# From darkness to light: Genetic manipulation of an atypical plant virus unveils key insights into kitavirus biology, highlighting capsid protein and eIF4A engagement to drive viral infection

**DOI:** 10.1371/journal.ppat.1013388

**Published:** 2025-08-01

**Authors:** Mikhail Oliveira Leastro, Elliot Watanabe Kitajima, Vicente Pallas, Jesús A. Sánchez-Navarro

**Affiliations:** 1 Department of Stress Biology, Institute of Molecular and Cellular Biology of Plants, CSIC- Universitat Politècnica de València, Valencia, Spain; 2 Department of Phytopathology and Nematology, University of Sao Paulo, Luiz de Queiroz College of Agriculture, Piracicaba, Brazil; University of Cambridge, UNITED STATES OF AMERICA

## Abstract

*Kitaviridae*, a newly recognized virus family, includes plant viruses infecting crops of great global importance, notably citrus. Despite its significant impact on citrus agricultural production, the molecular mechanisms underlying kitavirus infections remain largely unknown. Here, we engineered a recombinant citrus leprosis virus C (CiLV-C, genus *Cilevirus*) expressing green fluorescent protein (GFP) and demonstrated its feasibility for studying the biology of cilevirus. Genetic manipulation of rCiLV-C-GFP revealed that vRNA1 is essential for replication and can self-replicate independently, while vRNA2 is crucial for movement. The intergenic region between the polymerase and capsid protein (CP) acts as a promoter for CP gene expression. Frameshift and deletion analyses provided key insights into replication, movement, and morphogenesis. We reported that CP is critical for viral RNA accumulation, while movement protein (p32) facilitates viral spread. The putative glycoprotein (p61) is not structurally essential, as its deletion did not affect virion assembly, whereas the putative matrix protein (p24) is critical for morphogenesis, likely acting as a structural protein. Deletion of the RNA silencing suppressor (RSS, p15) and p15-p61 attenuated symptoms, implicating them as virulence factors. Additional analyses revealed that CP enhances vRNA accumulation through a mechanism independent of RSS. CP exhibits RNA-binding properties and interacts with eukaryotic initiation factor 4A (eIF4A), suggesting a role in translation. Overexpression of eIF4A increased CiLV-C RNA accumulation, while eIF4A knockdown reduced it, indicating that CP may recruit eIF4A to promote replication. Similar results were observed with turnip crinkle virus (TCV), and notably, the TCV CP efficiently restored RNA accumulation of a CP-defective CiLV-C, suggesting the existence of a conserved, CP-dependent, replication-related mechanism shared across distinct virus families. Our findings support the proposal of an initial model that elucidates the mechanism through which the CPs drive the production of high levels of vRNA manipulating host eIFs.

## Author summary

This study provides a comprehensive molecular characterization of a kitavirus using a recombinant citrus leprosis virus C (rCiLV-C) engineered to express GFP. The roles of the two vRNA segments and their encoded proteins in replication, movement, morphogenesis, and virulence were investigated. Our findings reveal that vRNA1 is required for autonomous replication, whereas vRNA2 is necessary for viral transport. The capsid protein (CP) is essential for vRNA accumulation, and the movement protein facilitates viral spread. The putative matrix protein p24 is required for morphogenesis, whereas the putative glycoprotein p61 is dispensable for virion assembly. Deletions of the RNA silencing suppressor p15 alone or in combination with p61 lead to symptom attenuation, highlighting their contribution to virulence. We also report an interaction between CP and the host eIF4A. Overexpression of eIF4A enhances vRNA accumulation, while its knockdown leads to reduced vRNA levels, suggesting that CP recruits eIF4A to promote viral replication. Similar CP-dependent effects were observed in a related betacarmovirus, indicating a potentially conserved replication mechanism across divergent virus families. By integrating genetic, molecular, and biochemical approaches, this work provides novel insights into kitavirus biology and highlights a novel role for CP in co-opting host translation machinery to enhance RNA accumulation.

## Introduction

Viruses are obligate parasites that necessarily rely on host cell machinery to develop their infectious cycle. In this regard, it is imperative and urgent to understand the molecular mechanism of virus-host interaction, particularly the host machinery hijacked by viruses to support their infection cycle. The family *Kitaviridae* is composed by single-stranded (ss) multiple-positive (+) sense RNA viruses with species assigned into the genera *Cilevirus*, *Higrevirus*, and *Blunervirus.* Most members of the family *Kitaviridae* are transmitted by tenuipalpid mites of the genus *Brevipalpus*, which have shown worldwide distribution, colonizing hundreds of plant species from several botanical families [1]. *Brevipalpus*-transmitted viruses (BTVs) are the causative agents of citrus leprosis (CL), the most economically important disease involving kitaviruses. This disease has a significant negative economic impact on the citrus industry [2]. CL manifests as nonsystemic chlorotic spots that typically progress into necrotic lesions on citrus trees’ leaves, branches, and fruits. Over time, the infection can lead to severe defoliation and dieback, ultimately causing the death of young citrus trees. The most extensively studied kitavirus is the type species of the genus *Cilevirus*, citrus leprosis virus C (CiLV-C; virus species *Cilevirus leprosis*). CiLV-C possesses a bi-segmented genome with 5’-cap and 3’-poly(A) structures. The six proteins encoded by cilevirus subgroup 1 (for more details about the taxonomy of cileviruses see [1]) are: the polymerase precursor (RdRp), containing conserved domains of methyl transferase, helicase, protease, and polymerase [3]; capsid protein (CP or p29; here after referred to as p29) [4,5]; a small protein (p15) that acts as a component of the viral RNA silencing suppression (RSS) machinery [6]; the movement protein (p32 MP) [7,8]; and putative glyco- (p61) and matrix- (p24) proteins [4,9]. The RdRp and p29 proteins are translated from RNA1, while p15, p61, MP, and p24 are translated from RNA2. Vesicles comprising enveloped bacilliform (40–70 nm x 100–120 nm) viral particles are extensively visualized in infected plant tissue [10], but discrepancies in size and morphology have been reported across different studies. Such variations may result from differences in sample preservation and preparation methods. Additionally, it is well established that many enveloped viruses alter their structural conformation during intracellular endosomal acidification [11], which may contribute to such morphological variations. During morphogenesis with the virus replication and assembly occurring in the cytoplasm, the particles accumulate in the lumen of the endoplasmic reticulum (ER).

While most plant viruses can spread systemically in their host resulting in a widespread and efficient invasion, a few exceptions are unable to achieve systemic dissemination, causing localized lesions where viral particles accumulate. Kitaviruses are notable for causing a locally restricted unusual infection, resulting in a limited or absent capacity to spread systemically in their natural and experimental hosts [2]. Experimental procedures confirmed the systemic movement capacity of kitavirus-encoded MPs [7], thereby excluding the potential inefficiency in the movement activity of these MPs as a limiting factor for systemic movement. In this regard, it is thought that the movement restriction is probably due to suboptimal interaction between the virus and its hosts [12], which limits viral replication and consequently its spread, at least for cileviruses.

The mechanisms for replication and virus genome expression utilized by positive-stranded RNA viruses vary extensively and may depend on structures associated with the 5’ and 3’ ends of the viral RNA (vRNA). The 5’ end may be naked, capped, or covalently linked to a viral protein and the 3’ end may be polyadenylated or highly structured naked forms [13]. Viruses typically use their untranslated regions (5’/ 3’ UTRs) to recruit components of the translation initiation machinery [14–17]. By orchestrating eukaryotic initiation factors (eIFs), viruses reprogram the protein synthesis machinery to favor their cycle of infection. In the cap-dependent translation initiation process, mRNA is recruited to a protein complex called eIF4F. This complex comprises the cap-binding protein eIF4E, the helicase eIF4A, the scaffold protein eIF4G - which interacts with the poly(A)-binding protein (PABP) - and eIF3, a multiprotein complex that promotes mRNA recruitment to the *43S* pre-initiation complex (PIC) and facilitates mRNA scanning for AUG codon recognition. In the translation process, eIF4E participates in the recruitment of mRNA to ribosomes for protein synthesis; eIF4A acts to unwind secondary structure in the 5’UTR, recruiting the PIC and allowing it to scan for start codons; and eIF4G, which in turn joins eIF4E and eIF4A and other initiation factors [18–21]. The binding of eIF4F to the cap structure induces mRNA cyclization, a key step for genome amplification of positive-stranded viruses [22]. Viral proteins have been constantly related in association with eIFs. For instance, the influenza A virus NS1 protein interacts with eIF4G and PABP to promote preferential translation of vRNA [23]. The protyvirus-derived viral genome-linked protein (VPg) associates with host eIF4E for successful infection [24]. In hantavirus, the eIF4A is substituted by the nucleocapsid protein (N), being able to differentiate between a viral and cellular mRNA, favoring vRNA, and consequently, the formation of new virions [25]. For the alfamovirus alfalfa mosaic virus (AMV), the CP stimulates the translation of viral RNA by acting as a functional analogue of PABP, assisting the vRNA circularization [26]. In the case of kitaviruses, no knowledge was generated about how these atypical viruses interact with the host’s translation machinery or the specific roles their viral proteins play in this critical process. The mysteries surrounding these interactions make kitaviruses an intriguing subject for further research, with the potential to uncover new mechanisms that could improve our understanding of virology.

Unfortunately, the lack of a reverse genetic system has left many aspects of replication, morphogenesis, and movement poorly understood for this atypical family. To overcome this limitation, we have recently developed a critical molecular tool to advance our understanding of kitavirus biology: an infectious clone of the CiLV-C [27]. This construct is pivotal for enhancing our understanding of the mechanisms employed by the virus to establish infection.

In this investigation, we have engineered a replication-competent virus that stably expresses green fluorescent protein (GFP) and demonstrated its feasibility for studying the biology of CiLV-C. Mutational and deletion analyses with recombinant CiLV-C have provided key insights into CiLV-C replication, movement, and morphogenesis allowing us to clarify fundamental questions that intrigue the biology of an atypical plant virus. Moreover, we demonstrated that the efficient accumulation of CiLV-C RNAs in plant cells depends on the capsid protein expression. The mechanism by which p29 operates is not directly associated with its RNA silencing suppressor activity but likely involves its ability to interact with RNA and host translation initiation factors. Our findings support the proposal of an initial model that elucidates the mechanism through which the capsid proteins drive the production of high levels of vRNA.

## Results

### A recombinant CiLV-C (rCiLV-C) vector stably expresses a foreign reporter gene and reveals CiLV-C RNA1 independence in replication and the role of RNA2 in movement

**Fig 1:**
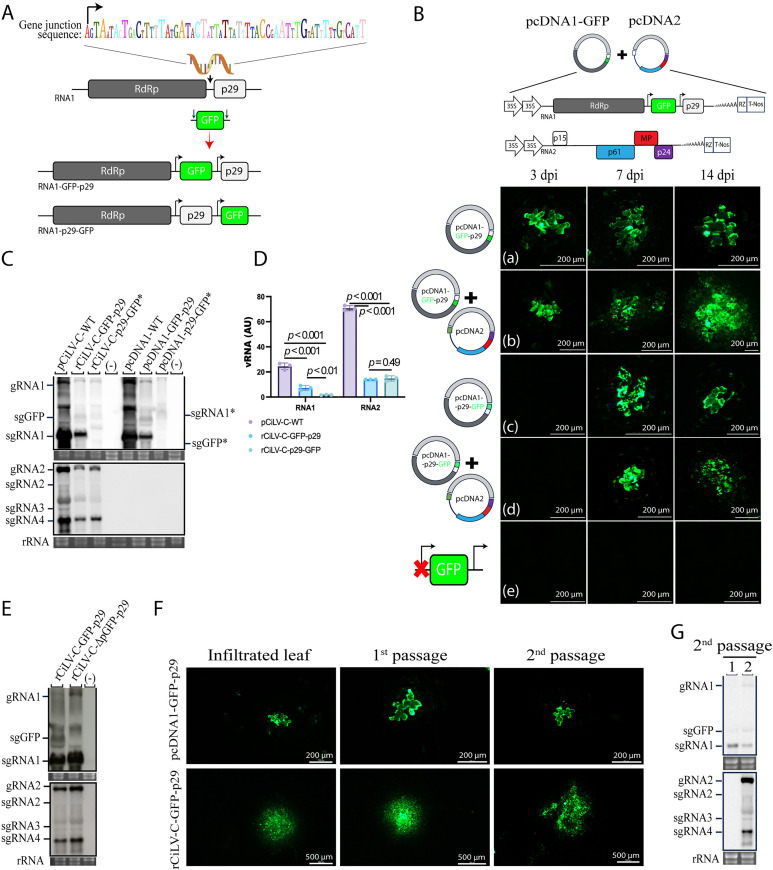
A recombinant CiLV-C vector expressing a foreign GFP gene (rCiLV-C-GFP) highlights the role of CiLV-C RNA1 in viral replication and RNA2 in viral movement. (A) Schematic representation of GFP insertion upstream (RNA1-GFP-p29) and downstream (RNA1-p29-GFP) of the *p29* gene in CiLV-C RNA1. The recombinant vector transcribes *GFP* gene under the control of a duplicated poly/p29 gene junction (GJ) sequence (black arrows) localized upstream of the GFP and p29 genes. (B) GFP fluorescence signal at 3-, 7- and 14-dpi from *Nicotiana benthamiana* leaves (four leaves per plant in three independent assays) agroinfiltrated with individual *Agrobacterium* culture carrying the pcDNA1-GFP-p29 (a), pcDNA1-p29-GFP (c) or bacterial mixture carrying pcDNA1-GFP-p29 + pcDNA2 (rCiLV-C-GFP-p29) (b) and pcDNA1-p29-GFP + pcDNA2 (rCiLV-C-p29-GFP) (d). (e) correspond to leaves infiltrated with a variant of pcDNA1-GFP-p29 in which the upstream GFP GJ sequence has been deleted (pcDNA1-ΔpGFP-p29). Scale bars correspond to 200 μm . (C) Northern blot showing CiLV-C RNA1 and RNA2 accumulation at 6 dpi, from *N. benthamiana* leaves agroinfiltrated with pCiLV-C-WT, rCiLV-C-GFP-p29, rCiLV-C-p29-GFP, pcDNA1-WT, pcDNA1-GFP-p29, and pcDNA1-p29-GFP using a DIG-labeled riboprobe complementary to the CiLV-C *p29* and *p24* genes. (-) corresponds to a non-infected plant. Asterisks (*) indicate the subgenomic viral RNAs (sgRNA1 and sgGFP) derived from the pcDNA1-p29-GFP construct. Ethidium bromide-stained rRNA serves as a loading control. The localization of CiLV-C gRNA1 and 2, as well as sgRNA1, 2, 3, 4, and sgGFP, is indicated. (D) Graphs represent the mean data from three independent biological replicates, showing the relative accumulation (arbitrary units, AU) of total CiLV-C RNAs. Error bars represent SD. Unpaired student’s t-test (two-tailed) with *p*-values is shown. The *p*-value < 0.05 is considered statistically significant. (E and G) Northern blot analysis at 6 dpi, using a DIG-labeled riboprobe complementary to the CiLV-C *p29* and *p24* genes. RNA band intensity was quantified using Fuji software with the ISAC plugin. Lane 1 corresponds to RNA sample obtained from leaves infected with pcDNA1-GFP-p29, and lane 2 from leaves infected with rCiLV-C-GFP-p29. (F) Loupe fluorescence images from *N. benthamiana* leaves agroinfiltrated with pcDNA1-GFP-p29, rCiLV-C-GFP-p29, and mechanically inoculated with infectious leaf extracts from the agroinfiltrated leaves (1^st^ passage) and subsequently from mechanically inoculated leaves (2^nd^ passage). Scale bars correspond to 200 μm and 500 μm.

Reporter genes have proved to be an excellent tool for understanding viral disease progression. To engineer a rCiLV-C vector for foreign gene expression in plants, we adapted the polymerase/p29 gene junction (GJ) with the GFP coding sequence and inserted it between the polymerase and p29 genes in the CiLV-C RNA1. The GJ sequence was inserted either upstream or downstream of the p29 coding sequence (Fig 1A) to generate pcDNA1-GFP-p29 and pcDNA1-p29-GFP. *Nicotiana benthamiana* leaves were infiltrated with both individual *Agrobacterium* cultures carrying the pcDNA1-GFP-p29 or pcDNA1-p29-GFP, as well as with a bacterial mixture harboring pcDNA1-GFP-p29 or pcDNA1-p29-GFP along with the other viral genomic component (pcDNA2) (rCiLV-C-GFP-p29 and rCiLV-C-p29-GFP). At 3 days post-inoculation (dpi), for the leaves expressing the construct harboring the GFP inserted upstream of the p29 gene (rCiLV-C-GFP-p29), the GFP expression was visualized in individual cells distributed throughout the infiltrated tissue. Progressing to fluorescence of the neighboring cells at 7 dpi, and the complete foci formation containing several GFP cells was noticed at 14 dpi (Fig 1B, panel b). No foci formation was observed at 7 or 14 dpi, with only individual GFP cells visualized (Fig 1B, panel a) for *N. benthamiana* leaves expressing only pcDNA1-GFP-p29. For the construct carrying the GFP downstream of the p29 gene (rCiLV-C-p29-GFP), individual GFP cells began to be visualized at 7 dpi (Fig 1B, panel c and d) with the presence of small foci at 14 dpi (Fig 1B, panel d). As expected, leaves infiltrated with pcDNA1-p29-GFP separated from pcDNA2, did not exhibit GFP foci formation at 14 dpi (Fig 1B, panel c). These results indicate that while the CiLV-C RNA1 genomic segment is sufficiently independent to self-replicate, it requires components of the second genomic segment for its cell-to-cell movement.

Considering the efficiency in foci formation, the construct expressing GFP between the polymerase and p29 genes seems to have a lesser impact on viral fitness compared to the version expressing the GFP downstream of the p29 gene, which exhibited a 4-day delay in the onset of GFP expression and the formation of infection foci. To gain further insight into the impact of a reporter gene on viral fitness, we compared the vRNA accumulation between the rCiLV-C-GFP vectors. Northern blot analyses (Fig 1C) revealed higher vRNA1 accumulation from the infection with the rCiLV-C-GFP-p29 vector compared to the rCiLV-C-p29-GFP (Fig 1D, *p*-value < 0.001). However, no major impact was noticed for the vRNA2 (*p*-value 0.49). Although the pcDNA1-GFP-p29 construct has a lesser impact on viral fitness compared to the pcDNA1-p29-GFP version, it is important to note that the presence of a reporter gene in the CiLV-C genome markedly decreases viral accumulation relative to the non-recombinant CiLV-C wild-type (WT) (Fig 1 C and D). Specifically, there is more than a 3.39-fold decrease in RNA1 accumulation and a 5.94-fold decrease in RNA2 accumulation in the rCiLV-C-GFP-p29 compared to the WT (pCiLV-C-WT). These differences persist both in the presence or absence of vRNA2 (Fig 1C).

The finding that the duplication of polymerase/p29 GJ allows the functional expression of a foreign gene strongly suggests that the sequence upstream of the p29 ORF (62 nt GJ) must act as a promoter. To test this hypothesis, we deleted the 62 nt GJ sequence (5’-AGTAATACTGACTTTTATGATACTATTATTGATATTTTACCGAATTTGTATTTTGTCATT-3’) upstream of the GFP gene from the pcDNA1-GFP-p29 construct, generating the pcDNA1-ΔpGFP-p29 vector. No GFP signal was visualized in the *N. benthamiana* leaves infiltrated with rCiLV-C-ΔpGFP-p29 (pcDNA1-ΔpGFP-p29 + pcDNA2) (Fig 1B, panel e). However, at 7 dpi, northern blot analysis confirmed the replication of the recombinant virus rescued from the rCiLV-C-ΔpGFP-p29 vector by detecting the subgenomic RNAs (sgRNAs) from CiLV-C RNA1 and 2. Notably, the synthesis of GFP subgenomic RNA transcripts (sgGFP) was not detected during rCiLV-C-ΔpGFP-p29 infection (Fig 1E). In contrast to the sample expressing the non-deleted version (rCiLV-C-GFP-p29), where the sgGFP band was clearly visible (Fig 1E). To determine whether the 62-nt GJ corresponds to the 5′ end of the sgRNA encoding p29, we performed a RACE analysis from the vcRNA. Total RNA extracted from plants infected with CiLV-C and rCiLV-C-GFP was both tailed with poly(G) and poly(U), followed by RACE using poly(C) and poly(A) primers in combination with virus-specific primers. Sanger sequencing revealed that the subgenomic RNA encoding CiLV-C p29 contains the full 62-nt GJ region plus 34 nt derived from the polymerase gene. In contrast, the sgGFP RNA contains only 48 nt of the GJ region (S1 Fig). These results indicate that the GJ region upstream of the p29 ORF contains essential promoter elements required for the transcription of downstream subgenomic RNAs. This is supported by the absence of GFP expression and sgGFP transcripts when the entire 62-nt GJ sequence was deleted, while the detection of sgGFP containing only 48 nt of the GJ suggests that a shorter portion of this region is sufficient to retain promoter activity.

Plant positive-strand RNA vectors are unable to stably maintain foreign genes during plant-to-plant transfers, with only a few rare exceptions [28–30]. To assess the stability of pcDNA1-GFP-p29, *N. benthamiana* plants were mechanically inoculated with leaf extracts infected with rCiLV-C-GFP-p29 (pcDNA1-GFP-p29 + pcDNA2) or pcDNA1-GFP-p29, and GFP expression was monitored under UV light and northern blot analysis. GFP-cell signals and GFP-foci fluorescence were extensively visualized following two serial passages in leaves infected with pcDNA1-GFP-p29 and rCiLV-C-GFP-p29, respectively (Fig 1F). Northern blot analysis demonstrated that the GFP insert was stably maintained in the progeny virus genome, as indicated by the detection of the sgGFP after two viral passages (Fig 1G). These findings demonstrate that CiLV-C RNA1 can be adapted for stable expression of foreign genes.

### Tracking cilevirus infection in leaf tissue using a rCiLV-C-GFP virus

**Fig 2:**
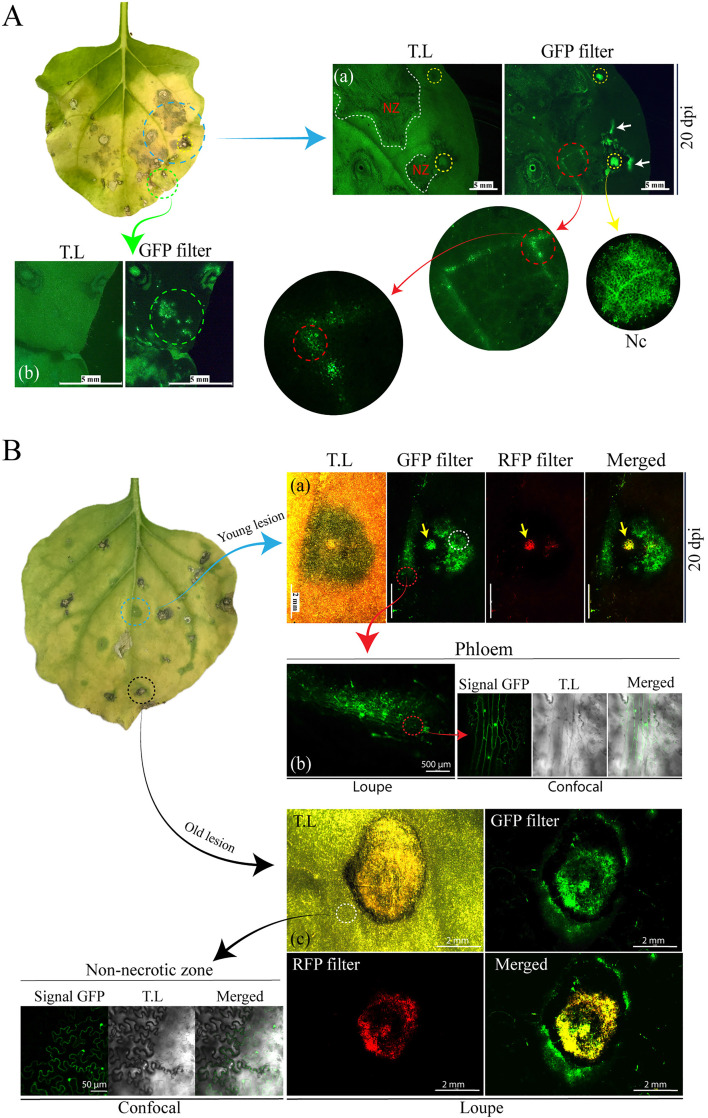
Tracking the CiLV-C infection by assessing GFP expression in plant leaf tissue. (A) *N. benthamiana* leaf agroinfiltrated with rCiLV-C-GFP constructs at 20 dpi. The image shows a necrosis lesion distributed through the infiltrated tissue. (a) A magnified view of the area indicated by the blue circle reveals large necrotic zones (NZ) outlined with white dotted lines, and individual necrotic lesions (typical cilevirus symptoms) with a necrotic center (Nc) (highlighted with yellow circles) showing intense autofluorescence under UV light. A magnification of a Nc provides a more detailed view of the necrosis. Red dotted circles indicate magnified images of the GFP signal in vein tissue. GFP-foci signal is also visualized surrounding the necrotic lesion (white arrows). (b) The green circle highlights a non-necrotic zone with GFP-foci that are not associated with necrotic lesions. (B) *N. benthamiana* leaf mechanically inoculated with leaf extract infected with rCiLV-C-GFP, at 20 dpi. Magnified images from young (a, b) and old (c) lesions show a detailed view of CiLV-C distribution in infected plant tissue. Yellow arrows indicate the Nc of a necrotic lesion. White and red circles highlight GFP signals around the necrotic lesion (a and c) and within the vein tissue (b), respectively. Confocal image reveals GFP signal in the cytoplasm and nucleus of parenchymal phloem cells (b) and in the epidermal cells of a non-necrotic zone (c). Fluorescence loupe images show the transmitted light (TL), GFP filter, RFP filter, and merged images. Confocal images display green (GFP), transmitted light (TL) channels, and merged images. Scale bars correspond to 50μm, 500 μm, 2 mm, and 5 mm.

Previous studies indicate that cileviruses cause only localized lesions, with virus-like particles restricted to the areas surrounding the lesions and absent in asymptomatic areas [2]. Taking advantage of the pcDNA1-GFP-p29 vector (hereafter referred to as pcDNA1-GFP), which carries the GFP reporter gene, we tracked virus distribution in both infiltrated and mechanically inoculated tissue by assessing GFP expression. At 20 dpi, *N. benthamiana* leaves agroinfiltrated with pcDNA1-GFP + pcDNA2 (rCiLV-C-GFP) showed several necrotic areas not only restricted to localized lesions (Fig 2A, blue circle in leaf image and Fig 2A, panel a, white dashed line). Under UV light, leaves exhibited multiple GFP foci distributed around localized lesions (Fig 2, panel a, white arrows) and more expressively throughout the leaf tissue, indicating their presence was not restricted to the necrotic lesions (green circle, Fig 2A, panel b). The regions lacking necrosis but exhibiting GFP foci remained unchanged throughout the entire observation period (30 dpi). GFP signal was also visualized in the leaf veins (Fig 2A panel a, red circle), suggesting that the virus can reach the leaf vascular tissue, which disagrees with the hypothesis that BTVs seem unable to reach the phloem. During hypersensitive cell death (HR), phenolics are released by decompartmentalization following cell death and accumulate in the dead cell’s wall area, causing autofluorescence [31]. To distinguish GFP autofluorescence from virus-induced GFP expression, we included fluorescence images acquired using a red fluorescent protein (RFP) filter, which specifically detects the autofluorescence signal derived from the necrotic lesions. In the necrotic center (Nc) of the localized lesions (yellow circles in Fig 2A, panel a; and yellow arrow in Fig 2B, panel a), intense autofluorescence was detected under different filters (GFP and RFP) (RFP filter is shown only in Fig 2B).

Unlike the widespread necrotic phenotype observed in agroinfiltrated leaves, mechanically inoculated leaves, at 20 dpi, displayed necrotic lesions confined to specific localized areas (Fig 2B, leaf image blue and black circles). Similar to those reported in the natural context of cilevirus infection [2], the virus distribution monitored by GFP expression was restricted to the areas surrounding the lesion (white circles, Fig 2B, panels a and c). Confocal images provide a detailed view of GFP distribution in both the nucleus and cytoplasm of infected cells around the non-necrotic zones (Fig 2B, panel c confocal). The GFP signal was not visualized in asymptomatic areas. To monitor the progression of the infection, we compared a young lesion (Fig 2B, panel a) with an older lesion (Fig 2B, panel c). The pattern of virus dissemination was consistently confined to the areas surrounding the lesions (white circles, Fig 2B, panels a and c). Autofluorescence in Nc was extensively visualized, progressing from small to larger areas as the lesion advanced. Similar to the observation in agroinfiltrated leaves, the virus distribution was also visualized in the leaf veins (Fig 2B, panel b, loupe) with GFP signal present in both the nucleus and cytoplasm of phloem parenchyma cells (Fig 2B, panel b, confocal). After 30 days post-inoculation, the GFP fluorescence remained around the necrotic zone and was not visualized beyond the inoculated leaf.

### The CiLV-C requires the capsid protein for efficient vRNA accumulation and p32 (MP) for cell-to-cell movement

**Fig 3:**
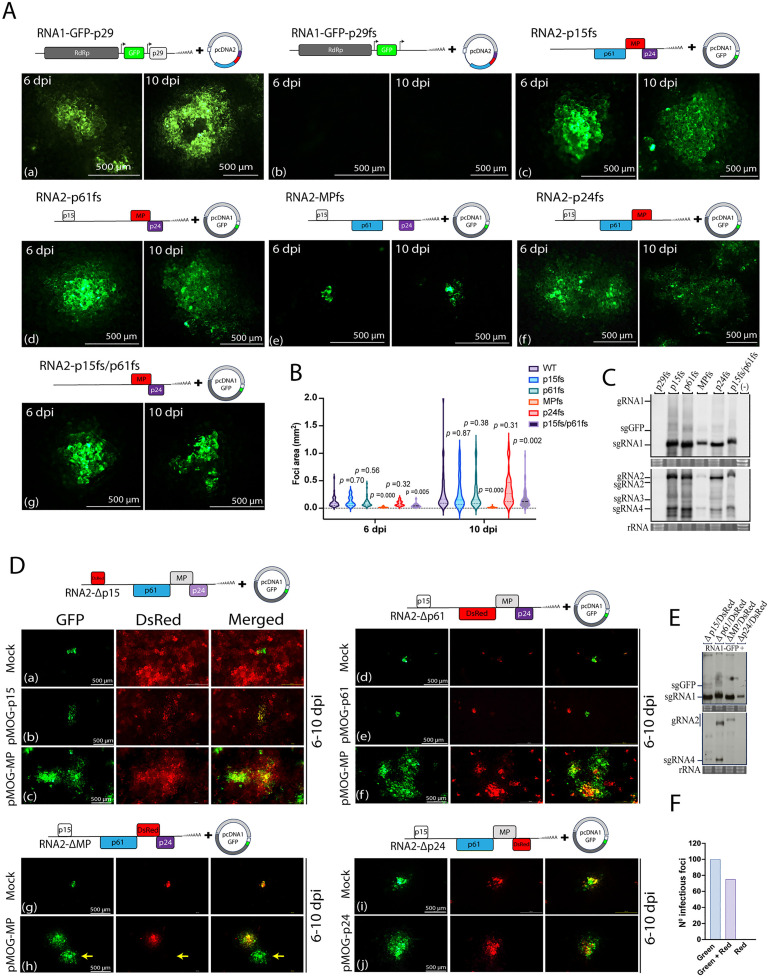
Effect of frameshift mutations and gene deletion on CiLV-C movement and RNA accumulation. (A) Cell-to-cell movement analysis of rCiLV-C-GFP (a) and its derivative constructs carrying frameshift mutation in p29 (b), p15 (c), p61 (d), MP (e), and p24 (f) genes. (g) corresponds to a double mutant (p15/p61). At the top of each panel a schematic genomic representation of rCiLV-C-GFP and its frameshift-mutated version is shown. The GFP signal in *N. benthamiana* leaves was captured at 6 and 10 dpi using a fluorescence loupe. Scale bar, 500 μm. (B) Violin plots represent the area measurement (mm^2^) of 14-100 independent infection foci at 6 and 10 dpi. Statistical analysis was performed using an unpaired two-tailed Student’s t-test, with significance set at *p* < 0.05. (C and E) Northern blot analysis showing CiLV-C RNA1 and RNA2 accumulation at 6 dpi, from *N. benthamiana* leaves agroinfiltrated with rCiLV-C-GFP and its frameshift (C) and deleted (E) versions using a DIG-labeled riboprobe complementary to the CiLV-C *p29* and *p24* genes. (-) corresponds to a non-infected plant. Ethidium bromide-stained rRNA serves as a loading control. The localization of CiLV-C gRNA1 and 2 and their respective subgenomic RNAs (sgRNA1, sgRNA2, sgRNA3, and sgRNA4), including the sgGFP, are indicated. (D) *N. benthamiana* leaves were infiltrated with pcDNA1-GFP along with constructs in which the entire transcriptional unit was deleted: Δp15 (a), Δp61 (d), ΔMP (g), and Δp24 (i). Panels b, c, e, f, h, and j correspond to trans-complementation cell-to-cell movement assays using rCiLV-C-Δp15/DsRed with pMOG-p15 and pMOG-MP, rCiLV-C-Δp61/DsRed with pMOG-p61 and pMOG-MP, rCiLV-C-ΔMP/DsRed and rCiLV-C-Δp24/DsRed with pMOG-MP and pMOG-p24. GFP, DsRed, and merged images were captured at 6-10 dpi. Scale bar, 500 μm. Yellow arrows indicate the presence of GFP-foci expressing only CiLV-C RNA1. (F) Graph representing the count of 100 fluorescence foci from three independent biological replicates from leaves infiltrated with rCiLV-C-ΔMP/DsRed vector. Green indicates the number of foci expressing only the GFP signal from the recombinant CiLV-C RNA1-GFP, Red represents the number of foci expressing the red fluorescent signal from the recombinant CiLV-C RNA2-DsRed, and Green + Red indicates the number of foci expressing both green and red fluorescent signals.

Our next step was to investigate the involvement of each CiLV-C-encoded protein in viral accumulation and movement. To knockdown p29, p15, p61, MP, and p24 genes in the rCiLV-C-GFP vector, the gene-coding sequences were modified by introducing frameshift mutations. *Agrobacterium* carrying the p29 (pcDNA1-GFP-p29fs), p15 (pcDNA2-p15fs), p61 (pcDNA2-p61fs), MP (pcDNA2-MPfs), and p24 (pcDNA2-p24fs) frameshift mutants were each agroinfiltrated into *N. benthamiana* leaves along with the corresponding viral genomic component (pcDNA1-GFP or pcDNA2), and their requirement for virus replication and movement were compared to those of rCiLV-C-GFP non-mutated (wild type, WT). In rCiLV-C-GFP-WT agroinfiltrated leaves, GFP-foci cells were visualized at 6 dpi and spread extensively into adjacent cells by 10 dpi (Fig 3A, panel a). However, the leaves infiltrated with rCiLV-C-GFP-p29fs (pcDNA1-GFP-p29fs + pcDNA2) did not present GFP signal (Fig 3A, panel b). To investigate whether the absence of p29 affects the GFP visualization

by impairing CiLV-C replication, we analyzed the vRNA accumulation and sgRNA formation by northern blot assay. At 6 dpi, the vRNA accumulation was not detected from the leaves infiltrated with rCiLV-C-GFP-p29fs (Fig 3C). These findings suggest that the CiLV-C capsid protein may have an essential role in virus replication. In leaves expressing rCiLV-C-GFP-MPfs (pcDNA1-GFP + pcDNA2-MPfs), GFP expression was restricted to individual cells at both 6 and 10 dpi (Fig 3A, panel e). This result confirms that movement protein p32 plays a crucial role in the virus movement [7]. In contrast, rCiLV-C-GFP-p15fs (pcDNA1-GFP + pcDNA2-p15fs), rCiLV-C-GFP-p61fs (pcDNA1-GFP + pcDNA2-p61fs), and rCiLV-C-GFP-p24fs (pcDNA1-GFP + pcDNA2-p24fs) mutants were functional in cell-to-cell movement (Fig 3A, panels c, d, and f), ensuring a local movement similar to the rCiLV-C-GFP (*p* value > 0.05, Fig 3B). On the other hand, a viral variant carrying a double fs mutation (p15 and p61) (rCiLV-C-GFP-p15fs/p61fs) (Fig 3A, panel g) exhibits reduced movement compared to rCiLV-C-GFP WT (*p* value < 0.05, Fig 3B). Northern blot analysis (at 6 dpi) indicates that the vRNA accumulation was comparable among the constructs, except for rCiLV-C-GFP-MPfs with DIG-signal of both RNAs 1 and 2 less intense (Fig 3C).

To evaluate the impact of the entire transcript unit of the CiLV-C genes on viral movement and to further confirm that the p32 protein, rather than the RNA sequence, is required for local movement, we replaced the full coding sequence of the CiLV-C RNA2-encoded proteins with the gene for red fluorescent protein (DsRed). At 6–10 dpi, in rCiLV-C-GFP-Δp15/DsRed (pcDNA1-GFP + pcDNA2-Δp15/DsRed) infiltrated leaves, individual GFP-cells were visualized, indicating that the RNA1 cannot be spread cell-to-cell (Fig 3D, panel a). Note that the DsRed signal was extensively visualized throughout the infiltrated tissue, indicating that the p15 is likely translated directly from the gRNA2. In rCiLV-C-GFP-Δp61/DsRed (pcDNA1-GFP + pcDNA2-Δp61/DsRed) and rCiLV-C-GFP-ΔMP/DsRed (pcDNA1-GFP + pcDNA2-ΔMP/DsRed) infiltrate leaves, only individual GFP-and DsRed-cells were visualized at 6–10 dpi (Fig 3D, panels d and g, respectively). While leaves expressing rCiLV-C-GFP-Δp24/DsRed (pcDNA1-GFP + pcDNA2-Δp24/DsRed), GFP-and DsRed-foci were visualized throughout the infected leaves (Fig 3D, panel i). Some of these results disagree with the analysis of frameshift mutation and raise the possibility that like MP, the p15 and p61 could play a role in virus movement. To address this question, we expressed the recombinant CiLV-C deletion constructs (Δp15, Δp61, and ΔMP) along with their respective deleted proteins *in trans*. We reasoned that if these proteins are involved in local movement, recovery of movement should be observed. The leaves expressing rCiLV-C-GFP-Δp15/DsRed + pMOG-p15 (Fig 3D, panel b) and rCiLV-C-GFP-Δp61/DsRed + pMOG-p61 (Fig 3D, panel e), fluorescent-foci formation was not visualized with only individual epidermal cells expressing GFP . This contrasts with the leaves expressing rCiLV-C-GFP-ΔMP/DsRed + pMOG-MP, which presented the formation of GFP/DsRFP-foci (Fig 3D, panel h). When rCiLV-C-GFP-Δp15/DsRed and rCiLV-C-GFP-Δp61/DsRed were co-expressed with pMOG-MP, the cell-to-cell movement was successfully rescued. This was confirmed by the visualization of GFP and DsRed foci, which indicated that both RNA1 and RNA2 were able to move (Fig 3D, panels c and f). A northern blot confirmed the replication of all these constructs (Fig 3E). Taken together, these results indicate that the complete coding sequences of p15 and p61 are essential for the proper processing of the movement protein, since their deletion impaired the viral movement, suggesting that these ORFs may share fundamental elements for gene transcription into their transcript unit. Alternatively, the replacement of these genes with DsRed might have affected the expression of downstream genes.

To gain further insights into the MP’s influence on CiLV-C movement, we tested whether this protein, when displaced of the CiLV-C RNA2, could facilitate cell-to-cell movement of the vRNA1 component. *N. benthamiana* leaves were co-infiltrated with a mix of bacterial cultures harboring the pcDNA1-GFP plus pMOG-MP:HA. As a control, leaves were infiltrated with cultures containing only pcDNA1-GFP. Throughout the infection (3–10 dpi), only individual GFP-cells were visualized in the leaves expressing pcDNA1-GFP (S2A Fig), in contrast with the leaves infiltrated with pcDNA1-GFP and the MP protein *in trans*. These leaves presented multiple GFP-foci, starting at 6 dpi and spreading extensively by 10 dpi (S2A Fig). Western blot analysis confirmed the MP and GFP expression (S2B Fig).

Considering that the CiLV-C RNA1 genomic component does not rely on RNA2 for replication (see northern blot analysis in Fig 1), we tested whether MP inserted into RNA1 (*cis*-expression) could guarantee the RNA1 movement. To this end, we modify the pcDNA1-GFP vector replacing the GFP gene with MPs of CiLV-C and tobacco mosaic virus (TMV). Northern blot analysis confirmed the pcDNA1-CiLV-C MP and pcDNA1-TMV MP replications by the detection of the sgRNAs. However, the vRNA accumulation was lower when compared with the pcDNA1-WT (S3A Fig). Notably, in leaves expressing pcDNA1-TMV MP, which carries a heterologous MP, the vRNA accumulation was higher than the construct carrying the CiLV-C MP. None of the non-infiltrated upper leaves were positive for virus detection, with viral cell-to-cell movement restricted to adjacent cells in the construct expressing the CiLV-C MP (pcDNA1-CiLV-C MP + pcDNA2ΔMP/GFP) (S3B Fig panel b). This result demonstrates that the insertion of MP into RNA1 preserves the ability for local movement. For the recombinant CiLV-C expressing the TMV MP (pcDNA1-TMV MP + pcDNA2ΔMP/GFP), the GFP signal was restricted to individual cells, as well as visualized in the control that does not express the MP (pcDNA1-WT + pcDNA2-ΔMP/GFP) (S3B Fig panels a and c, respectively). The sgRNA1, which likely corresponds to capsid protein transcripts [3], had a faint expression in the leaves infiltrated with recombinant vectors carrying the MPs.

### CiLV-C RNA2 obligatorily requires RNA1 to replicate

During the observation of the colocalization between the pcDNA1-GFP with pcDNA2-Δgene/DsRed (Fig 3D), we frequently noticed individual cells or foci expressing GFP exclusively (as indicated in Fig 3D, panel h, yellow arrows). Importantly, no cells exhibiting exclusive expression of DsRed were observed. To confirm this observation, we analyzed 100 infection foci from leaves infiltrated with rCiLV-C-GFP-ΔMP/DsRed + pMOG-MP. The foci were categorized into three groups: those exhibiting only green fluorescence, those exhibiting only red fluorescence, and those showing co-localization. All (100/100) of the foci exhibited RNA1 expression (GFP), with 75% (75/100) showing co-localization (GFP + DsRed), while none (0/100) displayed exclusive RNA2 expression (DsRed) (Fig 3F). This result confirms that the CiLV-C RNA2 requires RNA1 to replicate.

### Disruption of p61 expression does not impact CiLV-C virulence, unlike the disruptions of p15, MP, and p24 proteins

To assess the biological relevance of CiLV-C-encoded proteins, agroinfiltrated leaves with p29, p15, p61, MP, p24, and p15-p61 translational defect rCiLV-C constructs (Fig 3A), either carrying or lacking the GFP marker, were monitored for the development of leprosis symptoms. The leaves infiltrated with pCiLV-C-p29fs, pCiLV-C-MPfs, and their respective recombinant versions carrying GFP (rCiLV-C-GFP-ORFfs), did not show typical necrotic symptoms (S4 Fig). In contrast, at 10 dpi, the leaves infected with pCiLV-C-p15fs, pCiLV-C-p61fs, and pCiLV-C-p24fs, as well as their GFP-carrying versions, showed necrotic lesions that evolved into large necrosis distributed throughout the infiltrated tissue, resembling the symptoms seen in pCiLV-C-WT (S4 Fig). In the double mutation (p15fs/p61fs), although the infiltrated leaves generated symptoms, these were milder compared to wild-type CiLV-C at 10 dpi (S4 Fig).

**Fig 4:**
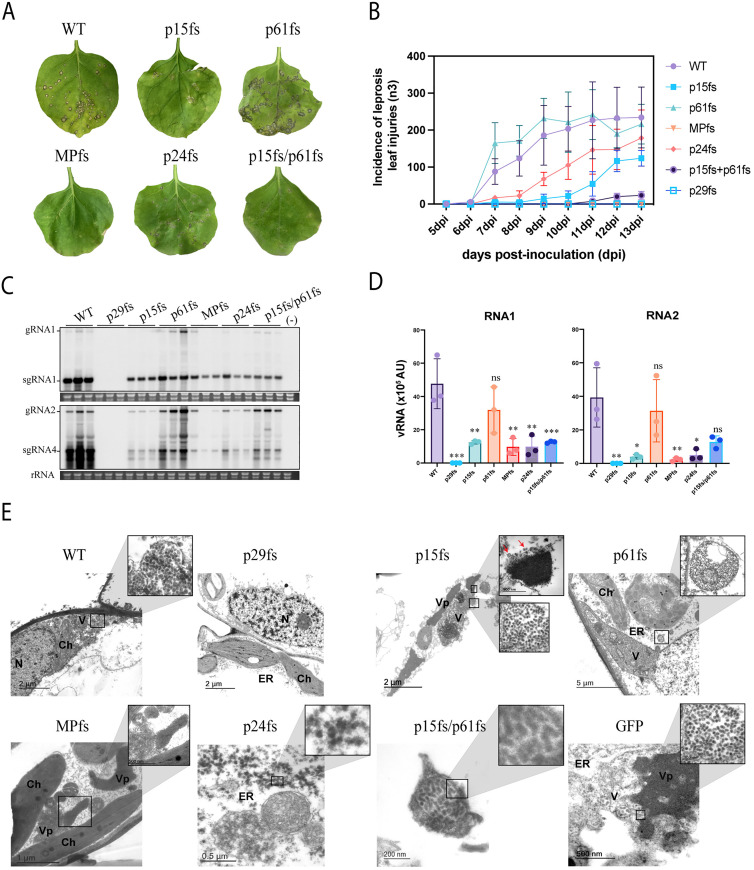
Impact of CiLV-C protein disruptions on symptom development, vRNA accumulation, and morphogenesis. (A) Symptom appearance in *N. benthamiana* leaves mechanically inoculated with pCiLV-C-WT and CiLV-C constructs carrying frameshift mutation in p29, p15, p61, MP, p24, and p15-p61, at 13 dpi. (B) The graphic represents the incidence of symptom appearance in *N. benthamiana* infected with pCiLV-C-WT and CiLV-C constructs carrying frameshift mutations mentioned above. Data are the mean of three independent biological replicates. Error bars represent SD. (C) Northern blot (at 6 dpi) using DIG-probes complementary to the CiLV-C p29 and p24. The localization of CiLV-C gRNA1, sgRNA1, gRNA2, and sgRNA4 are indicated. rRNA stained with ethidium bromide indicates equal loading of samples. (-) correspond to a non-infected plant. (D) Relative accumulation (arbitrary units, AU) of total CiLV-C RNAs 1 and 2 from three independent experiments of *N. benthamiana* leaves infected with pCiLV-C-WT and derivative versions carrying frameshift mutations. RNA band intensity was quantified from membranes shown in C using Fuji software with the ISAC plugin. Error bars represent SD. An asterisk indicates a statistically significant difference according to unpaired Student’s t-test (two-tailed), *** **p* *< 0.001, ** *p* < 0.01, * *p* < 0.05, and ns correspond to *p* > 0.05. AU, arbitrary units. (E) Electron micrographs of thin sections of *N. benthamiana* leaves infected with pCiLV-C-WT, pCiLV-C-p29fs, pCiLV-C-p15fs, pCiLV-C-p61fs, pCiLV-C-MPfs, pCiLV-C-p24fs, pCiLV-C-p15fs/p61fs, and rCiLV-C-GFP. The boxed sectors in the panels are magnified (30,000 X) to highlight CiLV-C virion particles or viroplasm structures. Enlarged images do not always precisely align with the delineated areas. Red arrows indicate vesicle structures containing fibrillar material emerging from viroplasm-like structures. Scale bars, 5 μm, 2 μm, 1 μm, 500 nm, and 200 nm. V, virion; Vp, viroplasm-like structure; N, nucleus; ER, endoplasmic reticulum; Ch, chloroplast.

In the course of infection, high necrosis was observed in the infiltrated areas, hindering the monitoring of virus symptoms. Therefore, to eliminate hypersensitive necrotic reactions generated by *Agrobacterium* [32], we assessed the symptoms in mechanically inoculated leaves using infiltrated tissue infected with mutant frameshift vectors lacking GFP (pCiLV-C-ORFfs) as the inoculum source. These symptoms were compared to those observed in leaves inoculated with the wild-type CiLV-C (pCiLV-C-WT). The incidence of leprosis leaf injuries (typical BTV symptoms) was measured and monitored for 13 days. At 6 dpi, leaves inoculated with pCiLV-C-p61fs exhibited the initial necrotic lesions, such as those observed in leaves infected with pCiLV-C-WT, and the incidence of leprosis injuries in the course of infection remained comparable to the WT (*p*-value > 0.05) (Fig 4A and 4B). However, a delay in symptom development was recorded on leaves infected with pCiLV-C-p24fs (1 day), pCiLV-C-p15fs (3 days), and pCiLV-C-p15fs/p61fs (5 days). MPfs mutant inoculation did not result in necrotic lesions (Fig 4A and 4B). As expected, leaves inoculated with pCiLV-C-p29fs do not generate necrotic injuries (Fig 4B). Note that disruption of p24 expression decreased the incidence of necrotic lesions in mechanically inoculated leaves, but not as much as disruption of p15 or the double mutation (p15fs/p61fs), which considerably reduced the incidence of symptoms (Fig 4A and 4B). Significant differences in the incidence of necrotic injuries were observed between p15fs, MPfs, p24fs, and p15fs/p61fs with WT (*p*-value < 0.001 for p15fs, MPfs and p15fs/p61fs with WT; and *p*-value < 0.02 for p24fs and WT) (Fig 4B).

Next, we also assessed the effect of the frameshift gene deletions in CiLV-C RNA1 and RNA2 accumulation compared with WT in mechanically inoculated leaves. At 7 dpi, no significant differences in RNA1 and RNA2 accumulation were detected between pCiLV-C-p61fs and pCiLV-C-WT (*p* > 0.25 for RNA1 and *p* > 0.62 for RNA2) (Fig 4C and 4D), which aligns with findings of leprosis injuries analysis (Fig 4B). Significant differences with low levels of vRNAs (RNA1 and RNA2) were reported for leaves mechanically inoculated with pCiLV-C-p15fs (*p* < 0.01 for RNA1 and *p* < 0.02 for RNA2), pCiLV-C-p24fs (*p* < 0.01 for RNA1 and *p* < 0.02 for RNA2), and pCiLV-C-p15fs/p61fs (RNA1) (*p* < 0.001) (Fig 4C and D). These results are consistent with the decreased incidence of leprosis injuries (Fig 4B). When comparing the vRNAs accumulation between pCiLV-C-WT and pCiLV-C-p15fs/p61fs (RNA2) (*p* > 0.06), no significant differences were observed (Fig 4C and 4D).

We also compared the accumulation of CiLV-C RNA1 and RNA2 from the mutated viruses in agroinfiltrated leaves (S4 Fig) with those mechanically inoculated (Fig 4). Northern blot analysis revealed that in both experiments, p29 frameshift deletion abolishes the vRNA expression to detectable levels (S5 Fig). In leaves agroinfiltrated with viruses carrying mutations in p15, p61, p24, and p15-p61, these frameshift deletions did not have a significant effect on virus replication (S5 Fig). However, when leaves were mechanically inoculated with extracts from leaves infiltrated with virus constructs carrying p15fs, p24fs, and p15fs/p61fs mutations, a significant decrease in CiLV-C RNAs levels was observed (except for RNA2 in p15fs/p61fs) (S5 Fig). In both agroinfiltrated and mechanically inoculated leaves, no significant differences in RNA1 and RNA2 accumulation were noticed between pCiLV-C-p61fs with pCiLV-C-WT (S5 Fig). In the case of MP, in agroinfiltrated leaves, RNA1 accumulation did not show significant differences compared to WT, unlike RNA2 (S5 Fig). This can be attributed to the self-replicative ability of RNA1. However, in mechanically inoculated leaves, a significant reduction in CiLV-C RNA1 levels was observed (S5 Fig). It is important to mention that low CiLV-C RNA accumulations in leaves infiltrated or sap-inoculated with pCiLV-C-MPfs (Figs 3D, 4D, and S5) correlate with the absence of symptoms, likely caused by the low number of adjacent cells infected due to restricted local movement (Fig 3A and 3C). Taken together, these data suggest that the disruption in p61 expression does not affect CiLV-C virulence (symptom and RNA accumulation) when assessed individually. On the other hand, p29, p15, MP, and p24 may play a role in CiLV-C virulence. Furthermore, these findings indicate a correlation between the onset of symptom attenuation and a reduction in viral RNA accumulation.

### The putative p24 matrix protein is required for morphogenesis, whereas the putative p61 glycoprotein does not seem to play a structural role in the mature virion particle

To address the effect of each mutation in virion morphogenesis, the infected tissue was inspected by transmission electron microscopy (TEM). Leaf tissue infected with pCiLV-C-WT was compared with pCiLV-C-p29fs, pCiLV-C-p15fs, pCiLV-C-p61fs, pCiLV-C-MPfs, pCiLV-C-p24fs, pCiLV-C-p15fs/p61fs, and rCiLV-C-GFP. As with previous studies of CiLV-C-infected plants [27,33], ER lumen of cells infected with pCiLV-C-WT contained enveloped bacilliform (mean virion 45.1 nm in length x 36.1 nm in width, n = 25) viral particles encompassed by vesicles (Fig 4E). In ultra-thin sections of tissues infected with pCiLV-C-p29fs, we were unable to identify structures reported as viral particles or electron-dense regions related to virus assembly and replication (viroplasm structures). Cells infected with pCiLV-C-p29fs displayed a normal phenotype (Fig 4E). In marked contrast, pCiLV-C-p15fs, pCiLV-C-p61fs, pCiLV-C-MPfs, pCiLV-C-p24fs, pCiLV-C-p15fs/p61fs, and rCiLV-GFP infected cells contained a large number of electron-dense regions characteristic of viroplasm structures within the cisternae of the ER (Figs 4E and S6). Regarding morphogenesis, mature enveloped virions were noticed in pCiLV-C-p15fs (mean virion 47.0 nm in length x 35.6 nm in width, n = 25), pCiLV-C-p61fs (47.6 nm in length x 34.7 nm in width, n = 25), pCiLV-C-p15fs/p61fs (46.9 nm in length x 29.7 nm in width, n = 12), and rCiLV-C-GFP (50.3 nm in length x 36.7 nm in width, n = 25) infected cells. In tissue infected with pCiLV-C-p15fs, vesicles containing fibrillar material - resembling those in which RNA replication is expected to occur - were extensively visualized around the edges of viroplasms, as previously observed in *Citrus* spp. (Elliot Kitajima, personal communication) (Fig 4E, red arrows). Although we did not observe mature particles in tissue infected with pCiLV-C-MPfs - possibly due to reduced viral RNA accumulation resulting from impaired viral spread - we cannot rule out the possibility of mature virions being present in this infected tissue, given the correct formation of viroplasm structures. In the case of cells infected with p24 frameshift deletion virus, aggregates of dense granular material were observed instead of compacted electron-dense structure, likely representing the same viroplasmic material that did not compact (Fig 4E). No mature and vesicle particles were visualized in this sample. Our data thus indicate that the p24 protein is required for morphogenesis and assembly of CiLV-C enveloped particles. The lengths of the viral particles were measured, and a comparison was conducted between pCiLV-C-WT, its fs-mutated variants, and rCiLV-GFP. Non-significant differences were observed in particle length between WT with p15fs, p61fs, and p15fs/p61fs (S7 Fig). On the other hand, rCiLV-C-GFP enveloped particles (mean virion length 50.3 nm) show approximately ~ 11.5% longer than the pCiLV-C-WT (45.1 nm) (**p* *< 0.001, S7 Fig), which is nearly proportional to the ~ 9.3% (9,557nt/8,745nt) increase in genome size. (S7 Fig). Importantly, given the bi-segmented nature of its RNA genome, we cannot exclude the possibility that virions may exist in a bipartite form, which could influence particle size measurements.

### Capsid protein enhances vRNA accumulation through a mechanism independent of RNA silencing suppression

**Fig 5:**
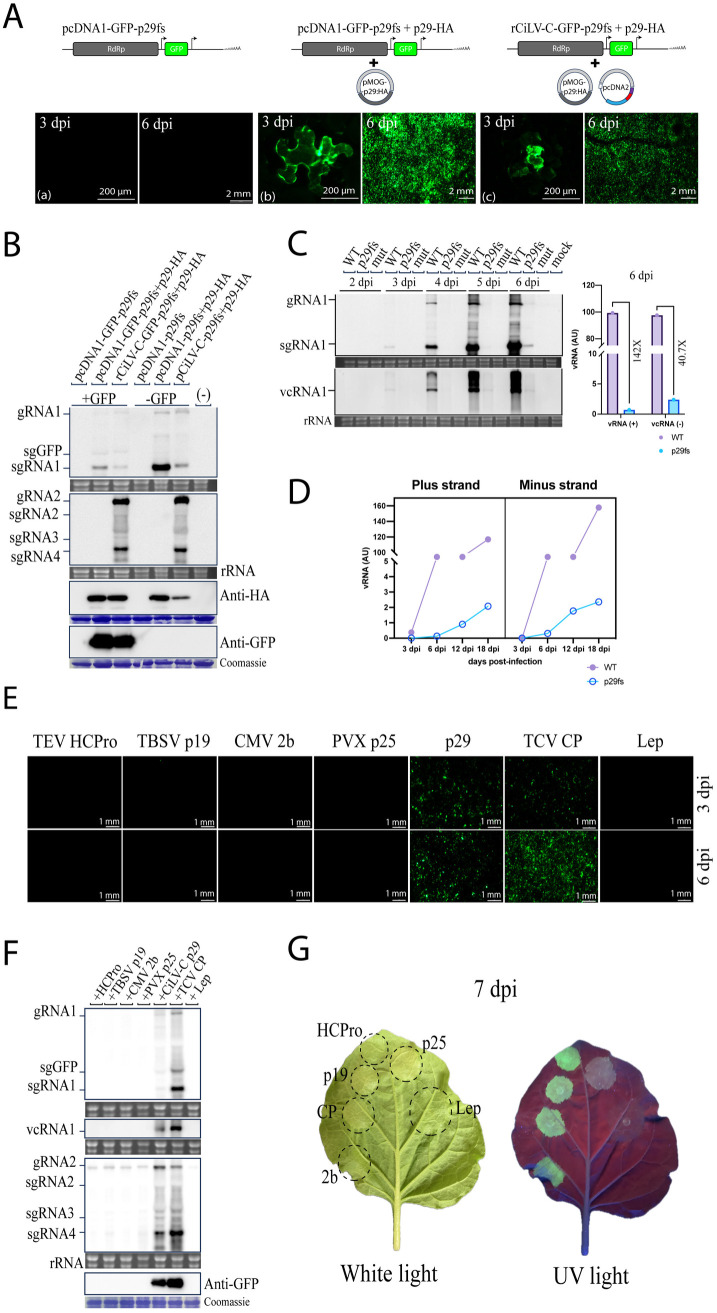
*Trans* expression of CiLV-C p29 and TCV CP can rescue a defective CiLV-C p29 clone. (A) Rescue of defective CiLV-C p29 clone (pcDNA1-GFP-p29fs) from *trans* expression of p29-HA. *N. benthamiana* leaves were infiltrated with individual agrobacterium carrying pcDNA1-GFP-p29fs (a) or in combination with pMOG-p29-HA (b) or pMOG-p29-HA plus pcDNA2 (rCiLV-C-GFP-p29) (c). A schematic genomic representation of pcDNA1-GFP-p29fs, pcDNA2 and pMOG binary vector, which carries the p29 targeted to the HA epitope at its C-terminus, is shown at the top of the panels. The GFP signal in *N. benthamiana* leaves was captured at 3 and 6 dpi using a fluorescence loupe. Scale bar, 200 μm and 2 mm. (B) Total RNA and proteins from *N. benthamiana* leaves shown in A, where the viral genome express the GFP (+GFP), or with the equivalent constructs without the GFP (-GFP), were analyzed at 6 dpi by northern and western blots, using a DIG-probe complementary to the CiLV-C p29 and p24, as well as antibodies against HA and GFP. The localization of CiLV-C gRNA1, gRNA2, sgRNA1-4, and sgGFP is indicated in the northern blot. Ethidium bromide-stained rRNA serves as a loading control. Proteins stained with Coomassie blue indicate equal loading of samples in the western blot. (-) corresponds to a non-infected plant. (C) A time course analysis of RNAs extracted from leaves inoculated with the wild-type version of CiLV-C RNA1 (pcDNA1-WT), the p29 defective CiLV-C RNA1 clone (pcDNA1-p29fs), and the pcDNA1-mut construct, which have a premature stop codon in the CiLV-C polymerase, preventing RNA replication. vRNA (plus strand) and vcRNA (minus strand) of CiLV-C RNA 1 were analyzed at 6 dpi by northern blot using DIG-probes complementary to the CiLV-C p29. The localization of CiLV-C gRNA1, sgRNA1, and vcRNA1 are indicated. Ethidium bromide-stained rRNA serves as a loading control. RNA band intensities were quantified using Fuji software with the ISAC plugin, and the data were represented graphically to compare the vRNA and vcRNA accumulation between pcDNA1-WT and pcDNA1-p29fs. (D) The graph illustrates the relative accumulation (arbitrary units, AU) of positive and negative strands of CiLV-C RNAs quantified by northern blot analysis at 3, 6, 12, and 18 dpi, using RNA extracted from leaves infected with pcDNA1-WT and pcDNA1-p29fs. (E) Analysis of the rescue of rCiLV-C-GFP-p29fs replication, indicated by GFP expression, through *trans* expression of the viral RNA silencing suppressor (RSS), including TEV HCPro, TBSV p19, CMV 2b, PVX p25, CiLV-C p29, and TCV CP. GFP signal was captured at 3 and 6 dpi. The leader peptidase protein (Lep), an integral component of bacterial membranes, was used as negative control. Scale bar, 1 mm. (F) Northern and western blot analysis of total RNA and proteins extracted from the leaves showed in E at 7 dpi. Northern blot analysis was performed using DIG-labeled riboprobes complementary to the CiLV-C p29 and p24, while western blot analysis was conducted using an antibody against the GFP. The localization of CiLV-C gRNA1, sgRNA1, vcRNA1, gRNA2, sgRNA2, sgRNA3, and sgRNA4 is indicated. (G) The functionality of the TEV HCPro, TBSV p19, CMV 2b, PVX p25, and TCV CP constructs was confirmed by evaluating their RSS activity through transient expression in wild-type *N. benthamiana* plants. A pMOG-eGFP construct was co-infiltrated with *Agrobacterium* suspension carrying a binary construct encoding the RSS proteins or Lep (control) ORFs. GFP fluorescence was monitored at 7 dpi.

Our findings, shown in Fig 3, reveal that p29 expression is essential for viral accumulation, suggesting its potential role in replication. To clarify the mechanism by which this protein contributes to this process, we first tested whether the *trans*-expression of p29 can rescue GFP expression of a defective recombinant CiLV-C-GFP containing an in-frame deletion of p29. *Trans*-complementation analysis revealed that the combination of pcDNA-GFP-p29fs with pMOG-p29 fused to an HA epitope (p29-HA), as well as the combination containing pcDNA2 (rCiLV-C-GFP-p29fs + p29-HA), generated a GFP signal at 3 dpi in the infiltrated leaves (Fig 5A, panels b and c). In contrast, leaves expressing only the p29 defective vector (pcDNA-GFP-p29fs) did not show visual GFP (Fig 5A, panel a). Northern and western blot analyses confirmed the vRNA replication through subgenomic detection, as well as the expression of GFP and p29-HA in leaves expressing the p29 *in trans* (Fig 5B). These results demonstrate that the p29 protein is able to rescue the infectivity of a defective CiLV-C clone and suggests a crucial role for p29 in virus viability.

To assess whether viral replication is fully impaired by the frameshift deletion of p29 or if it persists with reduced RNA accumulation, we evaluated minus-strand and plus-strand CiLV-C RNA accumulation in leaves infiltrated with pcDNA1-p29fs construct without GFP, which exhibits the highest accumulation (see Fig 1D). The second viral component was omitted because the presence of RNA2 might interfere with RNA1 replication by competing for polymerase during the replication process, potentially masking the detection of low levels of vRNA. Samples were collected at several early time points post-infection (2–6 dpi) and analyzed by northern blot. As controls, we used the pcDNA1-WT (positive control) and pcDNA1-mut (negative control), which have a premature stop codon in the CiLV-C polymerase that prevents vRNA replication [27]. Time course analysis demonstrates the presence of sgRNA and anti-genomic (complementary strand, vcRNA) RNA in leaves expressing pcDNA1-WT from 3 dpi, with signal intensity becoming more pronounced over subsequent days (Fig 5C). Notably, from leaves expressing the pcDNA1-p29fs construct, the presence of sgRNA and vcRNA was noted in very low amounts starting at 5 dpi (Fig 5C). In leaves infiltrated with pcDNA1-mut construct, vRNA and vcRNA were not detected (Fig 5C). Comparative analysis of the positive (vRNA) and negative (vcRNA) strand levels of CiLV-C RNA between pcDNA1-WT and pcDNA1-p29fs showed a 142-fold and 40.7-fold decrease in CiLV-C vRNA and vcRNA levels at 6 dpi, when p29 expression was impaired. It is important to note that the membrane exposure time was increased 20-fold compared to previous assays to enable the visualization of vcRNA in the samples containing p29fs. From this result, we can conclude that the expression of p29 is not crucial but is required for efficient viral accumulation. Infectious clones defective in RNA silencing suppressor (RSS) proteins exhibit vRNA accumulation comparable to those of the WT at the early stage of infection (5 dpi), which progressively declines over the course of the infection [34,35]. However, the results from Fig 5C do not show similar levels of vRNA and vcRNA between the WT and the p29 defective vectors at the early stage of infection, suggesting that the mechanism by which p29 enhances RNA replication may not be directly linked to its RSS activity [6]. We also assessed whether vRNA accumulation in leaves infiltrated with the construct lacking p29 decreases over the course of the infection, which would indicate that its reduced replication rate is associated with the absence of the VSR. At 3 to 18 dpi, leaves expressing the p29 frameshift deletion construct show a progressive increase in both positive and negative strand CiLV-C RNA, with a progression profile similar to that observed in the WT (Fig 5D), suggesting that the diminished replication rate is not attributable to the absence of the VSR. This finding further reinforces that p29 RSS activity is not the main factor related to efficient levels of viral replication. To gain additional insights into this aspect, we performed complementation assays by *trans* expression of well-characterized viral suppressors of RNA silencing (VSRs) into binary vectors with the rCiLV-C-GFP-p29fs (pcDNA1-GFP-p29fs + pcDNA2). For this assay, we selected VSRs that interfere with different steps of the RNA silencing machinery: the helper-component proteinase (HCPro) from tobacco etch virus (TEV), the p19 from tomato bushy stunt virus (TBSV), the 2b protein from cucumber mosaic virus (CMV), the p25 from potato virus X (PVX), and the coat protein from turnip crinkle virus (TCV). Notably, the TCV CP not only acts as a VSR but also likely plays a role in replication, given its association with a transcription factor [36]. At 6 dpi, leaves expressing rCiLV-C-GFP-p29fs with HCPro, p19, p25, or 2b proteins did not show GFP fluorescence, in contrast to *trans*-expressed p29, which resulted in a strong GFP signal at 3 dpi (Fig 5E). Notably, *trans* expression of TCV CP successfully rescued the replication of the defective CiLV-C p29 clone at high levels, with the GFP fluorescence being less intense at 3 dpi compared to the p29 expression. However, at 6 dpi, the GFP intensity in leaves infiltrated with TCV CP was slightly higher than in those p29 (Fig 5E). Northern and western blot analysis at 7 dpi confirmed the visual results presented in Fig 5E. No detectable level of vRNA, vcRNA, or GFP expression were observed in the samples expressing HCPro, TBSV p19, PVX p25, and CMV 2b, similar to the negative control (Leader peptidase protein, Lep). In contrast, vRNA, vcRNA, and GFP expression were detected in samples expressing p29 and TCV CP *in trans* (Fig 5F). To verify the correct expression and functionality of the VSRs, we co-infiltrated these proteins with a GFP binary construct, and the GFP expression was monitored at 7 dpi. The transient expression of an RSS blocks the locally induced silencing triggered by the GFP, allowing sustained visualization of the bright green signal for more than 6 days. As expected, all VSRs maintained GFP expression, in contrast to Lep (negative control) (Fig 5G). Additionally, we investigated whether CiLV-C could effectively replicate when the p29 gene was replaced with the TCV CP gene. No increase in vRNA1 levels was detected, and no subgenomic RNAs derived from RNA2 were observed (S8 Fig), suggesting that the p29 coding sequence may contain intrinsic cis-acting elements essential for the formation of the viral replication complex.

### Cilevirus capsid protein acts as an RNA-binding protein and interacts with eukaryotic initiation factor 4A (eIF4A)

The inability of VSRs, like HCPro and p19 to rescue, at high levels, the replication of the CiLV-C p29 defective clone, opens the possibility that p29 might enhance CiLV-C RNA accumulation by interacting with host factors involved in gene regulation pathways. To generate hypotheses about mechanisms underlying p29 activity, we performed an immunoprecipitation (IP) assay followed by mass spectrometry sequencing. The strategy to identify host factors interacting with p29 during the viral infection cycle involved incorporating an immuno-epitope into the p29 coding sequence, ensuring that the viral cycle remained unaffected. For this end, we fused the HA-Tag to the N- or C-terminus of the p29 by genomic modification of pcDNA1-WT plasmid, resulting in pcDNA1-HA:p29 and pcDNA1-p29:HA constructs. First, we tested the infection efficiency of the recombinant clones. *N. benthamiana* leaves were infiltrated with *Agrobacterium* cultures carrying pcDNA1-HA:p29 or pcDNA1-p29:HA mixed with culture carrying the pcDNA2-Δp24-DsRed, which enables the virus spread visualization via RFP in place of the p24 gene without affecting viral local movement. Then, the cell-to-cell movement was monitored by DsRed fluorescence in comparison with WT (pcDNA1-WT). The presence of HA-Tag at the N-terminus of p29 abolished viral cell-to-cell movement. Only individual DsRed fluorescent cells were visualized at 12 dpi (S9A Fig). However, the construct with HA-tagged at the C-terminus of p29 resulted in infectious foci similar to the WT control (S9A Fig). Next, we tested the mechanical transmission efficiency of HA-targeted clones. Leaves mechanically inoculated with extract of tissue infiltrated with pcDNA1-p29:HA generated infectious foci comparable to the control (WT) (1^st^ passage, S9A Fig). No DsRed signal was detected in leaves mechanically inoculated with pcDNA1-HA:p29. (S9A Fig). Regarding vRNA accumulation, the presence of HA-tagged at the C-terminal of p29 reduced vRNA accumulation (S9B Fig), but without negatively affecting pathogenicity, as demonstrated by the foci formation assays and viral transmission to new plants. The relationship between the foci formation, efficient mechanical transmission, and high vRNA accumulation indicates that CiLV-C-p29:HA is an optimal candidate for the IP experiments aimed at identifying potential host interactors involved in CiLV-C replication.

The IP assay was conducted using the HA epitope and the Pierce Magnetic HA-Tag kit. pCiLV-C-WT and pCiLV-C-HA were infiltrated in *N. benthamiana* leaves, and at 12 dpi, plant tissues were collected for protein extraction and subsequent IP. The untagged version was employed as a control. Western blot analysis demonstrated the presence of CiLV-C p29-HA in both the crude protein extract and the finished immunoprecipitate obtained from tissue infiltrated with the pCiLV-C-HA, but not in those obtained from the tissue inoculated with the untagged version (pCiLV-C-WT) (S9C Fig). Next, the immunoprecipitated proteins were subjected to mass spectrometry sequencing. The Excel formula = FILTER(X2:X450;COUNTIF(Y2:Y300;X2:X450)=0) was utilized to filter potential host interactors by identifying those unique to CiLV-C HA IP sample in comparison to the mock sample (healthy plant) (S1 Table), which resulted in the identification of 177 potential interactors (S1 Table). Functional analysis using Omicsbox software grouped these interactors within three ontological categories: biological process (BP), molecular function (MF), and cellular component (CC). 35.7 percent of the proteins were associated with BP, 36.8% with MF, and 27.3% with CC (S10A Fig). Presuming that p29 might enhance CiLV-C RNA accumulation by interacting with host factors associated with transduction, we focused specifically on translation initiation factors classified within the MF ontology category (S10B Fig). Among them, we highlight the eukaryotic initiation factor 4A, which has a high probability of interacting with p29 indicated by the number of reads obtained (S1 Table).

**Fig 6:**
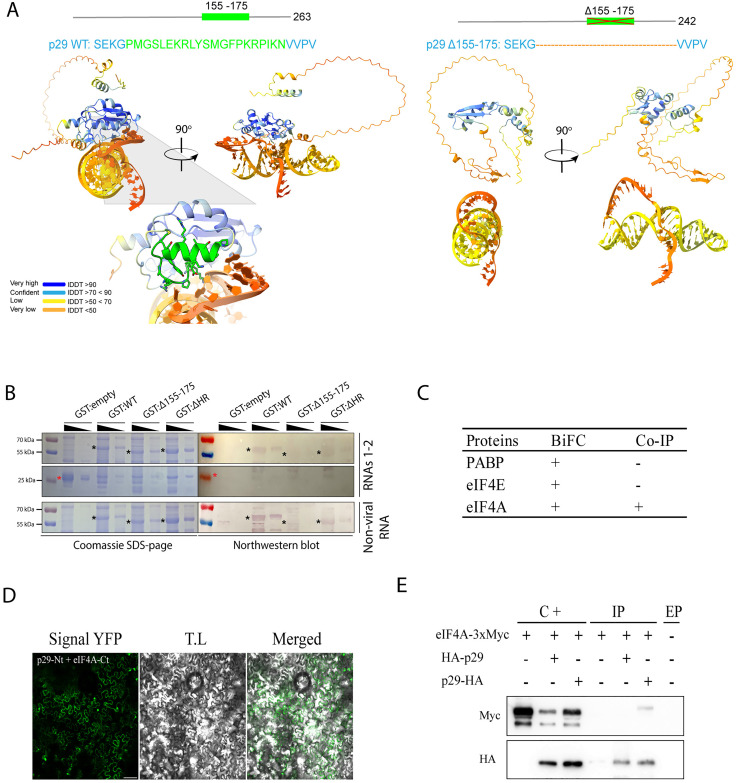
CiLV-C p29 acts as an RNA-binding protein and interacts with eukaryotic initiation factor 4A (eIF4A). (A) Prediction of p29-RNA interaction using AlphaFold 3. The model predicts the interaction of p29 and 3’UTR of CiLV-C RNA2 (left image) versus a model generated by deleting the 155-175 residues of p29 (right image). Schematic representation of p29 ORF highlights the putative domain of RNA interaction (green box). Magnification in the left image highlights the alpha helix formed by the residues 155-175 and its potential involvement in RNA interaction. lDDT corresponds to a per-atom confidence estimate on a 0-100 scale where higher values indicate greater confidence. (B) Analysis of the RNA-binding activity of GST:p29 proteins by northwestern blot assay. The GST fusion protein was produced in a prokaryotic system. Duplicated membranes containing GST:empty (lines 1 and 2), GST:p29WT (lines 3 and 4), GST:p29Δ155-175 (lines 5 and 6), and GST:p29ΔHR (lines 7 and 8) were resolved by 12% SDS-PAGE, transferred to a PVDF membrane, and incubated with 1 µg of CiLV-C RNAs 1, 2 or non-viral RNA labeled with digoxigenin. Samples were loaded at different concentrations (1:4 dilution factor), starting from 5μL and decreasing to 1.25 μL. The left panel shows a Coomassie blue-stained gel, while the right panel shows results from northwestern blot analysis. Full-length GST:p29 and its deletion variants are indicated by black asterisks. The positions of molecular mass markers (in kDa) are shown on the left side. (C) Summary of interactions analysis between CiLV-C p29 and eukaryotic initiation factors eIF4E, eIF4A, and the Poly(A)-binding protein (PABP), using two molecular techniques: Bimolecular fluorescence complementation (BiFC) and Co-immunoprecipitation (CoIP) assays. (D) *In vivo* interaction between p29 and eIF4A. eIF4A was targeted at its C-terminus with NYFP and CYFP and co-expressed with p29 fused at its N- or C-terminus with the NYFP or CYFP. A representative protein pair combination is indicated at the top of the image. The image shows the reconstitution of the YFP fluorescence distributed through the cell cytoplasm of *N. benthamiana* cells. The image is representative of several infiltrated leaves from three different plants. The fluorescent signals were captured at 3 days post-infiltration (dpi). The green (GFP), transmitted light (T.L) channels, and merged images are shown in the figure. Negative control corresponds to the expression of the eIF4A in combination with Ncyt constructs and p29 in combination with the Cer construct (S11 Fig). The bar indicates 50 μm. (E) CoIP of CiLV-C p29 and eIF4A. Extracts from *N. benthamiana* leaves expressing p29 fused at its N- or C-terminus with an HA epitope and 3xMyc-tagged eIF4A at its C-terminus were analyzed at 3 dpi. The Co-IP assay was addressed using the Pierce HA-Tag Magnetic IP/Co-IP Kit. Western blot analysis was conducted using Myc and HA antibodies. C + , positive control (non-immunoprecipitated samples); IP, immunoprecipitated samples. The ‘+’ and ‘–‘ signs indicate the presence or absence of the corresponding proteins in the leaf extracts. EP, empty line corresponds to non-loaded line.

Before exploring the potential interaction between p29 and eIFs, we first aimed to evaluate whether p29 possesses RNA-binding property. It is known that viral RNA biding-proteins (RBPs) play important roles in multiple steps of the viral cycle, including the translation and replication of viral genomic RNAs [37]. First, we predicted the potential p29 and RNA interaction using AlphaFold. Models were generated by analysis of the interaction between 5’ and 3’ UTRs of CiLV-C RNAs 1 and 2 and p29. The highest templating modeling (pTM) score, which measures the accuracy of the entire structure [38], was observed for the interaction between p29 and the 5’UTR of RNA2 (pTM = 0.45). From this model (Fig 6A, left image), we identified the residues 155-PMGSLEKRLYSMGFPKRPIKN-175 with a potential role in RNA interaction. Deletion analysis prediction suggests that the absence of 155–175 residues disrupts the association between p29 and 5’UTR of RNA2 (Fig 6A, right image). To validate the prediction and assess the RNA-binding property of p29, GST-tagged p29WT, GST-p29Δ155–175, and GST-p29ΔHR were expressed in *Escherichia coli*. ΔHR corresponds to a deletion of the p29 hydrophobic region (176–194 residues), demonstrated as a possible interaction domain with cell membranes [4]. Based on this aspect, we reasoned that deletion of this region would potentially not affect the protein’s binding to RNA and was therefore used as a control. Northwestern blot assay revealed that p29WT interacts with CiLV-C RNAs 1 and 2, indicated by a positive band signal detected across different protein concentrations (Fig 6B). The capacity of RNA union was maintained when the HR domain was deleted. However, when the residues 155–175 predicted as a domain of RNA interaction were deleted, we did not notice a clear band signal at the corresponding size indicated on the mirror gel (Fig 6B, asterisks). No signal was observed in the fraction loaded with empty GST, thereby excluding the possibility that the positive signal came from an unspecific GST-RNA union (Fig 6B, gel below). Positive signal bands below the expected size of GST:p29 (56 kDa) likely correspond to GST-p29 degradation products. Furthermore, we assessed whether p29-RNA binding is specific to vRNA. To this end, we conducted a northwestern blot analysis using a non-viral RNA transcript (~ 300 bp plasmid-derived). A positive band signal was detected for GST:p29WT and GST:p29ΔHR but not for GST-p29Δ155–175 (Fig 6B), further confirming the relevance of this region for RNA union and indicating that p29 RNA binding activity is not restricted to vRNA. These results reveal that the CiLV-C capsid protein possesses RNA-binding activity.

Subsequently, we employed different approaches to validate that p29 binds to eIF4A. Furthermore, we also investigate potential interactions between p29 and other components of the eIF4F complex. Bimolecular fluorescence complementation (BiFC) analysis showed the reconstitution of the YFP fluorescent signal distributed through the cytoplasm of *N. benthamiana* leaves infiltrated with *Agrobacterium* cultures carrying constructs of p29 targeted with N-or C-terminal fragments of YFP and eIF4E, eIF4A, and PABP fused with the counterpart at either the N- or C-terminus (Figs 6C and 6D, and S11). Considering that it is crucial to analyze the same protein couple using at least two different protein-protein interaction (PPI) approaches [39], a co-immunoprecipitation (CoIP) assay was performed to confirm the *in vivo* interactions. *N. benthamiana* plants were agroinfiltrated with bacterial cultures carrying the p29-HA or HA-p29 and 3xMyc targeted to the N-or C-terminus of the eIF4A, eIF4E, and PABP, and at 3 dpi, subjected to the CoIP. Among all tested combinations and couple proteins, a clear band was observed in the immunoprecipitated extract of the combination between p29-HA with eIF4A-3xMyc (Fig 6E), confirming the mass spectrometry sequencing results, which indicated p29-eIF4A interaction. No bands were detected in the immunoprecipitated extract, indicating an interaction between p29 with PABP and eIF4E proteins (S11 Fig).

### eIF4A acts as a pro-viral factor for CiLV-C infectivity in *N. benthamiana* plants

**Fig 7:**
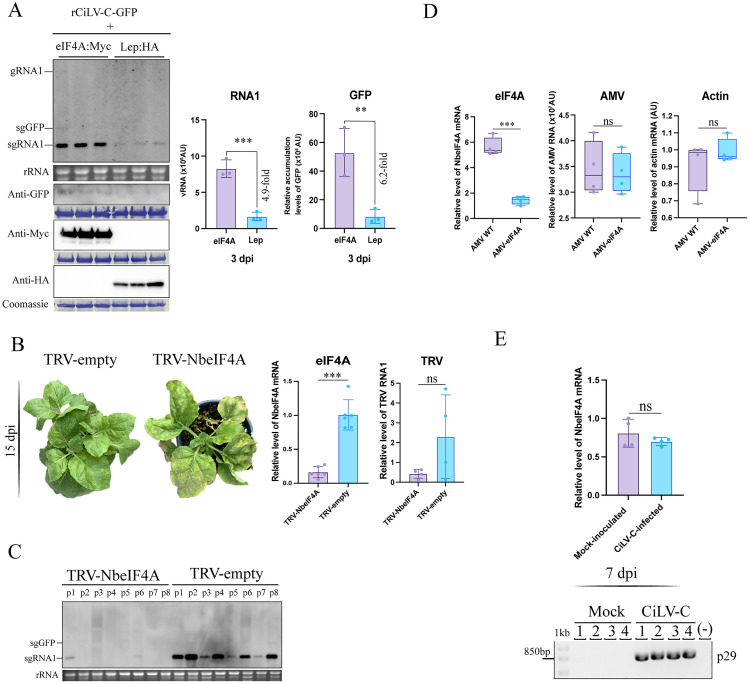
Effect of *N. benthamiana* eIF4A overexpression and knockdown on rCiLV-C-GFP infection. (A) Left panel shows northern and western blot analyses (3 dpi) of levels of CiLV-C genomic (g) and subgenomic (sg) RNAs and GFP protein in plants overexpressing eIF4A-3xMyc and Lep-HA (control) infected with rCiLV-C-GFP. RNA detection was performed using a DIG-probe complementary to the CiLV-C p29. GFP, HA and Myc detection was carried out using specific antibodies against these proteins. The position of CiLV-C gRNA1, sgGFP, and sgRNA1 are indicated in the northern blot. Ethidium bromide-stained rRNA serves as a loading control. Coomassie blue-stained proteins serve as a loading control for the western blot. The graphs represent the mean relative accumulation levels of CiLV-C RNA1 and GFP protein from plants expressing eIF4A-3xMyc or Lep-HA. Error bars represent SD. Asterisks indicate statistically significant difference according to unpaired Student’s t-test (two-tailed), *** **p* *< 0.001, ** *p* < 0.01. (B) Phenotype of eIF4A-silenced *N. benthamiana* via TRV-induced gene silencing at 15 dpi. The graph represents eIF4A mRNA transcript levels (left) and TRV vRNA (right) in *N. benthamiana* plants infected with TRV-empty and TRV-*Nbe*IF4A. The *L23* housekeeping gene was used as an internal control. Bars indicate standard error obtained from 6 or 4 plants per treatment. (C) Northern blot analysis of CiLV-C RNA1 accumulation in control (TRV-empty) and eIF4A-silenced (TRV-*Nb*eIF4A) *N. benthamiana* plants infected with rCiLV-C-GFP at 7 days post-mechanical inoculation. RNA detection was performed using a DIG-probe complementary to the CiLV-C p29. The localization of CiLV-C sgRNA1 and sgGFP is indicated in the northern blot. Ethidium bromide-stained rRNA serves as a loading control. (D) The left graph represents eIF4A mRNA transcript levels in *N. benthamiana* plants infected with AMV-empty and AMV-*Nbe*IF4A. The *L23* housekeeping gene was used as an internal control. The middle graph represents the relative accumulation of AMV RNA (arbitrary units, AU) determined by northern blot analysis using a specific AMV riboprobe from *N. benthamiana* plants infected with AMV-empty and AMV-*Nb*eIF4A . The right graph represents the relative levels of Actin mRNA in *N. benthamiana* plants infected with AMV-empty and AMV-*Nbe*IF4A. Bars indicate standard error obtained from four plants per treatment. (E) The graph shows eIF4A mRNA transcript levels in *N. benthamiana* plants either infected with CiLV-C or mock-inoculated at 7 dpi. Bars represent the standard error from four biological replicates per treatment. RT-PCR using primers for the *p29* gene was performed to confirm the infection status and plant health.

In order to verify the functional role of eIF4A in CiLV-C infection/accumulation, we first assessed the eIF4A overexpression by *Agrobacterium*-mediated transformation in *N. benthamiana*. The functional role of eIF4A in CiLV-C infection was validated by CiLV-C RNA/protein accumulation in *N. benthamiana* leaves agroinfiltrated with rCiLV-C-GFP plus eIF4A-3xMyc or Lep-HA (control) at 3 dpi. CiLV-C RNA1 accumulation was 4.29-fold greater with overexpression of eIF4A-3xMyc than in control plants (overexpression of Lep-HA), with a significant effect on CiLV-C protein accumulation (6.2-fold greater than Lep) demonstrated by detection of GFP expression from the rCiLV-C-GFP infection (Fig 7A).

To confirm the positive role of eIF4A in CiLV-C infection, the expression of eIF4A was knocked down by VIGS in *N. benthamiana*. A 300-bp fragment from the *NbeIF4A* gene was used to generate tobacco rattle virus - TRV2-NbeIF4A, which was agroinfiltrated with TRV1 in *N. benthamiana*. At 10 days post-TRV agroinfiltration, the plants were mechanically inoculated with an extract of *N. benthamiana* leaves infected with rCiLV-C-GFP. RT-qPCR analysis revealed a reduction in NbeIF4A transcript levels of approximately 84% in TRV-NbeIF4A plants relative to the control plants (TRV-empty) at 17 days post-TRV inoculation (Fig 7B). Typical TRV symptoms were visualized in control plants (TRV-empty) at 15 dpi, whereas eIF4A-silenced plants showed morphological changes (leaf necrosis, chlorosis, and leaf-twisting) (Fig 7B).

At 7 days post-CiLV-C mechanical inoculation, CiLV-C RNA1 accumulation was monitored by northern blot analysis. A clear decrease in CiLV-C RNA1 accumulation was noticed in the eIF4A-silenced plants when compared to control plants (Fig 7C). To exclude the possibility that the observed reduction in CiLV-C accumulation was a consequence of a confounding effect associated with the *eIF4* silencing phenotype, we examined the accumulation of vRNA using both TRV- and AMV-induced gene silencing systems [40]. We selected the AMV system because it enables a more precise and targeted gene silencing mechanism compared to the TRV-based approach. The AMV construct includes only 54 nucleotides of the target sequence, minimizing the likelihood of off-target effects. In contrast, TRV vectors typically use sequences longer than 300 nucleotides, increasing the risk of non-specific silencing. We reasoned that, if the eIF4A silencing phenotype had a major confounding impact on viral accumulation, a similar reduction should be observed in the virus used to induce gene silencing. For TRV, we observed non-significant differences in TRV RNA1 accumulation between TRV-eIF4A and TRV-empty plants (Fig 7B, right graph). In the AMV system, we first confirmed a 74.42% reduction in *NbeIF4A* transcript levels in AMV-eIF4A plants compared to the control (AMV-empty) (Fig 7D, left graph). Next, we evaluated AMV RNA accumulation and found no significant differences between AMV-eIF4A and AMV-empty plants (Fig 7D, middle graph). To further validate our results, we evaluated the expression of a structural plant gene (Actin) and found no significant differences in transcript levels between treatments (Fig 7D, right graph). Together, these results indicate that the reduction in CiLV-C accumulation is likely due to a virus-specific requirement for eIF4A rather than a general effect of gene silencing.

We also investigated whether CiLV-C infection affects *eIF4A* expression. qPCR analysis revealed no significant differences in *NbeIF4A* mRNA levels between mock-inoculated and CiLV-C-infected plants (Fig 7E), indicating that eIF4A expression is not altered by CiLV-C infection. The lack of changes in eIF4A transcript levels during CiLV-C infection suggests that the virus relies on equilibrated eIF4A expression, possibly to avoid triggering host defenses; however, we cannot exclude the possibility that regulation occurs at a post-transcriptional level.

To further investigate the requirement of eIF4A for successful CiLV-C infection, we tested whether eIF4A overexpression could rescue the accumulation of the p29fs mutant. Leaves were co-infiltrated with pcDNA1-GFP-p29fs and pMOG-eIF4A. As a positive control, leaves were co-infiltrated with pcDNA1-GFP-p29fs and pMOG-p29, while leaves infiltrated with pcDNA1-GFP-p29fs and pMOG-Lep served as a negative control. At 5 dpi, no visible GFP expression was detected in the combination of pcDNA1-GFP-p29fs with pMOG-eIF4A, nor in the negative control. In contrast, the positive control exhibited a strong GFP signal (S12 Fig). As expected, eIF4A overexpression did not rescue the replication of the p29-defective clone, likely due to the requirement for a direct interaction between p29 and eIF4A to promote viral accumulation.

### The subcellular expression of p29 and eIF4A reveals a dynamic interaction during the viral cycle

**Fig 8:**
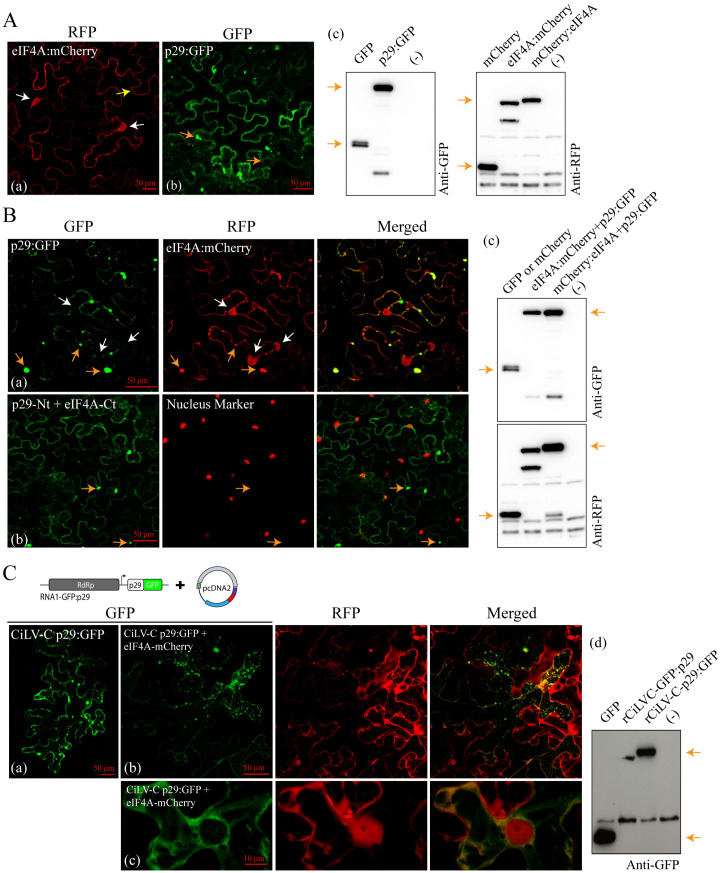
Structure formation and intracellular distribution of p29 and eIF4A after transient expression and during CiLV-C infection. (A) Subcellular localization analysis following transient expression of p29 (a) and eIF4A (b), fused at their C-termini to GFP and mCherry, respectively in *N. benthamiana* leaves. White, yellow, and orange arrows indicate the nucleus, cell wall, and p29 punctate bodies, respectively. (B) Co-expression of p29:GFP and eIF4A:mCherry (a), and subcellular localization of the p29–eIF4A complex co-expressed with the nuclear marker SV40-RFP (b). p29 and eIF4A were fused to the N-terminal (Nt-YFP) and C-terminal (Ct-YFP) fragments of YFP at their C-termini, respectively. White arrows indicate eIF4A:mCherry signal in the nucleus, and orange arrows highlight punctate cytoplasmic bodies showing co-localization of p29:GFP and eIF4A:mCherry in (a). In panels A-a, B-a, and C-c, the identification of nuclei was based on the presence of the nucleolus, which is clearly discernible in fluorescent-stained nuclei. (C) Subcellular localization of p29 and eIF4A during CiLV-C infection. (a) Intracellular distribution of p29:GFP expressed from rCiLV-C construct carrying GFP fused to the C-terminus of p29. A schematic representation of the rCiLV-C-p29:GFP genome is shown at the top of the panel. (b) Intracellular distribution of p29:GFP and eIF4A:mCherry in leaves infected with rCiLV-C-p29:GFP. (c) Higher magnification image showing the nucleus in leaves infected with CiLV-C expressing p29:GFP and eIF4A:mCherry. Fluorescent signals were captured at 72 h post-infiltration for transient expression and at 5 days post-infection for CiLV-C-infected leaves using a confocal microscope Zeiss LSM 780 AxiObserver. Green (GFP), red (RFP), and merged fluorescent channels are shown. Bars correspond to 10-50 μm. Western blot analysis of transient accumulation in *N. benthamiana* of GFP (~27 kDa), p29:eGFP (~56 kDa), mCherry (~27 kDa), eIF4A:mCherry (~80 kDa), and mCherry:eIF4A (~80 kDa) are presented in panels A-c and B-c. Panel C-d shows p29:GFP expression during viral infection. Monoclonal antibodies against GFP and RFP were used to detect both free and fused forms of GFP and mCherry, indicated by orange arrows.

To gain further insight into the functional association between p29 and eIF4A, we assessed their subcellular localization after transient expression and during CiLV-C infection. First, we evaluated the individual transient expression of p29 and eIF4A. The p29 was previously observed to have a cytoplasmic localization, forming numerous inclusion bodies dispersed throughout the cell, as well as in the nucleus, where its presence correlates with a reduction in nucleus size [4,41]. In this study, we confirmed its cytoplasmic localization, characterized by the formation of several inclusion bodies (orange arrows in Fig 8A, panel b). Leaves expressing eIF4A:mCherry showed a diffuse signal evenly distributed in the cytoplasm (yellow arrow in Fig 8A, panel a) and within the nucleus (white arrow in Fig 8A, panel a). No differences in localization were observed when mCherry was fused to either the N- or C-terminus of eIF4A; therefore, we present here the results obtained with the C-terminal fusion. To confirm proper protein expression, western blot analysis was performed using monoclonal antibodies against GFP and RFP to detect both free and fused forms of GFP and mCherry (Fig 8A, panel c and Fig 8B, panel c).

Next, we examined the subcellular localization resulting from the co-expression of p29:GFP and eIF4A:mCherry. The p29:GFP signal exhibited the same distribution pattern observed during its individual expression. In contrast, the eIF4A:mCherry signal displayed a marked difference, with the formation of inclusion bodies that co-localized with p29:GFP aggregates (Fig 8B, panel a, orange arrows). Co-localization was observed both in inclusion bodies and as a diffuse signal throughout the cytoplasm. The nuclear localization of eIF4A was preserved, whereas no GFP signal was detected in the nuclei. To further confirm the subcellular distribution of the p29–eIF4A complex, we co-expressed these proteins using BiFC constructs along with a nuclear marker. Inclusion bodies resulting from the interaction between p29 and eIF4A were observed exclusively in the cytoplasm (Fig 8B, panel b), resembling those seen in Fig 8B, panel a. No co-localization with the nucleus was detected, indicating that the interaction between p29 and eIF4A occurs specifically in the cytoplasm.

Finally, we assessed the subcellular localization of the p29–eIF4A complex in the context of viral infection. To this purpose, the constructs pcDNA1-p29-GFP and pcDNA1-GFP-p29 were adapted by removing the GJ sequence, resulting in GFP fused to either the N- or C-terminus of p29. Protein fusions were confirmed by western blot analysis (Fig 8C, panel d). Both constructs produced similar fluorescence patterns; therefore, we present the results obtained with the pcDNA1-p29:GFP construct. At 5 dpi, the rCiLV-C-p29:GFP (pcDNA1-p29:GFP + pcDNA2) expression showed a GFP signal distributed in inclusion bodies throughout the cytoplasm (Fig 8C, panel a), similar to the pattern observed during ectopic p29:GFP expression. Co-infiltration of rCiLV-C-p29:GFP and eIF4A:mCherry in *N. benthamiana* leaves resulted in co-localization of both signals, observed as a diffuse cytoplasmic fluorescence and within granular cytoplasmic structures (Fig 8C, panel b). As observed in Fig 8B, GFP signal was not detected in the cell nucleus co-localizing with eIF4A (Fig 8C, panel c). Fluorescence was monitored from 2 to 7 dpi, and no differences in complex distribution were observed during this period.

The p29 RBP activity, p29 capability of interacting exclusively with initiation factor (eIF4A), the subcellular distribution of the p29-eIF4A complex, and the impact of eIF4A on CiLV-C infection – enhanced by its overexpression and impaired by its knockdown - collectively suggest that CiLV-C capsid protein may recruit eIF4A to promote efficient viral replication.

### TCV CP interacts with eIF4A, and knockdown of eIF4A negatively impacts TCV replication

In this study, TCV CP was able to rescue the RNA accumulation at high levels of a CP-defective CiLV-C virus (Fig 5F). To explore a potential relationship between the initiation factor eIF4A and the ability of TCV CP to rescue CiLV-C infectivity, we examined whether TCV CP shares the capacity of CiLV-C CP to interact with eIF4A and evaluated the impact of eIF4A on TCV replication. BiFC and CoIP assays revealed the ability of TCV CP to interact with eIF4A (S13 A and S13B Fig). Additionally, both overexpression and knockdown of eIF4A negatively affected TCV accumulation (S13 C and S13D Fig). These findings suggest that viral CPs can increase the vRNA accumulation by manipulating host initiation factors.

## Discussion

We have engineered a recombinant CiLV-C virus able to express the GFP reporter gene stably even after serial mechanical passages. We have used this recombinant cilevirus as a valuable proof-of-concept for advancing the molecular understanding of kitaviruses’ biology. This approach allows us to dissect the viral genomic components and elucidate their roles in infection mechanisms and pathogenesis. By manipulating specific viral genes, we explored the functional contribution of individual proteins to the virus life cycle, thereby providing critical insights into the molecular determinants of cilevirus movement, replication, morphogenesis, and virulence.

The 30K MP of cileviruses is a tubule-forming protein that interacts with its cognate p29 protein, associates with the ER network and plasmodesmata, and integrally binds cell membranes. In addition, it is capable of nucleolar targeting and interacts with fibrillarin [7]. Studies based mainly on positive-strand RNA plant viruses have proposed two main mechanisms for intercellular viral movement. These mechanisms differ in whether the MP interacts with intact viral particles or with viral ribonucleoprotein (vRNP) complexes, each generating specific modifications to the plasmodesmata channels [42–44]. In the case of cilevirus, the local movement mechanism has been previously explored through studies of the MP functionality using heterologous viral systems, which allowed the proposal of two possible routes for cell-to-cell transport [7]. Since virus particles are not required for the local transport mediated by cilevirus MPs in the AMV infectious context, it was proposed that the cilevirus MPs assist the transport of vRNP complexes rather than virion particles [7]. Successful local movement was observed in the pCiLV-C construct with a deletion of the p24 gene (Fig 3), which is involved in the structural assembly of virion particles (Fig 4E). This finding confirms that the presence of a mature virion is not essential for the cell-to-cell movement of cileviruses, aligning with our previously reported model. The failure of the rCiLV-GFP-MPfs (MP translational defect) and rCiLV-C-GFP-ΔMP/DsRed (MP gene deletion) mutants to move from cell-to-cell provides conclusive and direct evidence that p32 is the CiLV-C movement protein. In addition, the maintenance of local movement, despite the absence of p15, p61, and p24 expression, suggests that p32 is likely the key viral factor involved in local movement. Nevertheless, the involvement of p29 could not be confirmed, as its deletion significantly compromises CiLV-C infectivity. Future studies may provide deeper insights into the specific role of p29 in regulating or facilitating the movement process.

Unlike the results obtained with the frameshift mutants, the complete deletion of p15 and p61 genes abolished the capacity of the virus to move, indicating that strong cis-elements are located in these regions. In this regard, when the p15 gene was replaced by DsRed, the accumulation of RNA 2 decreased (compare Δp15 with Δp61, Fig 3E), which may explain the inability to move localy, likely due to low production of MP. Moreover, the low RNA2 production suggests that the missing sequence may contain a key component necessary for RNA2 synthesis. Regarding p61, a drastic reduction in RNA2 was not observed, but the viral movement was abolished. We speculate that the MP promoter sequence may be located in the p61 sequence. Further research is needed to better understand this issue.

Abundant reactive oxygen species (ROS) production with the presence of cell death, leading to a hypersensitive-like response, are found in CiLV-C-infected lesions [12]. This host response is believed to be the key factor in restricting the viral systemic spread of these atypical kitaviruses. However, the avirulence (*Arv*) effector responsible for triggering the host cell death pathway remains unidentified. None of the CiLV-C frameshift mutants impaired the necrotic lesion response or restored the systemic spread of the virus (non-positive signal in tissue print analysis of leaf petiole), indicating that p15, MP, p24, and particularly p61 – whose ectopic expression active defense signal transduction leading to HR response [45] - appear not to be directly implicated as *Avr* effectors on CiLV-C infection context. To further explore this issue, during our analysis with CiLV-C RNA1-GFP (Fig 1), we investigated whether the necrotic response occurs independently of the proteins encoded by RNA2. We assessed the presence of necrosis in leaves expressing only viral component 1 and observed that necrosis persisted (S14 Fig). However, the intensity of this response was reduced, likely due to the restriction of local movement, which limits the efficient spread of the infection. Moreover, we hypothesized that the lack of systemic movement observed in infections with CiLV-C RNA1 carrying the MP (pcDNA1-CiLV-C MP) (S3 Fig) could also result from the HR triggered by the RNA1 expression. Considering these findings and the fact that this HR phenotype is conserved across family members, we speculate that the *Avr* determinant/effector associated with the HR response may be related to the viral polymerase, as it contains the most conserved domains among the members of the *Kitaviridae* family. Importantly, we cannot exclude the possible relevance of p29 in the process, as its deletion leads to the collapse of the entire viral system, preventing us from assessing its implications. In any case, our mass spectrometry sequencing results indicate that p29 may interact with host factors that activate hypersensitive-induced response and apoptosis (see S1 Table, hypersensitive-induced response protein 1-like and apoptosis-inducing factor), opening the possibility that p29 may play a key role in the activation of the plant's antiviral defense mechanism.

In most cases, enveloped virions are covered with a coat composed of glycoproteins essential for viral pathogenesis and/or morphogenesis [46]. *Kitaviridae* is the sole plant virus family in the class *Alsuviricetes* comprising enveloped virions [1]. Remarkably, the p61 protein has all the features of a virion glycoprotein: it contains a signal peptide at its N-terminal (cleavage site), followed by four N-glycosylation sites along the protein, and three transmembrane domains (TMDs) in its extreme C-terminal [4]. Despite these features, the deletion of p61 did not affect the formation of mature enveloped virions (Fig 4E), even though this protein possesses TMDs, indicating its potential presence within the viral envelope. This observation suggests that p61 may not participate in the budding process (assuming the virus is able to replicate in its vector), which is crucial for the release of animal-infecting viruses, where surface glycoproteins play critical roles during cellular entry [47,48]. In this context, we speculate that p61 may act as a non-structural glycoprotein that is not integrated into the virion but rather secreted into its mite host cells, similar to some animal viruses, such as the dengue virus [49]. Regardless, the precise role of p61 as a glycoprotein requires further investigation.

Some enveloped viruses possess a structural matrix protein located beneath the viral envelope. This protein plays a crucial role in virion assembly and budding, interacts with ribonucleoproteins (RNPs) and the viral membrane, and contributes to the structural stability of virus particles [50,51]. Cileviruses' p24 protein belongs to the SP24 family of virion membrane protein of plant and insect viruses [9]. Herein, using a p24-defective virus, aggregates of dense granular material were extensively observed in place of the typical compacted electron-dense structures. Similar virus-induced inclusions, representing dense nucleocapsid aggregates, have been reported in plants infected with a morphologically defective tomato spotted wilt virus (TSWV) isolate lacking the capacity to produce the membrane glycoproteins (Gp) [52]. More recently, TEM analysis of an engineered TSWV carrying the CRISPR-Cas12a gene in place of the Gp gene, revealed the formation of scattered, electron-dense, amorphous nucleocapsid aggregates (NAs) in the cytoplasm, which failed to undergo morphogenesis [53]. These structures closely resemble those observed in CiLV-C p24fs-infected tissue. In this sense, the p24, like the Gp of orthotospovirus, appears to be indispensable for virus budding and envelopment [54]. The granular aggregates observed in CiLV-C-infected tissue may indeed represent non-enveloped virus-like particles (VLPs), or RNPs that accumulated in the cytoplasm due to their inability to bud into the ER, as a consequence of the absence of the p24 protein. In this sense, the sole expression of p29 has been shown to form stable VLPs [5], further supporting the notion that these aggregates may represent assembled capsid structures lacking envelope acquisition mediated by a matrix protein. We provide the first direct evidence that p24 is required for complete morphogenesis and assembly of CiLV-C enveloped particles. This finding is consistent with our previous characterization of p24 membrane topology and subcellular localization with ER membrane remodeling [4]. Collectively, these data strongly support the hypothesis that p24 plays a structural role, functioning as the viral matrix protein. Although current evidence suggests this model, we cannot rule out the possibility that CiLV-C particle formation requires two capsid components, with p24 potentially acting as one of them, contributing structurally to the capsid itself.

Symptom attenuation was most evident in the tissue infected with viruses defective in p29, p15, and MP. Our results suggest that the absence or gradual reduction in the severity of necrotic symptoms in leaves infected with p29- or MP-defect constructs is due to the decreased viral RNA content, caused by inefficient vRNA replication (p29 defect) and the absence of cell-to-cell movement (MP defect). However, in the case of the p15 and p15-p61 double mutation, no reduction in vRNA levels was observed in agroinfiltrated leaves, despite the attenuation of symptom severity. This indicates that these proteins may act as virulence determinants. In some plant viruses, the inactivation of VSR proteins has been associated with symptom attenuation [55]. However, there is not always a perfect correlation between suppression and virulence, as demonstrated by an analysis of amino acid substitutions in the RSS HCPro of TEV [56]. It is known that CiLV-C p15 and p61 possess RSS activity [6]. In this context, the obstruction of these protein activities through inhibition of their expression may have influenced symptom development by disrupting viral-host protein interactions related to RNA silencing pathways.

As commented above, the ectopic expression of p61 triggers a strong defense response in plants [45]. However, it was the only protein whose single deletion did not result in significant changes in viral RNA accumulation, particle formation, or symptom development. This observation suggests that p61 may not be essential in plant host infection. Instead, its main function could be related to vector specificity, acting as a key component required for transmission and/or playing a crucial role in the viral infection cycle within insect vector cells, similar to glycoproteins of several plant viruses [57,58]. Our next step is to investigate the role of p61 in vector-mediated transmission and its involvement in the viral infection cycle within animal cells.

**Fig 9:**
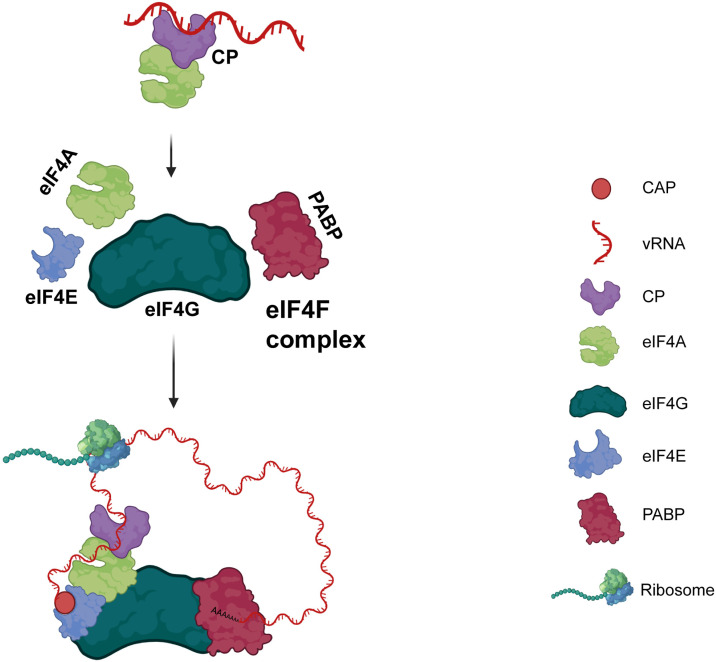
A model for the role of CP in viral infectivity. We propose that the CP possibly recruits eIF4A to preferentially promote the translation of vRNA by unwinding RNA structures at the 5’ untranslated regions (UTRs) of the viral genome, thereby facilitating access for translation initiation elements. This interaction may enhance viral protein synthesis, leading to increased accumulation of vRNA.

The regulation of viral protein synthesis in host cells is a crucial strategy that viruses employ to enhance their replication cycle while modulating host gene expression. Notably, the control of the translation initiation step, particularly through the eIF4F complex, is a mechanism commonly exploited by many animal and plant viruses. Three components of the eIF4F complex (eIF4A, eIF4E, and eIF4G) were reported to closely regulate the life cycle of various viruses, either promoting or restricting the infection [59,60]. In this study, we analyzed the interaction of p29 with two components of the eIF4F complex and PABP. A positive interaction was observed only with elF4A, confirmed by two different techniques, suggesting that p29 may play a role in the translation process. Our results indicate that eIF4A is a positive regulator of the CiLV-C cycle, likely through its interaction with p29, supporting the proposal of a model for the role of CP in viral infectivity based on its capacity to interact with both RNA and eIF4A. Our model posits that the CiLV-C CP possibly recruits eIF4A to preferentially promote the translation of vRNA by unwinding RNA structures at the 5’ UTRs and/or recruiting the translational complex, thereby facilitating access for translation machinery. This interaction may enhance viral protein synthesis, leading to increased accumulation of vRNA (Fig 9). It has been proposed that the eIF4A helicase activity could work by removing protein from the 5’UTR of the RNA-protein complex [61,62]. Although this model suggests a role for eIF4A in unwinding RNA structures at the CiLV-C 5’UTRs and/or recruiting the translational complex, further studies are needed to confirm this hypothesis. In this sense, the suggested model serves as a starting point to uncover the mechanism underlying CP activity in viral genomic replication.

Unexpectedly, TCV CP was the only VSR assayed able to functionally replace the CP activity of a cilevirus, leading to enhanced viral RNA accumulation. A previous study demonstrated that TCV CP prevents the nuclear localization of an initiation factor (TIP), suggesting that TIP might be a transcription factor involved in regulating the defense response of *Arabidopsis* to TCV [36]. As demonstrated for CiLV-C, TCV CP was able to interact with eIF4A, and alterations in eIF4A had a significant impact on TCV accumulation. We observed different behavior when comparing both viruses. While the silencing of the *eIF4A* gene negatively affected the accumulation of TCV, similar to what was observed for CiLV-C, overexpression also reduced TCV accumulation. These results point to an additional role of the TCV CP in virus translation. In this sense, some viruses redirect the translation machinery to their viral RNAs through interaction with eIF4A, either by inactivation or recruitment [62]. Further analysis will be conducted to determine the specific role of TCV CP in translation. We speculate that, beyond VSR activity, CPs of TCV and CiLV-C may play a key role in modulating translation initiation factors to favor the viral cycle. The ability of TCV CP to restore RNA accumulation of a virus belonging to a phylogenetically distinct family suggests the existence of a conserved, CP-dependent, likely replication-related mechanism that may be shared across distinct virus families. A recent study has revealed that TCV CP suppresses RNA decay by interacting with AtDcp1 and mediating its degradation through the 26S proteasome and autophagy pathways, effectively subduing antiviral RNA decay [63]. This mechanism could potentially be exploited by the TCV CP to enhance CiLV-C accumulation, representing a line of investigation for future studies.

## Materials and methods

### Plant materials

*N. benthamiana* plants were kept in a growth chamber with 16 hours (h) of light at 24°C and 8h of darkness at 18°C. All biological assays were performed with *N. benthamiana* plants at the four-leaf growth stage in three independent biological experiments (unless otherwise stated).

### *Agrobacterium* infiltration

For *Agrobacterium tumefaciens*-mediated transient transformation *in N. benthamiana,* plasmids were transformed into the C58 strain by the electroporation method. Individual agrobacterium cultures grew at 28 °C in Luria-Bertani (LB) medium containing rifampicin (100 mg mL^-1^) and kanamycin (50 mg mL^-1^) antibiotics for plasmid and agrobacterium selection. Agrobacterium cultures were resuspended in agroinfiltration buffer (10 mM MgCl_2_, 10 mM MES adjusted to pH 5.6) and OD_600_ adjusted to 0.1-0.5. For assays using the CiLV-C infectious clone, before infiltration, individual *Agrobacterium* suspensions (OD_600_ = 0.5) carrying each genomic component of the full-length CiLV-C clone (e.g., pcDNA1 or pcDNA2) were mixed in equal volume (1:1 ratio). To increase the stability of the viral genome transcripts, an agrobacterium culture carrying an expression cassette for the tobacco etch virus HCPro, was added to the mix at OD_600_ = 0.1. For experiments involving BiFC, which require the simultaneous expression of two different proteins, bacterial cultures were adjusted to an OD_600_ value of 0.4 and then mixed in equal proportions before infiltration. For overexpression analysis, the OD_600_ was adjusted to 0.5.

### Mechanical inoculation

Viral mechanical transmissions were carried out as previously described [27]. For assays involving CiLV-C frameshift (fs) constructs, to ensure uniform inoculum pressure across the different fs constructs, 1g of infected plant tissue (leaf) was crushed in 3 mL of phosphate buffer (30mM, pH 8.0) for each sample infected with CiLV-C-p29fs, CiLV-C-p15fs, CiLV-C-p61fs, CiLV-C-MPfs, CiLV-C-p24fs, and CiLV-C-p15fs/p61fs. The resulting plant leaf extract was then gently rubbed onto the new *N. benthamiana* leaves 15 successive times, ensuring an even distribution. For viral mechanical serial passage assays, each passage was conducted 7 days post-inoculation (dpi).

**Plasmid** **construction** **(editing** **CiLV-C full-length** **cDNA clones)**

For engineering the pcDNA1-GFP-p29 carrying the GFP expression cassette inserted between the polymerase and *p29* genes (poly-GFP-p29), we designed primers containing overlapping fragments with the aid of the NEBuilder assembly tool (https://nebuilder.neb.com/). The binary vector containing the CiLV-C cDNA1 (pJL89 cDNA1, [27]) was used for inverse amplification with specific primers (S2 Table), which anneals to the 5’ end of the *p29* gene (cDNA1 Fwd) and the 3’end (cDNA1 Rev) of the gene junction (GJ) between polymerase and *p29* genes. For EGFP amplification, the 62 nt of the GJ was inserted upstream of the GFP sequence by two PCR rounds with each reverse primer (EGFP GJ 31pb Rev and EGFP GJ 62pb Rev) carrying part of the GJ sequence (S2 Table), generating the EGFP GJ 62nt insert. The EGFP gene was obtained from an alfalfa mosaic virus derivative infectious clone [64]. This strategy allowed us to duplicate the GJ and insert it before the EGFP and p29 sequences. Then, the EGFP carrying the complete GJ was amplified in a single step using primers (EGFP 25pb Fwd/EGFP 25pb Rev) with 25pb overlaps with 5’ and 3’ends of the binary vector pJL89 cDNA1. The amplifications were performed with the Phusion High-Fidelity DNA polymerase (New England BioLabs, Ipswich, MA, USA), and all fragments were purified using the Wizard SV Gel and PCR Clean-Up System (Promega, Madison, WI, USA). Subsequently, the fragments were assembled in an isothermal reaction (Gibson Assembly Cloning master mix, New England BioLabs, Ipswich, MA, USA), according to the manufacturer’s instructions. For insertion of the EGFP expression cassette after the *p29* gene, the pJL89 cDNA1 was used for inverse amplification with primers carrying *Nhe*I sites to facilitate the sub-cloning. These primers anneal at the 3’ end of the *p29* gene (cDNA1 Rev *Nhe*I) and the beginning of the CiLV-C RNA1 3’ UTR (cDNA1 Fwd *Nhe*I). The complete coding region of GFP, carrying the poly/p29 gene junction, was amplified from the pcDNA1-GFP-p29. The resulting PCR products were digested by *Nhe*I and ligated, resulting in the pcDNA1-p29-GFP plasmid. Clones with correctly oriented EGFP inserts were verified by colony PCR, and by Sanger sequencing (all constructs generated in this study were confirmed by these methods). To test the promoter activity of the poly/p29 GJ, we annulate the GJ 62nt downstream the EGFP by inverse amplification, which excluded this entire region of the new amplicon generated, using primers that anneals to the 3’ end of the polymerase (Poly+link2xser rev) and 5’ end of the p29 (EGFP Fwd). The PCR product, previously treated with *Dpn*I was phosphorylated, and blunt-end ligation was performed, generating the pcDNA1-ΔpGFP-p29 clone. To fuse the EGFP at the N- and C-terminus of the p29, the GJ before (poly-GFP-p29) and after (poly-p29-GFP) the p29 sequence was deleted by inverse amplification from pcDNA1-GFP-p29 and pcDNA1-p29-GFP plasmids, respectively. To ensure the fusion of the EGFP coding sequence with the N-terminal (EGFP:p29) and C-terminal (p29:EGFP) of the p29, stop codons of the EGFP and p29 were removed from primer sequences (S2 Table). Blunt-end ligation of PCR products generated the pcDNA1-GFP:p29 and pcDNA1-p29:GFP vectors.

The pcDNA1-p29fs, pcDNA2-p15fs, pcDNA2-p61fs, pcDNA2-MPfs, pcDNA2–24fs, and pcDNA2-p15fs/p61fs were derivate by primers that altered the gene coding sequence of these respective genes. For the p29fs mutant, a premature stop codon (TGA) was generated after the eighth codon. Similarly, for the p15 and p61 mutants, the stop codon (TGA) was generated after the sixth codon, and for the MP mutant, after the tenth codon. In the case of p24, due to the gene’s overlap with the 3’ end of the MP, we designed primes to delete two nucleotides (nt) in the second methionine (at position 25 in the codon sequence). This modification resulted in a premature stop codon occurring after the thirty-second codon (refer to the fs primers in the S2 Table). The pcDNA2-p15fs/p61fs construct was derived from the pcDNA2-p15fs clone using primes for p61fs (S2 Table). The PCR products, previously treated with *Dpn*I were phosphorylated, and blunt-end ligation was performed.

The pcDNA2-Δp15/DsRed, pcDNA2-Δp61/DsRed, pcDNA2-ΔMP/DsRed, and pcDNA2-Δp24/DsRed were derived from pcDNA2 by deletion of the entire transcription units of p15, p61, MP, and p24, respectively. The reverse amplification was performed with Rev and Fwd primers (S2 Table) carrying *Nco*I and *Xba*I, respectively, which are complementary to the regions before and after the start and stop codon of each gene (except for MP and p24). In the case of p24, to avoid modifying the coding sequence of MP, the Rev primer (Δp24 *Nco*I Rev) was designed to anneal to the 3’ end of MP, which corresponds to the 5’ end of p24, resulting in a construct with a synthetic intergenic region between MP and DsRed codon sequence consisting of two cytosines derived from the *Nco*I recognition site. In the case of the MP, the Fwd primer (ΔMP *Xba*I) anneals after the valine codon at position 288 of the MP codon sequence, to preserve the 5’end of the p24 gene. The complete coding region of DsRed was amplified with primers carrying *BspH*I and *Xba*I from a pSK plasmid [65], which are compatible with the amplicons carrying the *Nco*I and *Xba*I restriction sites above-mentioned.

To exchange the CiLV-C p29 by TCV CP, we proceeded to the golden gate strategy with primers (S2 Table) carrying the *Bsa*I type II restriction site containing sequences complementary to the region of insertion in the binary pJL89 cDNA1 vector. This vector was used for inverse amplification with primers (Δp29 *Bsa*I Fwd/ Δp29 *Bsa*I Fwd) designed to delete the entire p29 gene. TCV CP was amplified from a pTCV plasmid [66] using primers (TCV CP *Bsa*I [TCAT] Fwd and TCV CP *Bsa*I [CCGA] Rev) harboring *Bsa*I restriction site containing the sequences TCAT and CCGA, which are complementary to the amplified pJL89 cDNA1. The resulting PCR product was digested with *Bsa*I and ligated into the *Bsa*I-digested pJL89 cDNA1 to generate pcDNA1-Δp29/TCV-CP.

To exchange the GFP from pcDNA1-GFP-29 plasmid with the MPs of CiLV-C and TMV, we proceeded to the golden gate strategy with primers (S2 Table) carrying the *Bsa*I type II restriction site containing sequences complementary to the region of insertion in the binary pJL89 cDNA1 vector. This vector was used for inverse amplification with primers designed to delete the entire GFP gene. CiLV-C MP and TMV MP were amplified using primers harboring *Bsa*I restriction site containing the sequences *Nco*I and *Nhe*I, which are complementary to the amplified pJL89 cDNA1. The resulting PCR product was digested with *Bsa*I and ligated into the *Bsa*I-digested pJL89 cDNA1 to generate pcDNA1- CiLV-C MP and pcDNA1-TMV MP.

The pcDNA1-p29:HA plasmid used for *in vivo* protein-protein interaction analysis was generated from pJL89 cDNA1 by inverse PCR with the primer (cDNA1-p29:HA.3’ Fwd) carrying the human influenza hemagglutinin (HA) sequence (5’-TACCCATACGATGTTCCAGATTACGCT-3’). The PCR product was blunt-end ligated as above indicated. The same strategy was performed to generate pcDNA1-HA:p29, but using a primer (cDNA1-HA:p29.5’ Fwd) carrying the HA sequence.

The mutated version of the RNA1 carrying a premature stop codon in the CiLV-C polymerase was generated previously by (27).

### Plasmid construction (CoIP, individual transient expressions, and BiFC)

The binary pMOG800 constructs for co-immunoprecipitation (Co-IP) (pMOG-p29, pMOG-p29:HA, pMOG-TCV CP:HA, pMOG-3xMyc:PABP, pMOG-PABP:3xMyc, pMOG-3xMyc:eIF4A, pMOG-eIF4A:3xMyc, pMOG-3xMyc:eIF4E, and pMOG-eIF4E:3xMyc) and bimolecular fluorescence complementation (BiFC) assays (pMOG-NtYFP:PABP, pMOG-PABP:NtYFP, pMOG-CYFP:PABP, pMOG-PABP:CYFP, pMOG-NYFP:eIF4E, pMOG-eIF4E:NYFP, pMOG-CYFP:eIF4E, pMOG-eIF4E:CYFP, pMOG-NYFP:eIF4A, pMOG-eIF4A:NYFP, pMOG-CYFP:eIF4A, pMOG-eIF4A:CYFP, pMOG-NYFP:p29, pMOG-p29:NYFP, pMOG-CYFP:p29, pMOG-p29:CYFP, pMOG-NYFP:TCV CP, pMOG-TCV CP:NYFP, pMOG-CYFP:TCV CP, and pMOG-TCV CP:CYFP) were generated as detailed previously by [41,67]. pMOG-TCV CP and pMOG-CMV 2b (cucumber mosaic virus) plasmids, used *in trans*-expression analysis, were generously provided by Dr. José Antonio Navarro from the IBMCP institute (UPV, Valencia, Spain). pMOG Lep (Leader peptidase protein, WP_112485673.1) an integral component of bacterial membranes, and pMOG TEV HCPro were obtained from [68]. The p19 gene (AJ288941.1) from TBSV was obtained from PCR amplification with primers containing *Nco*I and *Nhe*I restrict sites and subsequently cloned between CaMV 35S promoter and potato proteinase inhibitor terminator (PoPit) into the pMOG binary vector.

### Plasmid construction (GST:p29 expression)

For northwestern analysis, the CiLV-C p29 glutathione S-transferase (GST)-tagged expression cassette was obtained from the double-digestion of the pGEX-KG plasmid (GE Healthcare Life Science) with *Nco*I and *Sal*I (Fisher Scientific, Waltham, MA, USA) restriction enzymes, generating compatibility with CiLV-C p29 PCR product containing combative cohesive ends. The *BspH*I-*Sal*I-digested p29 PCR product was ligated in the previously *Nco*I-*Sal*I-digested pGEX-KG, resulting in the pGEX-KG-GST:p29 plasmid. Mutated p29 versions (ΔHR and Δ155–175) were generated from pGEX-GST:p29 vector by inverse PCR.

### Plasmid construction (virus-induced gene silencing, VIGS)

Tabacco rattle virus (TRV)-based VIGS was used to knockdown the expression of *N. benthamiana eIF4A* gene (NbeIF4A, KP979608.1). A 300-bp fragment (166–465 bp) of the NbeIF4A coding sequence was amplified by RT-PCR with specific primers carrying attB sequence (S2 Table). The attB-flanked PCR product was inserted into pDONR 207 plasmid by BP Gateway reaction (Thermo Fisher Scientific, Waltham, MA, USA) following manufacturer’s specifications. Through the LR Gateway reaction, the NbeIF4A sequence was transferred into the pTRV2 plasmid. The methodology for generating constructs in the AMV VIGS system is detailed by [40]

### RNA extraction

Total RNAs from agroinfiltrated (at 7 dpi) or mechanically inoculated (at 8 dpi) *N. benthamiana* leaves were extracted using VWR Life Science AMRESCO RiboZol RNA extraction Reagent.

### Poly Tail addition

Poly (G) and poly (U) addition was performed using Poly (A) and Poly (U) polymerase (New England BioLabs, Ipswich, MA, USA) following the manufacturer’s instructions. Briefly, the RNA sample (minimum amount 10 ng) was incubated in a final volume of 12.5 μl, containing 2.5 μl of 5X buffer, 0.65 μl of 10 mM GTP or UTP, and 1 μl of polyA/U polymerase for 20 minutes at 37 °C.

### Northern blot

All procedures of RNA electrophoresis, membrane transference, hybridization, and detection were conducted as previously described by [6,69]. For CiLV-C genomic (g), subgenomic (sg), and anti-genomic (vc) RNA detection, CiLV-C RNA1 and RNA2 DIG-riboprobes (Roche, Mannheim, Germany) were generated as described by (27). For the detection of TCV, a DIG-probe complementary totheTCV *CP* gene was generated. Northern blot analysis from agroinfiltrated and mechanically inoculated leaves expressing CiLV-C infectious clone and its mutated versions was performed using 3–6 μg of total RNA. The quantification of the RNA bands was performed using ImageJ version 2.0cr software with the ISAC plugin.

### Total protein extraction and western blot

Total protein was isolated from infiltrated and inoculated plant leaves with Berger buffer containing 750 mM Tris-HCl, pH 8.8, 4% (w/s) SDS, 4% (w/s) β-Mercaptoethanol, and 40% (w/s) saccharose. The protein expression was confirmed by Western blot on 12% SDS-PAGE as described previously [4], using the primary monoclonal antibodies anti-GFP (1:10000) (Thermo Fisher Scientific, Waltham, MA, USA), anti-HA (1:10000), and anti-c-Myc (1:5000) (Sigma-Aldrich, Steinheim, Germany) followed by anti-mouse or anti-habit IgG HRP-linked antibodies (1:10000) (Sigma-Aldrich, Steinheim, Germany).

### *In vivo* CoIP

For Co-immunoprecipitation assays from transient expression context, the HA tag was introduced into the coding sequence of the CiLV-C p29 (YP_654539.1), while 3xMyc tags were introduced into the coding sequence of the PABP (Q347293.1), eIF4E (DQ393833.1), and eIF4A (NM_001324828). The CoIP assay was conducted using the HA epitope and the Pierce Magnetic HA-Tag kit (Thermo Fisher Scientific, Waltham, MA, USA), following the manufacturer’s guidelines. At 3 dpi, 0.1 g of tissue was homogenized with liquid nitrogen with 0.5 mL of non-denaturing buffer (25 mM Tris HCl pH 7.4, 150 mM NaCl, 1 mM EDTA, 1% NP-40, 5% glycerol). The extracts were incubated on ice for 5 minutes and then centrifuged at 13,000 x *g* for 10 minutes to remove cell debris. 50 µl of the supernatant was used for the protein expression control (C+) meanwhile the rest of the supernatant was incubated with 20 µl (0.2 mg) of Pierce Anti-HA Magnetic Beads during 90 minutes of agitation at room temperature. After the washing steps (3X) with the non-denaturing buffer, the HA immunoprecipitated proteins were eluted by resuspending the beads with 70 µl of 1X Laemmli buffer and incubate them at 100 ºC for 10 minutes. Western blot analyses were performed using 20 µl of C+ and 30 µl of IP proteins per line and two monoclonal anti-HA and anti-c-Myc antibodies (Sigma-Aldrich, Steinheim, Germany), following the manufacturer’s instructions. For the CoIP assay, from virus infection context, the CiLV-C-p29:HA (pcDNA1-p29:HA + pcDNA2) construct carrying the HA epitope at the C-terminus of the p29 protein, was agroinfiltrated onto *N. benthamiana* leaves. The CiLV-C infectious clone wild-type (WT) (pcDNA1 + pcDNA2, which corresponds to a non-epitope version) was used as a control. At 12 dpi, 12 leaf discs (0.14 g) of the symptomatic infected tissue were crushed in liquid nitrogen, then added 0.5 mL of denaturing buffer. The subsequent steps were identical to the CoIP described above. After the elution step in 1x Leammli buffer, part of the IP fraction eluted was sent for mass spectrometry sequencing at the proteomic service of the University of Córdoba.

### GST:p29-Expression

CiLV-C p29 was subcloned into pGEX-KG (GE Healthcare Life Science) to generate a cassette with p29 fused to the C-terminal of the GST. Free GST and GST-tagged proteins were expressed in BL21 (DE3) *E. coli* by induction with IPTG (1 mM).

### Analysis of GST:p29-Viral RNA interaction by northwestern

Northwestern analysis was carried out as previously described [70]. Two different concentrations (1:4 dilution factor) of GST:p29, mutated versions or non-fused GST (control) expressed proteins were electrophoresed in 12% SDS-PAGE and transferred to PVDF membranes. Membranes were incubated overnight at 4°C in renaturation NW buffer (10 mM Tris-HCl, pH 7.5; 1 mM EDTA; 0.1 M NaCl; 0.05% TRITON-X100; and 1% BSA). Next, the membranes were incubated with 20 mL of NW buffer (without BSA) containing DNase-treated CiLV-C RNAs 1 and 2 (1 µg) labeled with digoxigenin (DIG RNA Labeling Mix, Roche, Mannheim, Germany) for 2 h at room temperature, followed by three washes with NW buffer + Tween 0.3%. Then, the membranes were incubated for 1 h in 20 mL of NW buffer containing anti-DIG (1:10000) (Anti-digoxigenin-AP, Fragments Fab; Roche, Mannheim, Germany), followed by three washes. The membranes were revealed using BCIP/NBT color development substrate (Thermo Fisher Scientific, Waltham, MA, USA) following the manufacturer’s guidelines.

### *In vitro* transcription for digoxigenin-labelled riboprobes and virus rescue

Specific regions of CiLV-C RNAs 1 (*p29* gene) and 2 (*p24* gene) were amplified with primers carrying the T7 promoter sequence. For TCV probe, the *CP* gene was amplified with primers carrying T7 sequence. The PCR products were used directly as templates to transcribe the synthetic viral RNAs using T7 RNA polymerase (Takara Bio Inc, USA) and nucleotide DIG-11-UTP-labeled mix solution (Roche, Mannheim, Germany), following the manufacturer’s instructions.

To generate TCV transcripts for plant infection, approximately 1 µg of pTCV plasmid [34] carrying T7 promoter was digested with *Sma*I. The linearized pTCV plasmid was used directly as a template to transcribe the synthetic viral RNAs using T7 RNA polymerase (Takara Bio Inc, USA) following the manufacturer’s instructions. RNA transcript quantifications were performed with agarose gel electrophoresis using an RNA ladder (RiboRuler High Range RNA Ladder, Thermo Fisher Scientific, Waltham, MA, USA) and several dilutions of the transcribed RNAs, such as described by (65).

### Bimolecular fluorescence complementation (BiFC) assay

For the BiFC assay addressed to investigate the interaction between the p29 with eIF4A, eIF4E, and PABP, the p29 protein carrying the NYFP or CYFP fused at the N-or C-terminus was transiently expressed with the counterpart fused to the N- or C-terminus of the eIF4A, eIF4E, and PABP or with NYFP alone (Ncyt) or carrying ER signal label (Cer) as controls. This same approach was conducted with the TCV CP. All procedures for BiFC assays were conducted as previously reported by (67). The YFP fluorescence was captured at four dpi. Four leaves per plant and three plants per construct or combination of constructs were analyzed in three independent experiments. All confocal images were obtained after multiple visualizations of different cells and regions of the *N. benthamiana* leaves.

### Co-localization assay and nucleus markers

To investigate the intracellular sublocalization of p29 and eIF4A, *N. benthamiana* leaves were agroinfiltrated with either the pMOG-p29:GFP or pMOG-eIF4A fused to mCherry N- or C-terminus. BiFC constructs of p29 and eIF4A were co-infiltrated with the nucleus marker SV40-RFP [71]. To assess the interaction between p29 and eIF4A during the viral infection cycle, leaves were co-agroinfiltrated with either pCiLV-C-GFP:p29 or pCiLV-C-p29:GFP infectious constructs, along with pMOG-mCherry:eIF4A or pMOG-eIF4A:mCherry. For *A. tumefaciens* cultures carrying the nuclear marker, the OD_600_ was adjusted to 0.1. For *Agrobacterium* cultures harboring CiLV-C infectious clones and their derivative constructs, the above-mentioned infiltration specifications were followed. The fluorescence was monitored from 2 to 7 dpi, with the images captured at 5 dpi. For leaves infiltrated with p29 and eIF4A, the images were taken at 3 dpi. All confocal images were obtained after multiple visualizations of different cells and regions of the *N. benthamiana* leaves to ensure consistency and reproducibility.

### Fluorescence loupe and confocal laser scanning microscopy

Using a fluorescence loupe MacroFluo (MZ16F Leica) with GFP and RFP filters, we captured the fluorescence from *N. benthamiana* leaves infected with the engineered CiLV-C infectious clone expressing GFP and/or DsRed. Using a laser scanning microscope Zeiss LSM 780 AxiObserver, we captured the fluorescence from several leaf discs of *N. benthamiana* plants. GFP was excited at 488 nm, and emission was captured at 495–520 nm. YFP was excited at 515 nm, and emission was captured at 520–560 nm. DsRFP was excited at 552 nm, and emission was captured at 585–610 nm. mCherry was excited at 545 nm, and emission was captured at 572–595 nm. Image processing was performed using the Fiji Image J program version 2.0r.

**Transmission** **electron** **microscope (TEM)**

Small pieces of *N. benthamiana* leaves infected with CiLV-C derivative infectious clones were fixed in a mixture of 2% glutaraldehyde, and 2.5% paraformaldehyde in 0.05 M cacodylate buffer pH 7.2. All procedures for block sectioning, thin-section sample preparation, and TEM visualization were performed as previously described by [27].

### Virus-induced gene silencing (VIGS) assay

For VIGS analysis, *N. benthamiana* plants (8 plants for each treatment) were agroinfiltrated with a mix of bacterial cultures harboring TRV1 with TRV2-eIF4A or TRV2-empty (control). At 10 dpi of TRV, silenced plants were inoculated with a mechanical inoculum of rCiLV-C-GFP or TCV. At 7 days post mechanical inoculation, total RNA was extracted to analyze vRNA and eIF4A mRNA accumulation by northern blot and RT-qPCR, respectively. VIGS analysis using the AMV system was performed at the same time points as indicated for TRV.

### Real-time quantitative reverse transcription (RT-qPCR)

At 17 dpi with TRV, total RNA from leaf tissues was extracted. First-strand cDNA was synthesized by using the oligo dT (20) primer and RevertAid H Minus reverse transcriptase (ThermoFisher Scientific, Waltham, MA, USA) following the manufacturer’s instructions. KAPA SYBR FAST qPCR master mix, 2 µ of cDNA synthesized, and specific primers (S2 Table) were used for RT-qPCR analysis of NbeIF4A mRNA levels with a QuantStudio 3 Real-Time PCR (Applied Biosystems). To normalize the mRNA levels of target genes between samples, relative mRNA levels of the *L23* gene were determined with specific primers previously published [72].

### Generative AI usage statement

During the preparation of this work, the authors utilized deep seek AI for language refinement. After using this tool/service, the authors carefully reviewed and revised the content where necessary, and take full responsibility for the final version of the publication.

## Supporting information

S1 Fig Schematic diagram of CiLV-C RNA1 from the WT construct and the recombinant version carrying the GFP sequence inserted between the polymerase and p29 genes. Chromatograms obtained by Sanger sequencing of amplicons corresponding to the 5′ terminal ends are aligned with the viral genome sequence. The red line indicates the poly (G) and poly (U) tails added to the 5’ end of the subgenomic viral RNA. The black line denotes the 5’ UTR of the two subgenomic RNAs. Gray and green lines indicate the open reading frames of the p29 and GFP genes, respectively.
(TIF)

S2 Fig The CiLV-C RNA1 spread locally through the action of MP. (A) *Trans*-complementation of pcDNA1-GFP cell-to-cell movement by the ectopic MP expression from a pMOG binary vector. A schematic genomic representation of rCiLV-C and the pMOG binary vector, carrying the CiLV-C MP fused to the HA epitope at its C-terminus, is shown at the top of the panels. The GFP signal in *N. benthamiana* leaves was captured at 3, 6, and 10 dpi using a fluorescence loupe. Scale bar, 200 μm. (B) Western blot analysis at 5 dpi of GFP and HA expression in plants infiltrated with pcDNA1-GFP (line 1) and pcDNA1-GFP + pMOG-MP:HA (lane 2). (-) corresponds to a non-infected plant. The samples were analyzed using antibodies against HA and GFP. Coomassie blue-stained proteins indicate equal sample loading.
(TIF)

S3 Fig The insertion of the movement protein gene into CiLV-C RNA1 preserves its ability for local movement. (A) Total RNA from *N. benthamiana* leaves infected with pcDNA1 wildtype (WT) and recombinants carrying the CiLV-C MP (pcDNA1-CiLV-C MP) and TMV MP (pcDNA1-TMV MP) was analyzed at 6 dpi by northern blot using a DIG-labelled riboprobe complementary to the CiLV-C *p29* gene. The localization of CiLV-C gRNA1, sgRNA1, and sgMP are indicated. Ethidium bromide-stained rRNA serves as a loading control. (B) GFP fluorescence signal in *N. benthamiana* leaves infiltrated with pcDNA1-WT + pcDNA2-ΔMP/GFP (a), pcDNA1-CiLV-C MP + pcDNA2-ΔMP/GFP (b), and pcDNA1-TMV MP + pcDNA2-ΔMP/GFP (c). A schematic genomic representation of rCiLV-C is shown at the top of the panels. The GFP signal in *N. benthamiana* leaves was captured at 10 dpi using a fluorescence loupe. Scale bar, 500 μm.
(TIF)

S4 Fig Onset of symptoms induced by frameshift deletions in CiLV-C infectious clone. *N. benthamiana* leaves agroinfiltrated with CiLV-C constructs (pCiLV-C and rCiLV-C-GFP) carrying frameshift deletions in p29, p15, p61, MP, p24, and p15-p61 ORFs. Leaf images were captured at 10 and 20 dpi. At 15 dpi no necrosis lesions were observed on upper non-inoculated leaves (pictures below).
(TIF)

S5 Fig Comparative analysis of CiLV-C RNA accumulation from *N. benthamiana* leaves agroinfiltrated versus mechanically inoculated. Leaves were agroinfiltrated with CiLV-C constructs carrying frameshift mutation in p29, p15, p61, MP, p24, and p15-p61. These infiltrated leaves were used as a source of inoculum for the mechanical inoculation of new plants, which were then analyzed for viral RNA accumulation. Northern blot (at 6 dpi) using DIG-probes complementary to the CiLV-C p29 and p24. The localization of CiLV-C gRNA1, sgRNA1, gRNA2, and sgRNA4 are indicated. rRNA stained with ethidium bromide indicates equal loading of samples. A negative control that corresponds to a non-infected plant is displayed in the final lane of the northern blots. RNA band intensity was quantified using Fuji software with the ISAC plugin and represented by the graphic. Graphics represent the relative accumulation (arbitrary units, AU) of CiLV-C RNAs. Data are the mean of three independent biological replicates. Error bars represent SD. An asterisk indicates a statistically significant difference according to unpaired Student’s t-test (two-tailed), *** *p *< 0.001, ** *p* < 0.01, * *p* < 0.05, and ns corresponds to *p* > 0.05.
(TIF)

S6 Fig Electron micrographs of thin sections of *N. benthamiana* leaves infected with pCiLV-C-WT, pCiLV-C-p15fs, pCiLV-C-p61fs, pCiLV-C-MPfs, pCiLV-C-p24fs, pCiLV-C-p15fs/p61fs, and rCiLV-C-GFP. Structures similar to viroplasm-like (Vp) are indicated. Scale bars, 2 μm, 1 μm, and 500 nm.
(TIF)

S7 Fig Measurement of the virion length in wild-type and mutant CiLV-C particles. The length of virions was measured for wild-type CiLV-C (pCiLV-C-WT) and CiLV-C particles carrying frameshift mutations in p15 (pCiLV-C-p15fs), p61 (pCiLV-C-p61fs), and both p15-p61 (pCiLV-C-p15fs/p61fs), as well as for recombinant CiLV-C expressing the GFP (rCiLV-C-GFP). Electron micrographs of thin sections of *N. benthamiana* leaves infected with the respective constructs were analyzed. Mature enveloped virions were observed and measured. Bars represent the mean virion lengths, with sample sizes of n = 25 for all groups, except for p15fs/p61fs (n = 12). Error bars represent SD. *p* indicates a statistically significant difference according to unpaired Student’s t-test (two-tailed). n.s indicates no significant difference. *p* < 0.001 indicates a significant difference.
(TIF)

S8 Fig Replacement of the p29 gene with the TCV CP gene did not preserve virus infection. The p29 gene was replaced by the coat protein (CP) gene of turnip crinkle virus (TCV), generating the pcDNA1-Δp29/TCV CP construct. (A) *N. benthamiana* leaves were co-infiltrated with pcDNA1-Δp29/TCV CP + pcDNA2-Δp24/DsRed (a construct in which the *p24* gene was replaced by DsRed) (a), pcDNA1-p29fs + pcDNA2-Δp24/DsRed (b), and pcDNA1-WT + pcDNA2-Δp24/DsRed (c). RFP signal was monitored for up to 15 dpi, and images were taken at 10 dpi. Bars correspond to 500 μm. (B) Northern blot analysis of RNA extracted from leaves of four plants (p1 to p4) agroinfiltrated with rCiLV-C-Δp29/TCV CP (pcDNA1-Δp29/TCV CP + pcDNA2) and pCiLV-C-WT (pcDNA1-WT + pcDNA2). DIG-probes complementary to the TCV *CP* and CiLV-C *p24* genes were used. (-) corresponds to a non-infected plant. Ethidium bromide staining of rRNA indicates equal loading of samples. The localization of CiLV-C gRNA1, gRNA2, sgRNA1, and sgRNA4 is indicated. The large membrane piece (on the left) containing the rCiLV-C-Δp29/TCV CP samples was highly exposed to visualize the bands.
(TIF)

S9 Fig The insertion of HA-tag at the C-terminal of p29 does not impair the movement, replication, and mechanical transmission of CiLV-C. (A) Analysis of cell-to-cell movement of pcDNA1-WT, and recombinant versions carrying HA-Tag fused to the N- or C-terminus of the p29 (pcDNA1-HA:p29 and pcDNA1-p29:HA, respectively). DsRed fluorescence was captured at 12 dpi. *N. benthamiana* leaves were mechanically inoculated (1^st^ pass) with sap extract from infiltrated leaves, and cell-to-cell movement was monitored at 6 dpi. (B) Northern blot analysis of CiLV-C RNAs 1 and 2 accumulation at 6 dpi, from *N. benthamiana* leaves agroinfiltrated with pcDNA1-WT + pcDNA2 (CiLV-C WT) and pcDNA1-p29:HA + pcDNA2 (CiLV-C HA) using a DIG-probe complementary to the CiLV-C *p29* and *p24* genes. The negative control (-) corresponds to a non-infected plant. Ethidium bromide staining of rRNA indicates equal loading of samples. The localization of CiLV-C gRNA1, gRNA2, sgRNA1, and sgRNA4 is indicated. The graphs show the mean relative accumulation (AU) of total CiLV-C RNAs from two independent biological replicates. (C) Immunoprecipitation and western blot analysis of CiLV-C p29:HA. Immunoprecipitation of CiLV-C p29 was analyzed by western blot using an anti-HA antibody. The results show the accumulation of the p29:HA protein obtained from tissue inoculated with the unlabeled version (pcDNA1-WT + pcDNA2, CiLV-C WT) and the HA-tagged version (pcDNA1-p29:HA + pcDNA2, CiLV-C HA), before (control) and after (CoIP) immunoprecipitation. The protein size, expressed in kDa, is shown on the right side of the image.
(TIF)

S10 Fig Ontological analysis of the proteins immunoprecipitated with the CiLV-C p29:HA. (A) Ontological distribution. Graphs corresponding to the three ontological categories: biological process, molecular function, and cellular component, are shown. Each graph displays the top 20 subcategories into which the analyzed protein set was classified. The proportion of proteins assigned to each category is indicated in the pie chart. Note that a single protein can be assigned to multiple subcategories. (B) Ontological count of molecular function. The graph illustrates the top 30 subcategories assigned into the molecular function. The red arrow highlights the translation initiation factor subcategory. The ontological analyses were generated using the Omicsbox 3.2.2 software.
(TIF)

S11 Fig BiFC and CoIP assays to prove the interaction between p29 and PABP/eIF4E. (A) *In vivo* interaction between the p29 and eIF4E/PABP. PABP and eIF4E were targeted at their C-terminus with NYFP or CYFP, respectively, and co-expressed with p29 fused at its N- or C-terminus with the NYFP or CYFP. A representative protein pair combination is indicated at the top of the image. The image shows the reconstitution of the YFP fluorescence distributed through the cell cytoplasm of *N. benthamiana* cells. The image is representative of several infiltrated leaves from three different plants. The fluorescent signals were captured at 3 dpi. The green (GFP), transmitted light (T.L) channels, and merged images are shown in the figure. The negative control is illustrated by a representative image (mock) showing the expression of eIF4A, eIF4E, and PABP combined with Ncyt constructs and p29 combined with the Cer construct. The bars indicate 50 μm. (B) CoIP of CiLV-C p29 with eIF4E and PABP. Extracts of *N. benthamiana* leaves expressing p29 fused at its N- or C-terminus with an HA epitope and 3xMyc targeted eIF4E or PAPB at their N- or C-terminus, were analyzed at 3dpi. The CoIP assay was addressed using the Pierce HA-Tag Magnetic IP/CoIP Kit. Western blot analysis was carried out using Myc and HA antibodies. C + , positive control (non-immunoprecipitated samples); IP, immunoprecipitated samples. ‘+’ and ‘–‘ signs indicate the presence or absence of the corresponding proteins in the leaf extracts.
(TIF)

S12 Fig Overexpression of eIF4A does not rescue a p29 defective CiLV-C clone. *N. benthamiana* leaves were co-infiltrated with pcDNA1-GFP-p29fs and either: pMOG-eIF4A (a), pMOG-Lep (b), or pMOG-p29 (c). GFP signal was captured at 5 dpi. Scale bars represent 500 μm.
(TIF)

S13 Fig TCV CP interaction with eIF4A and the effect of *N. benthamiana* eIF4A overexpression and knockdown on TCV infection. (A) *In vivo* interaction between the TCV CP and eIF4A. The eIF4A was targeted at its C-terminus with NYFP or CYFP and co-expressed with the TCV CP fused at either its N- or C-terminus with the NYFP or CYFP. A representative protein pair combination is indicated at the top of the image. The image shows the reconstitution of the YFP fluorescence into the nuclei and distributed through the cell cytoplasm of *N. benthamiana* leaves. The image is representative of several infiltrated leaves from three different plants. The fluorescent signals were captured at 3 dpi. The green (GFP), transmitted light (T.L) channels, and merged images are shown in the figure. The bars indicate 50 μm. (B) CoIP of TCV CP with eIF4A. Extracts of *N. benthamiana* leaves expressing the TCV CP fused at either the N- or C-terminus with an HA epitope, along with eIF4A tagged at the N- or C-terminus with 3 × Myc, were analyzed at 3dpi. The CoIP assay was addressed using the Pierce HA-Tag Magnetic IP/CoIP Kit. Myc and HA antibodies were used in the western blots. C + , positive control (non-immunoprecipitated samples); IP, immunoprecipitated samples. “+” and “–” signs indicate the presence or absence of the corresponding proteins in the leaf extracts. (C) Left panel: northern blot analysis (3 dpi) showing the levels of TCV genomic (g) and subgenomic (sg) RNAs in plants overexpressing eIF4A or DsRed (control) infected with TCV. RNA detection was performed using a DIG-probe complementary to the TCV *CP* gene. The localization of TCV gRNA and sgRNA is indicated. Ethidium bromide-stained rRNA serves as a loading control. Right panel: graph showing the mean relative accumulation levels of TCV RNA from plants expressing eIF4A or DsRed. Error bars represent SD. Asterisks indicate statistically significant differences according to unpaired Student’s t-test (two-tailed), ** *p* < 0.01. (D) Northern blot analysis of TCV RNA accumulation in plants control (TRV-empty) and eIF4A-silenced (TRV-NbeIF4A) infected with TCV at 7 days post-TCV mechanical inoculation and 17 days post-TRV agroinfiltration. The localization of TCV gRNA and sgRNA is indicated in the northern blot. Ethidium bromide-stained rRNA serves as a loading control. (E) The graphic represents eIF4A mRNA level transcripts in TRV-empty and TRV-NbeIF4A infected *N. benthamiana* plants. *L23* housekeeping gene served as an internal control. Bars indicating standard error from 8 plants per treatment.
(TIF)

S14 Fig Necrotic lesion by CiLV-C infection occurs independently of the vRNA2 component. *N. benthamiana* leaves agroinfiltrated with pcDNA1-GFP + pcDNA2, pcDNA1-GFP, pcDNA1-GFP + pMOG-MP, pcDNA1-GFP + pcDNA2-Δp61/DsRed, and pcDNA2 at 15 dpi. To determine whether RNA1 plays a direct role in the necrotic lesions, we infiltrated leaves with *Agrobacterium* cultures transformed only pcDNA1-GFP and bacteria combination carrying pcDNA1-GFP + pMOG-MP, which favors increased accumulation of RNA1 due to increased dissemination. To determine whether p61 (both the protein and gene) plays a direct role in the necrotic lesions, leaves were infiltrated with rCiLV-C-Δp61/DsRed, which the p61 sequence was replaced by DsRed. Necrotic reaction distributed through the infiltrated tissue is exhibited in all constructs, except in leaves infiltrated with only pcDNA2, indicating that RNA1 is enough to trigger the necrosis response and that p61 is not directly associated with this phenotype. Leaves under UV light reveal necrosis reaction in zones infected by rCiLV-C constructs. Intense autofluorescence under RFP filter indicates necrosis regions. Yellow arrows show autofluorescence signal, while white arrows show GFP fluorescence signal from CiLV-C infection. Fluorescence loupe images show the transmitted light (TL), GFP filter, RFP filter, and merged images. Scale bars correspond to 500 μm, 1 mm, and 2 mm.
(TIF)

S1 Table List of the identified p29-interacting proteins. (XLSX)

S2 Table Primers used in this study. (DOCX)

S1 Data Source data for graphs in this study. (XLSX)

S2 Data Original images. (DOCX)
