# Supplementary figures and images for: From darkness to light: Genetic manipulation of an atypical plant virus unveils key insights into kitavirus biology, highlighting capsid protein and eIF4A engagement to drive viral infection
Source: PLoS Pathog. 2025 Aug 1;21(8):e1013388. doi: 10.1371/journal.ppat.1013388 (PMC12334043; doi:10.1371/journal.ppat.1013388)

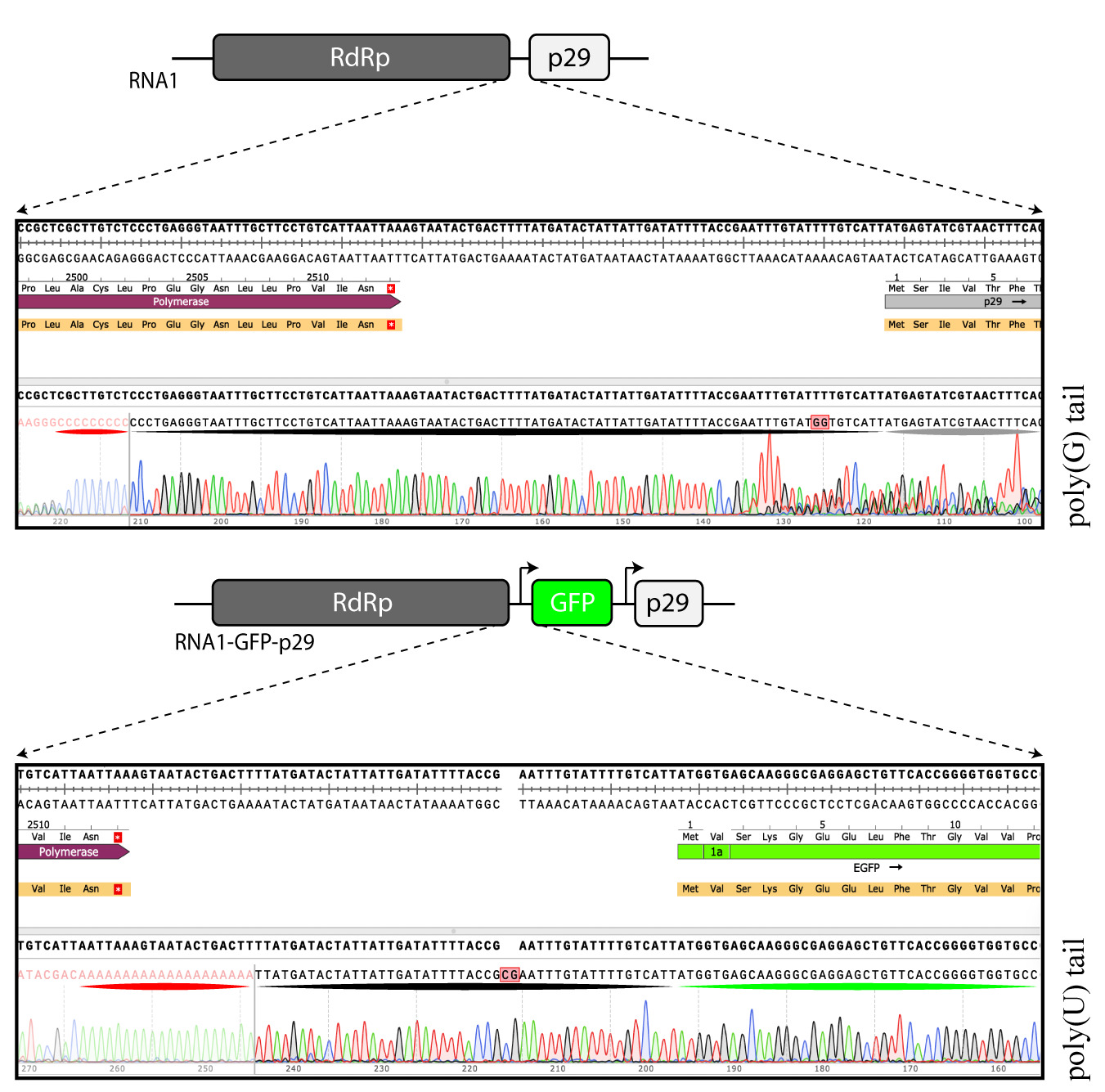

Supplement: S1 Fig — Chromatograms obtained by Sanger sequencing of amplicons corresponding to the 5′ terminal ends are aligned with the viral genome sequence. The red line indicates the poly (G) and poly (U) tails added to the 5’ end of the subgenomic viral RNA. The black line denotes the 5’ UTR of the two subgenomic RNAs. Gray and green lines indicate the open reading frames of the p29 and GFP genes, respectively. (TIF) [file ppat.1013388.s001.tif]

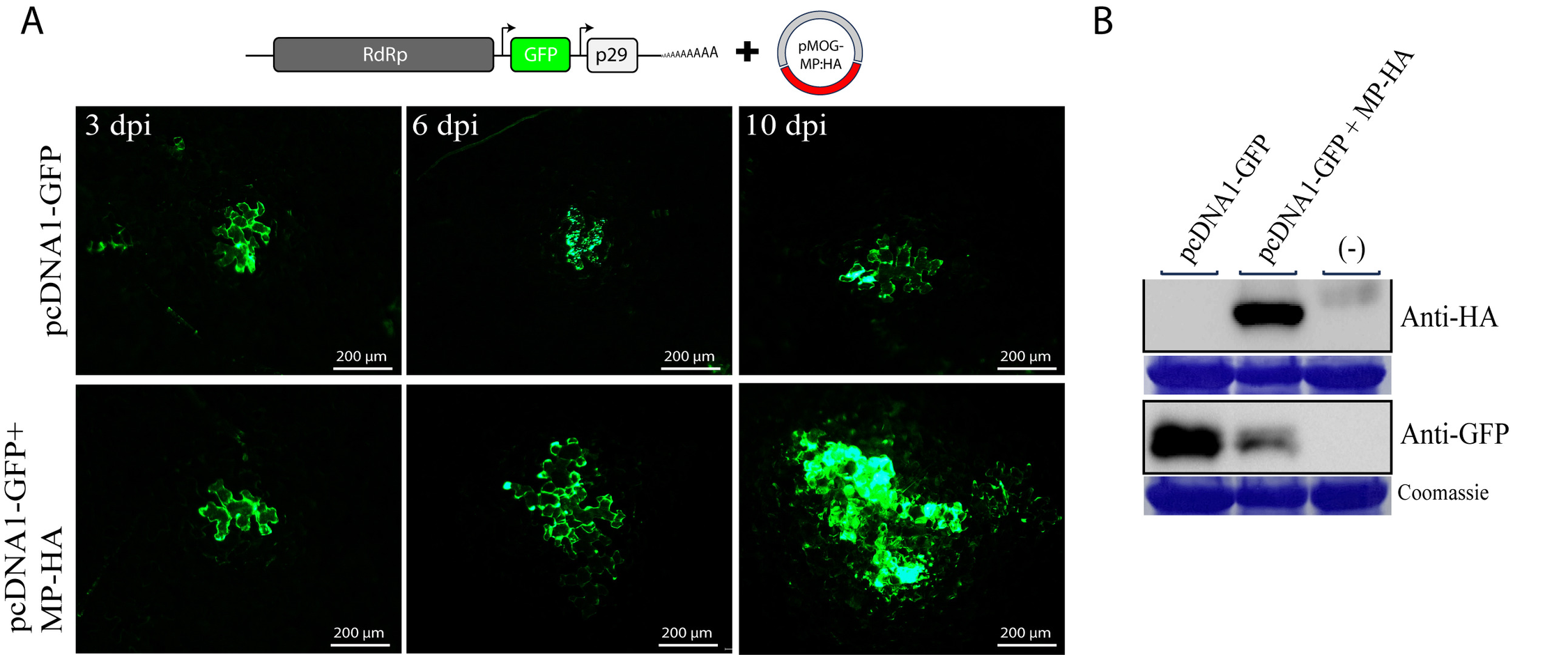

Supplement: S2 Fig — (A) Trans-complementation of pcDNA1-GFP cell-to-cell movement by the ectopic MP expression from a pMOG binary vector. A schematic genomic representation of rCiLV-C and the pMOG binary vector, carrying the CiLV-C MP fused to the HA epitope at its C-terminus, is shown at the top of the panels. The GFP signal in N. benthamiana leaves was captured at 3, 6, and 10 dpi using a fluorescence loupe. Scale bar, 200 μm. (B) Western blot analysis at 5 dpi of GFP and HA expression in plants infiltrated with pcDNA1-GFP (line 1) and pcDNA1-GFP + pMOG-MP:HA (lane 2). (-) corresponds to a non-infected plant. The samples were analyzed using antibodies against HA and GFP. Coomassie blue-stained proteins indicate equal sample loading. (TIF) [file ppat.1013388.s002.tif]

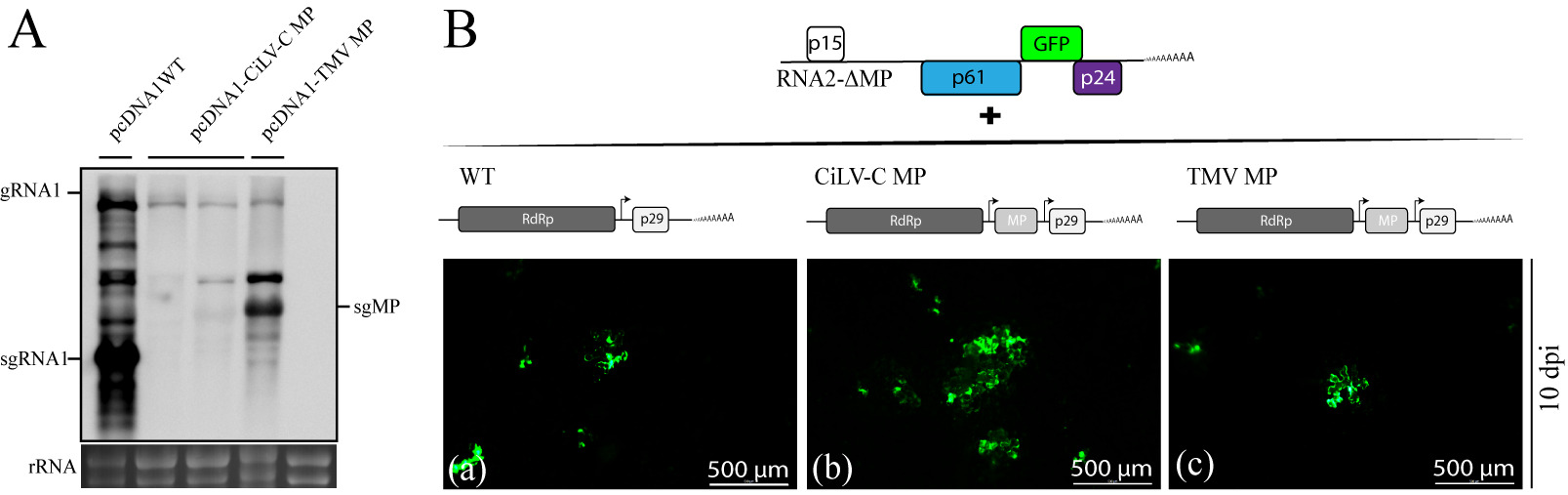

Supplement: S3 Fig — (A) Total RNA from N. benthamiana leaves infected with pcDNA1 wildtype (WT) and recombinants carrying the CiLV-C MP (pcDNA1-CiLV-C MP) and TMV MP (pcDNA1-TMV MP) was analyzed at 6 dpi by northern blot using a DIG-labelled riboprobe complementary to the CiLV-C p29 gene. The localization of CiLV-C gRNA1, sgRNA1, and sgMP are indicated. Ethidium bromide-stained rRNA serves as a loading control. (B) GFP fluorescence signal in N. benthamiana leaves infiltrated with pcDNA1-WT + pcDNA2-ΔMP/GFP (a), pcDNA1-CiLV-C MP + pcDNA2-ΔMP/GFP (b), and pcDNA1-TMV MP + pcDNA2-ΔMP/GFP (c). A schematic genomic representation of rCiLV-C is shown at the top of the panels. The GFP signal in N. benthamiana leaves was captured at 10 dpi using a fluorescence loupe. Scale bar, 500 μm. (TIF) [file ppat.1013388.s003.tif]

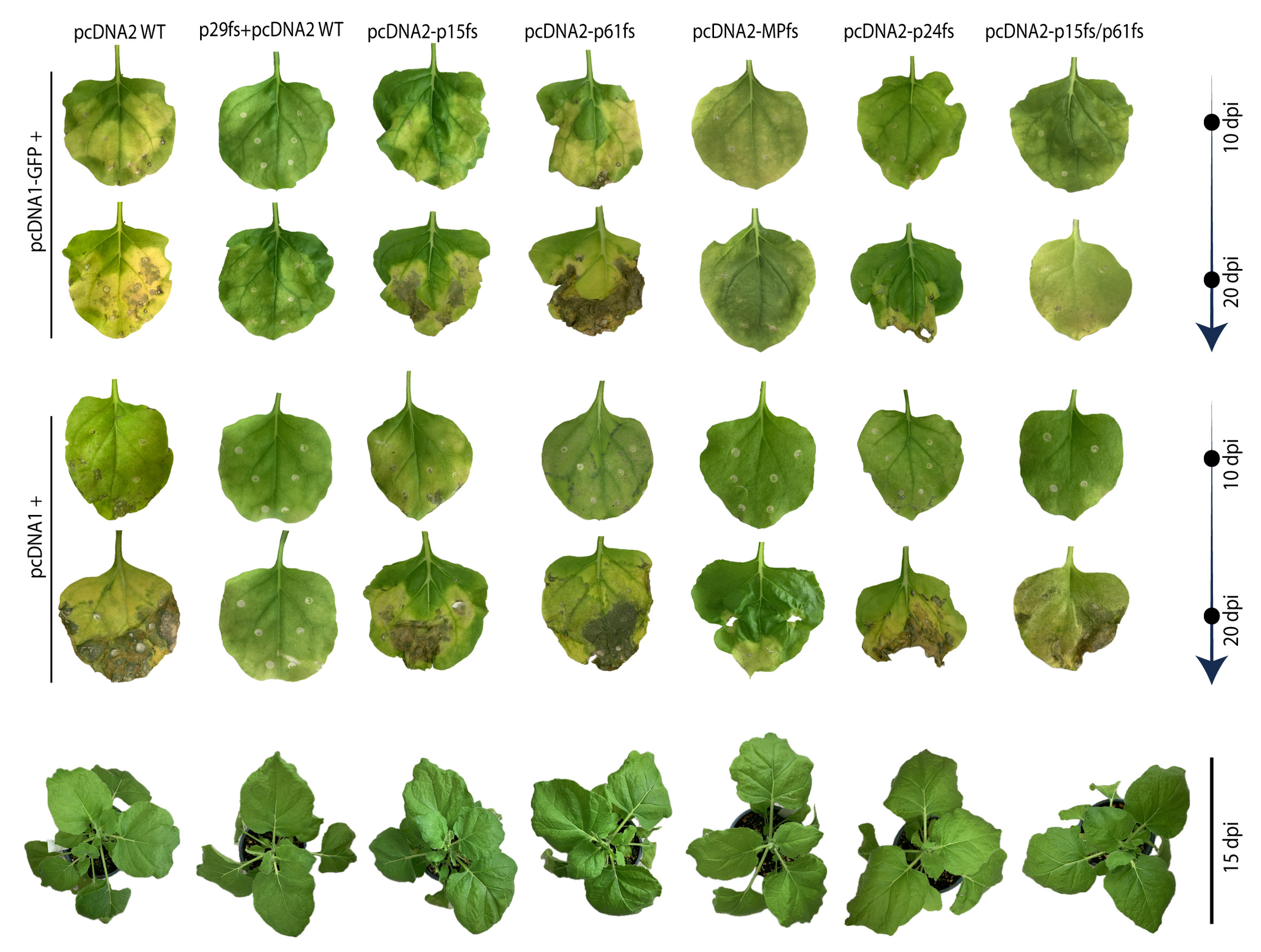

Supplement: S4 Fig — N. benthamiana leaves agroinfiltrated with CiLV-C constructs (pCiLV-C and rCiLV-C-GFP) carrying frameshift deletions in p29, p15, p61, MP, p24, and p15-p61 ORFs. Leaf images were captured at 10 and 20 dpi. At 15 dpi no necrosis lesions were observed on upper non-inoculated leaves (pictures below). (TIF) [file ppat.1013388.s004.tif]

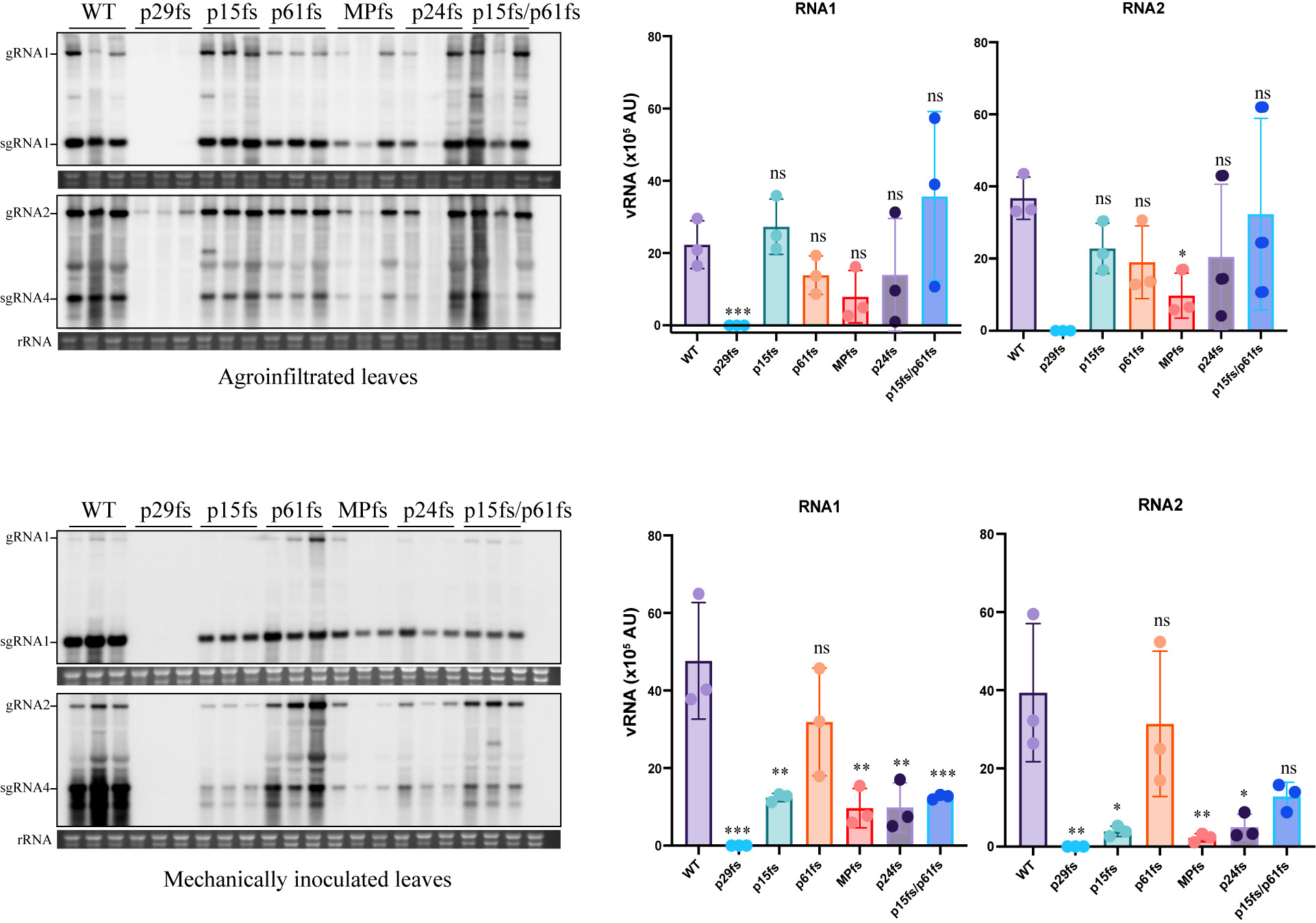

Supplement: S5 Fig — Leaves were agroinfiltrated with CiLV-C constructs carrying frameshift mutation in p29, p15, p61, MP, p24, and p15-p61. These infiltrated leaves were used as a source of inoculum for the mechanical inoculation of new plants, which were then analyzed for viral RNA accumulation. Northern blot (at 6 dpi) using DIG-probes complementary to the CiLV-C p29 and p24. The localization of CiLV-C gRNA1, sgRNA1, gRNA2, and sgRNA4 are indicated. rRNA stained with ethidium bromide indicates equal loading of samples. A negative control that corresponds to a non-infected plant is displayed in the final lane of the northern blots. RNA band intensity was quantified using Fuji software with the ISAC plugin and represented by the graphic. Graphics represent the relative accumulation (arbitrary units, AU) of CiLV-C RNAs. Data are the mean of three independent biological replicates. Error bars represent SD. An asterisk indicates a statistically significant difference according to unpaired Student’s t-test (two-tailed), *** p < 0.001, ** p < 0.01, * p < 0.05, and ns corresponds to p > 0.05. (TIF) [file ppat.1013388.s005.tif]

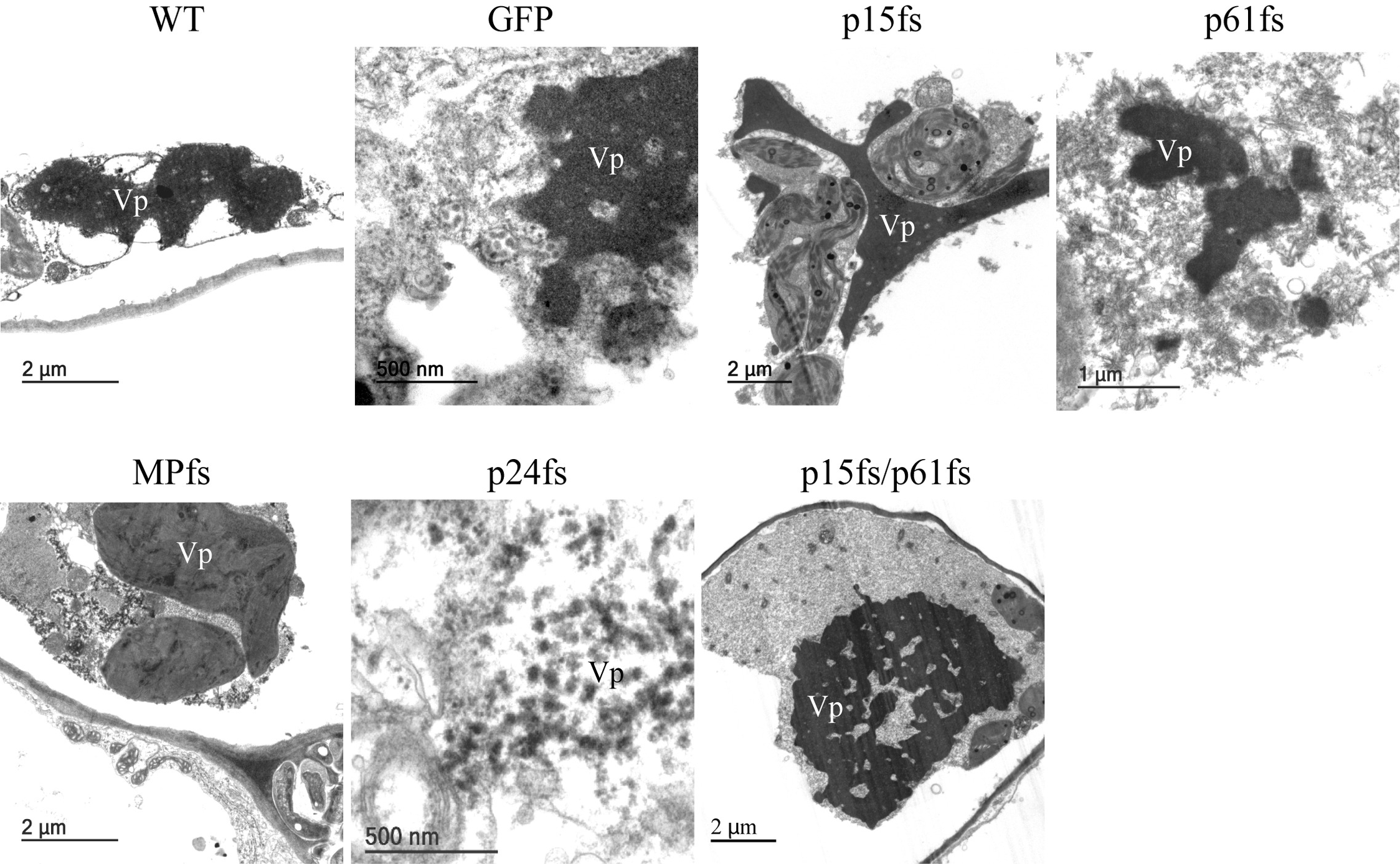

Supplement: S6 Fig — Structures similar to viroplasm-like (Vp) are indicated. Scale bars, 2 μm, 1 μm, and 500 nm. (TIF) [file ppat.1013388.s006.tif]

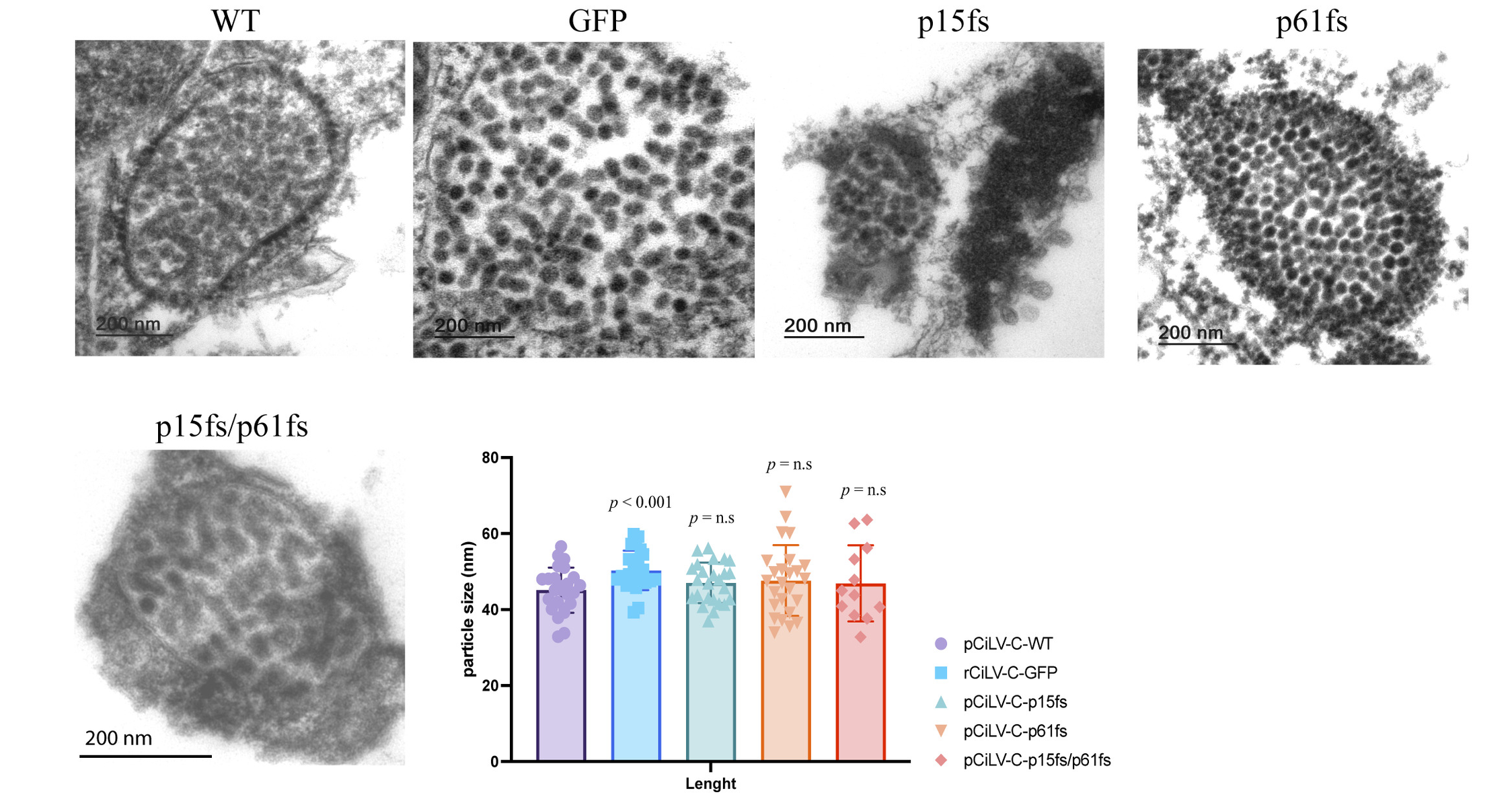

Supplement: S7 Fig — The length of virions was measured for wild-type CiLV-C (pCiLV-C-WT) and CiLV-C particles carrying frameshift mutations in p15 (pCiLV-C-p15fs), p61 (pCiLV-C-p61fs), and both p15-p61 (pCiLV-C-p15fs/p61fs), as well as for recombinant CiLV-C expressing the GFP (rCiLV-C-GFP). Electron micrographs of thin sections of N. benthamiana leaves infected with the respective constructs were analyzed. Mature enveloped virions were observed and measured. Bars represent the mean virion lengths, with sample sizes of n = 25 for all groups, except for p15fs/p61fs (n = 12). Error bars represent SD. p indicates a statistically significant difference according to unpaired Student’s t-test (two-tailed). n.s indicates no significant difference. p < 0.001 indicates a significant difference. (TIF) [file ppat.1013388.s007.tif]

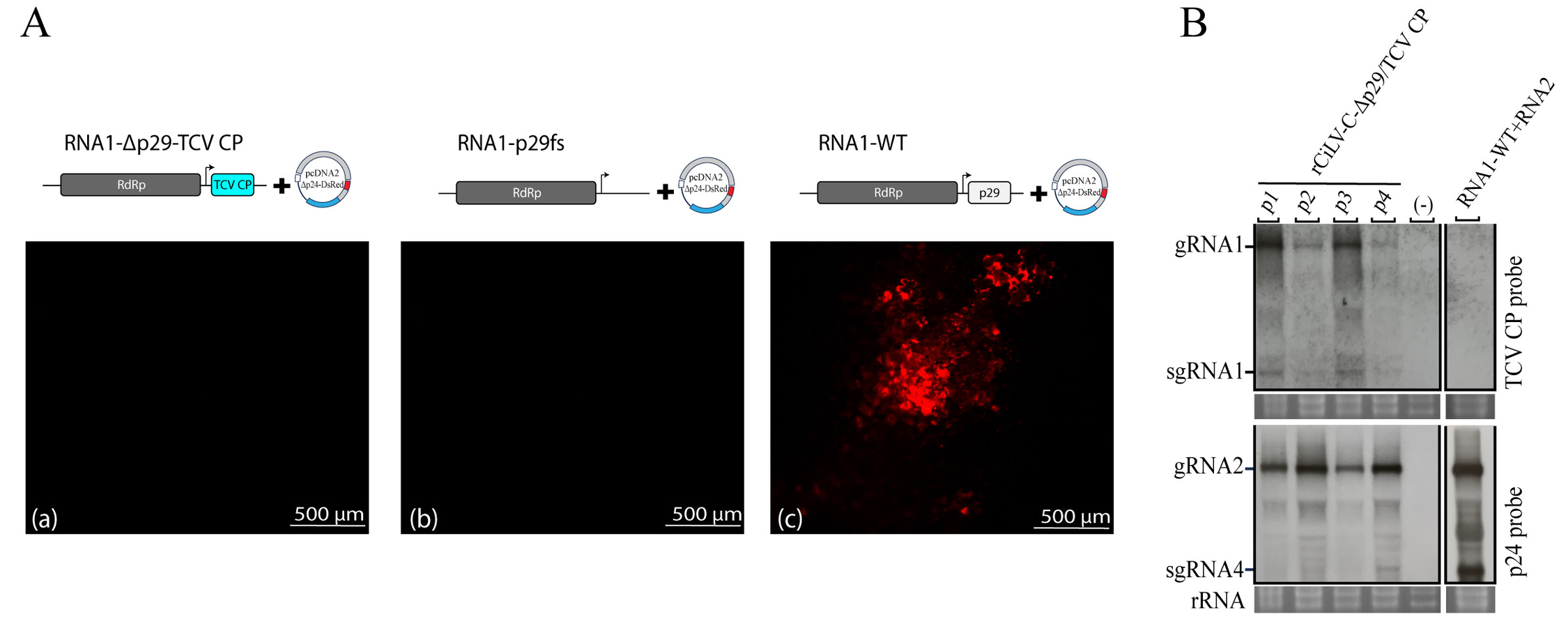

Supplement: S8 Fig — The p29 gene was replaced by the coat protein (CP) gene of turnip crinkle virus (TCV), generating the pcDNA1-Δp29/TCV CP construct. (A) N. benthamiana leaves were co-infiltrated with pcDNA1-Δp29/TCV CP + pcDNA2-Δp24/DsRed (a construct in which the p24 gene was replaced by DsRed) (a), pcDNA1-p29fs + pcDNA2-Δp24/DsRed (b), and pcDNA1-WT + pcDNA2-Δp24/DsRed (c). RFP signal was monitored for up to 15 dpi, and images were taken at 10 dpi. Bars correspond to 500 μm. (B) Northern blot analysis of RNA extracted from leaves of four plants (p1 to p4) agroinfiltrated with rCiLV-C-Δp29/TCV CP (pcDNA1-Δp29/TCV CP + pcDNA2) and pCiLV-C-WT (pcDNA1-WT + pcDNA2). DIG-probes complementary to the TCV CP and CiLV-C p24 genes were used. (-) corresponds to a non-infected plant. Ethidium bromide staining of rRNA indicates equal loading of samples. The localization of CiLV-C gRNA1, gRNA2, sgRNA1, and sgRNA4 is indicated. The large membrane piece (on the left) containing the rCiLV-C-Δp29/TCV CP samples was highly exposed to visualize the bands. (TIF) [file ppat.1013388.s008.tif]

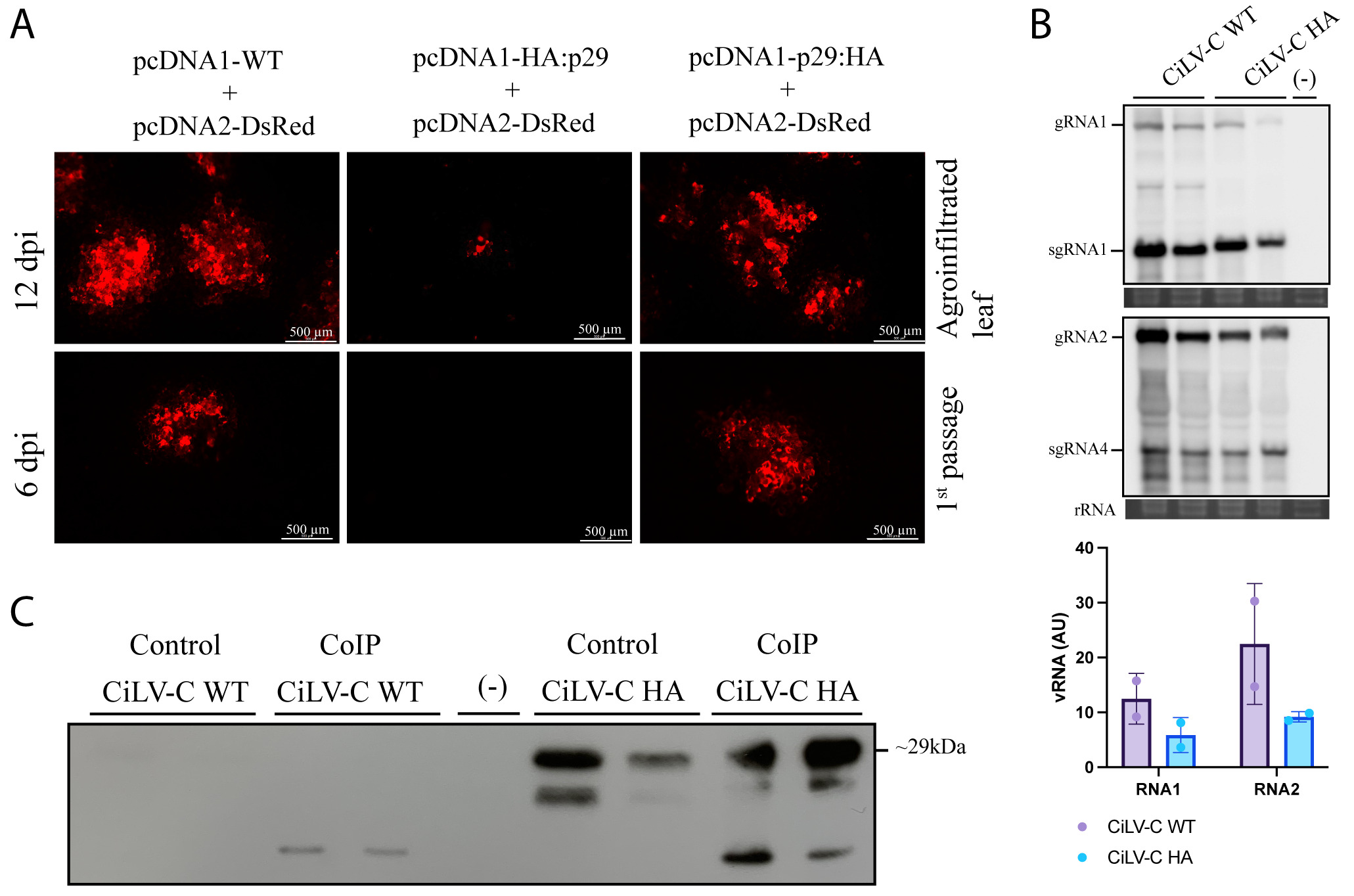

Supplement: S9 Fig — (A) Analysis of cell-to-cell movement of pcDNA1-WT, and recombinant versions carrying HA-Tag fused to the N- or C-terminus of the p29 (pcDNA1-HA:p29 and pcDNA1-p29:HA, respectively). DsRed fluorescence was captured at 12 dpi. N. benthamiana leaves were mechanically inoculated (1st pass) with sap extract from infiltrated leaves, and cell-to-cell movement was monitored at 6 dpi. (B) Northern blot analysis of CiLV-C RNAs 1 and 2 accumulation at 6 dpi, from N. benthamiana leaves agroinfiltrated with pcDNA1-WT + pcDNA2 (CiLV-C WT) and pcDNA1-p29:HA + pcDNA2 (CiLV-C HA) using a DIG-probe complementary to the CiLV-C p29 and p24 genes. The negative control (-) corresponds to a non-infected plant. Ethidium bromide staining of rRNA indicates equal loading of samples. The localization of CiLV-C gRNA1, gRNA2, sgRNA1, and sgRNA4 is indicated. The graphs show the mean relative accumulation (AU) of total CiLV-C RNAs from two independent biological replicates. (C) Immunoprecipitation and western blot analysis of CiLV-C p29:HA. Immunoprecipitation of CiLV-C p29 was analyzed by western blot using an anti-HA antibody. The results show the accumulation of the p29:HA protein obtained from tissue inoculated with the unlabeled version (pcDNA1-WT + pcDNA2, CiLV-C WT) and the HA-tagged version (pcDNA1-p29:HA + pcDNA2, CiLV-C HA), before (control) and after (CoIP) immunoprecipitation. The protein size, expressed in kDa, is shown on the right side of the image. (TIF) [file ppat.1013388.s009.tif]

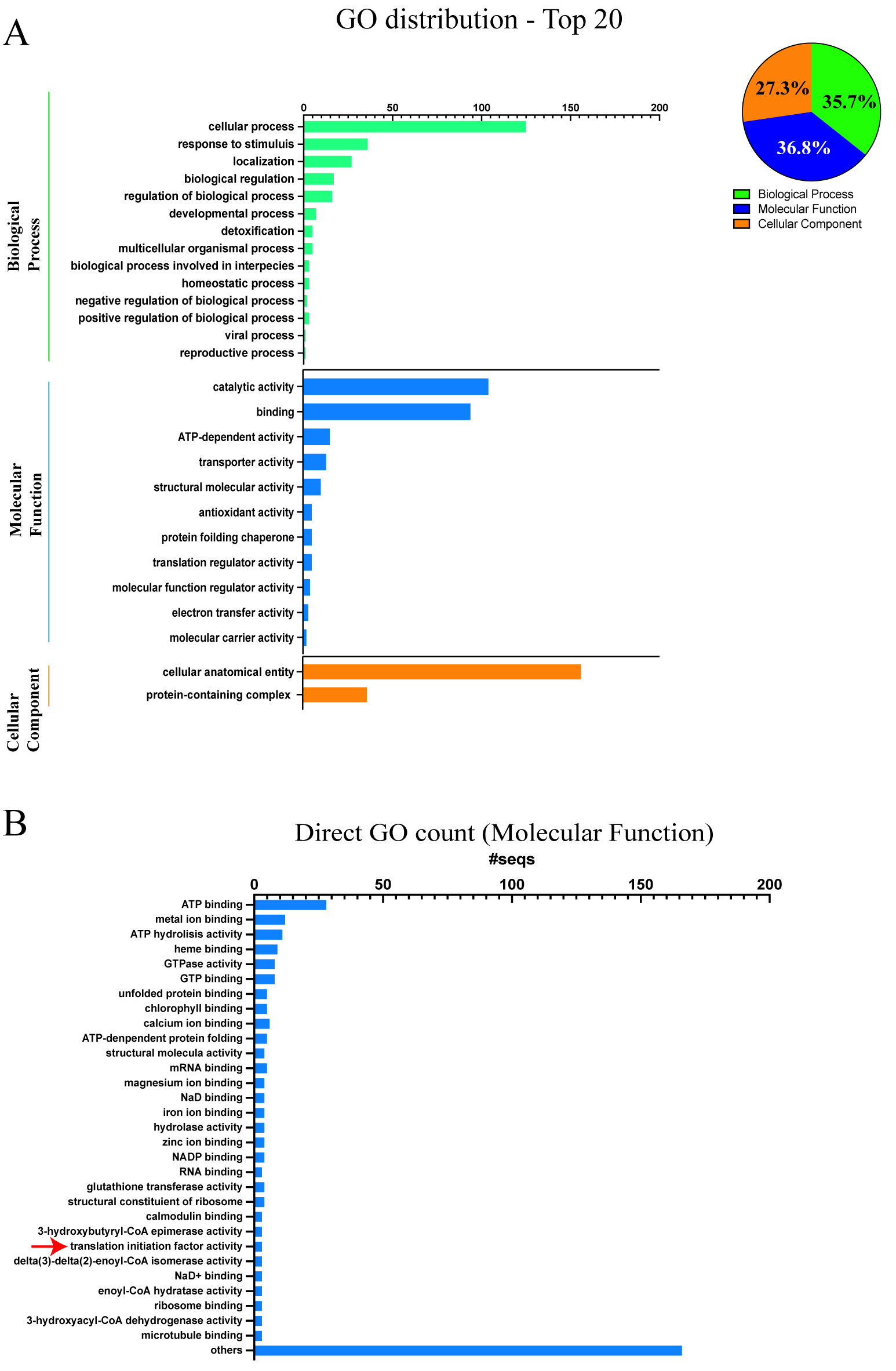

Supplement: S10 Fig — (A) Ontological distribution. Graphs corresponding to the three ontological categories: biological process, molecular function, and cellular component, are shown. Each graph displays the top 20 subcategories into which the analyzed protein set was classified. The proportion of proteins assigned to each category is indicated in the pie chart. Note that a single protein can be assigned to multiple subcategories. (B) Ontological count of molecular function. The graph illustrates the top 30 subcategories assigned into the molecular function. The red arrow highlights the translation initiation factor subcategory. The ontological analyses were generated using the Omicsbox 3.2.2 software. (TIF) [file ppat.1013388.s010.tif]

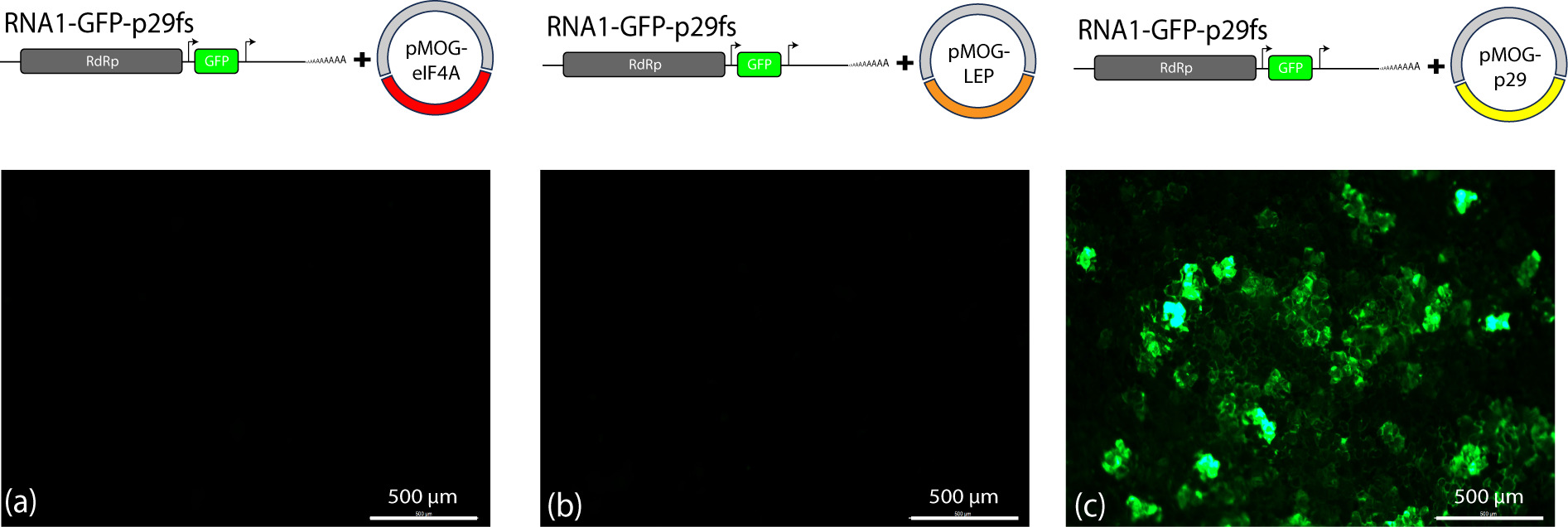

Supplement: S11 Fig — (A) In vivo interaction between the p29 and eIF4E/PABP. PABP and eIF4E were targeted at their C-terminus with NYFP or CYFP, respectively, and co-expressed with p29 fused at its N- or C-terminus with the NYFP or CYFP. A representative protein pair combination is indicated at the top of the image. The image shows the reconstitution of the YFP fluorescence distributed through the cell cytoplasm of N. benthamiana cells. The image is representative of several infiltrated leaves from three different plants. The fluorescent signals were captured at 3 dpi. The green (GFP), transmitted light (T.L) channels, and merged images are shown in the figure. The negative control is illustrated by a representative image (mock) showing the expression of eIF4A, eIF4E, and PABP combined with Ncyt constructs and p29 combined with the Cer construct. The bars indicate 50 μm. (B) CoIP of CiLV-C p29 with eIF4E and PABP. Extracts of N. benthamiana leaves expressing p29 fused at its N- or C-terminus with an HA epitope and 3xMyc targeted eIF4E or PAPB at their N- or C-terminus, were analyzed at 3dpi. The CoIP assay was addressed using the Pierce HA-Tag Magnetic IP/CoIP Kit. Western blot analysis was carried out using Myc and HA antibodies. C + , positive control (non-immunoprecipitated samples); IP, immunoprecipitated samples. ‘+’ and ‘–‘ signs indicate the presence or absence of the corresponding proteins in the leaf extracts. (TIF) [file ppat.1013388.s011.tif]

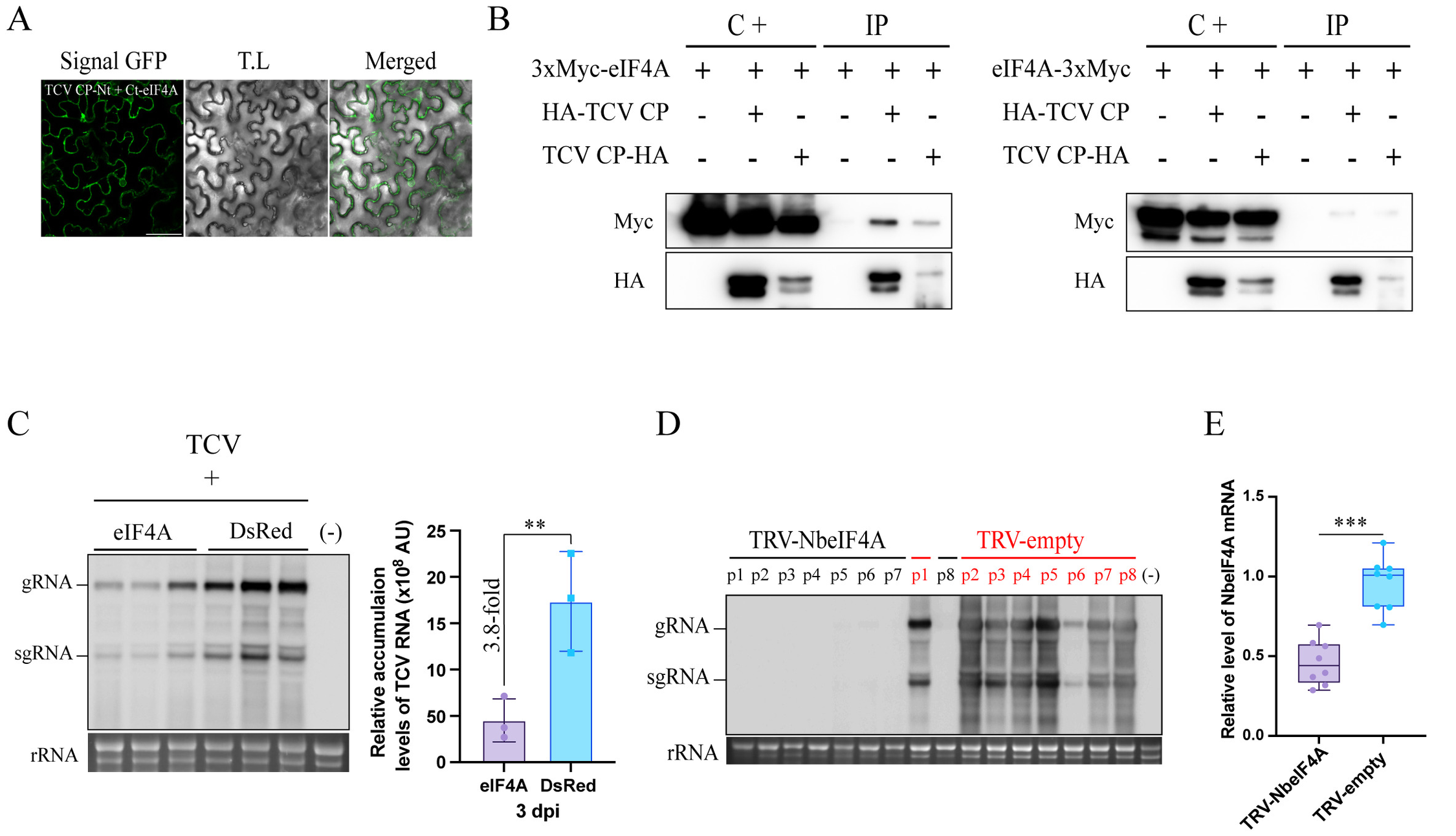

Supplement: S13 Fig — (A) In vivo interaction between the TCV CP and eIF4A. The eIF4A was targeted at its C-terminus with NYFP or CYFP and co-expressed with the TCV CP fused at either its N- or C-terminus with the NYFP or CYFP. A representative protein pair combination is indicated at the top of the image. The image shows the reconstitution of the YFP fluorescence into the nuclei and distributed through the cell cytoplasm of N. benthamiana leaves. The image is representative of several infiltrated leaves from three different plants. The fluorescent signals were captured at 3 dpi. The green (GFP), transmitted light (T.L) channels, and merged images are shown in the figure. The bars indicate 50 μm. (B) CoIP of TCV CP with eIF4A. Extracts of N. benthamiana leaves expressing the TCV CP fused at either the N- or C-terminus with an HA epitope, along with eIF4A tagged at the N- or C-terminus with 3 × Myc, were analyzed at 3dpi. The CoIP assay was addressed using the Pierce HA-Tag Magnetic IP/CoIP Kit. Myc and HA antibodies were used in the western blots. C + , positive control (non-immunoprecipitated samples); IP, immunoprecipitated samples. “+” and “–” signs indicate the presence or absence of the corresponding proteins in the leaf extracts. (C) Left panel: northern blot analysis (3 dpi) showing the levels of TCV genomic (g) and subgenomic (sg) RNAs in plants overexpressing eIF4A or DsRed (control) infected with TCV. RNA detection was performed using a DIG-probe complementary to the TCV CP gene. The localization of TCV gRNA and sgRNA is indicated. Ethidium bromide-stained rRNA serves as a loading control. Right panel: graph showing the mean relative accumulation levels of TCV RNA from plants expressing eIF4A or DsRed. Error bars represent SD. Asterisks indicate statistically significant differences according to unpaired Student’s t-test (two-tailed), ** p < 0.01. (D) Northern blot analysis of TCV RNA accumulation in plants control (TRV-empty) and eIF4A-silenced (TRV-NbeIF4A) infected with TCV [file ppat.1013388.s013.tif]

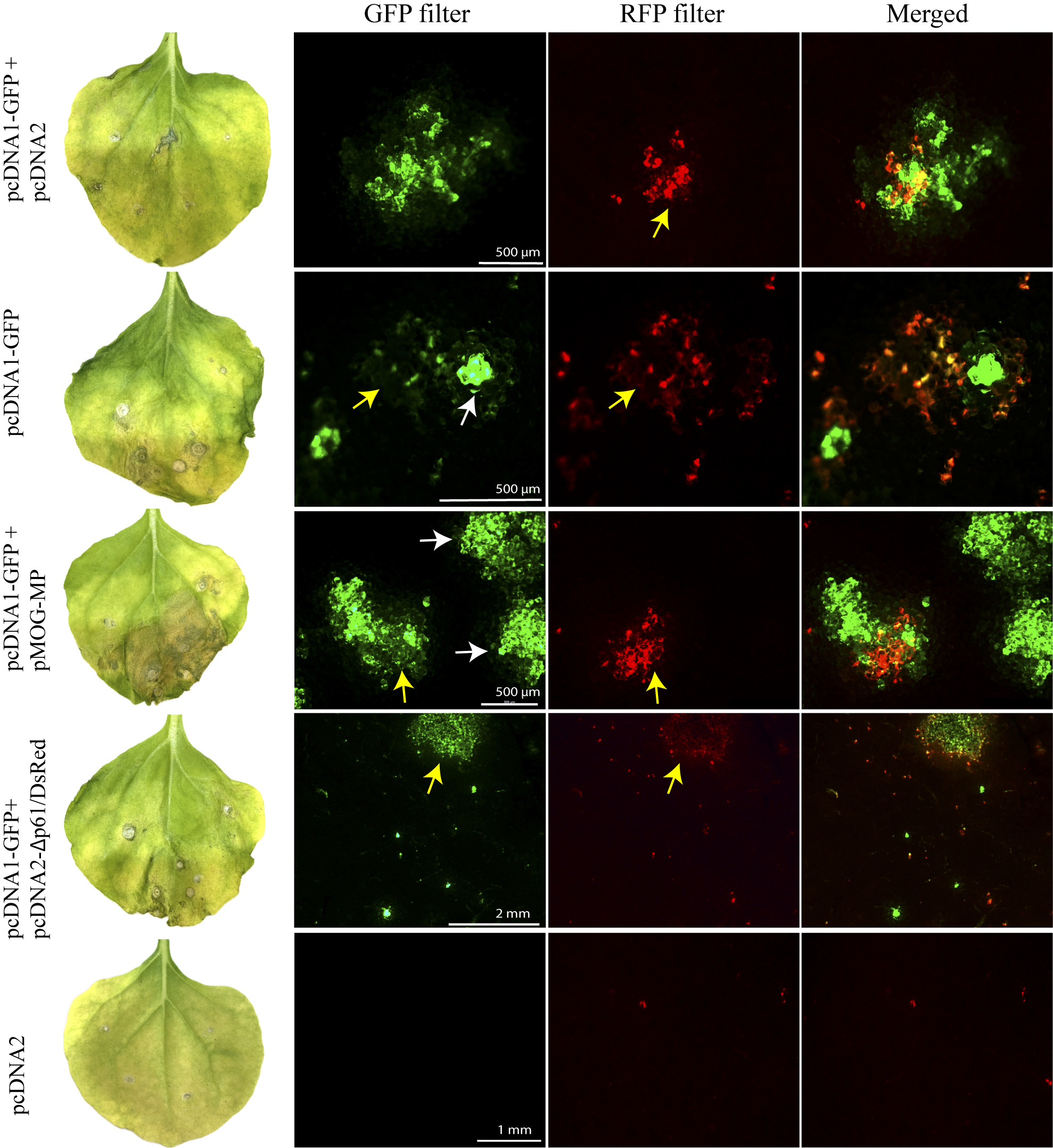

Supplement: S14 Fig — N. benthamiana leaves agroinfiltrated with pcDNA1-GFP + pcDNA2, pcDNA1-GFP, pcDNA1-GFP + pMOG-MP, pcDNA1-GFP + pcDNA2-Δp61/DsRed, and pcDNA2 at 15 dpi. To determine whether RNA1 plays a direct role in the necrotic lesions, we infiltrated leaves with Agrobacterium cultures transformed only pcDNA1-GFP and bacteria combination carrying pcDNA1-GFP + pMOG-MP, which favors increased accumulation of RNA1 due to increased dissemination. To determine whether p61 (both the protein and gene) plays a direct role in the necrotic lesions, leaves were infiltrated with rCiLV-C-Δp61/DsRed, which the p61 sequence was replaced by DsRed. Necrotic reaction distributed through the infiltrated tissue is exhibited in all constructs, except in leaves infiltrated with only pcDNA2, indicating that RNA1 is enough to trigger the necrosis response and that p61 is not directly associated with this phenotype. Leaves under UV light reveal necrosis reaction in zones infected by rCiLV-C constructs. Intense autofluorescence under RFP filter indicates necrosis regions. Yellow arrows show autofluorescence signal, while white arrows show GFP fluorescence signal from CiLV-C infection. Fluorescence loupe images show the transmitted light (TL), GFP filter, RFP filter, and merged images. Scale bars correspond to 500 μm, 1 mm, and 2 mm. (TIF) [file ppat.1013388.s014.tif]

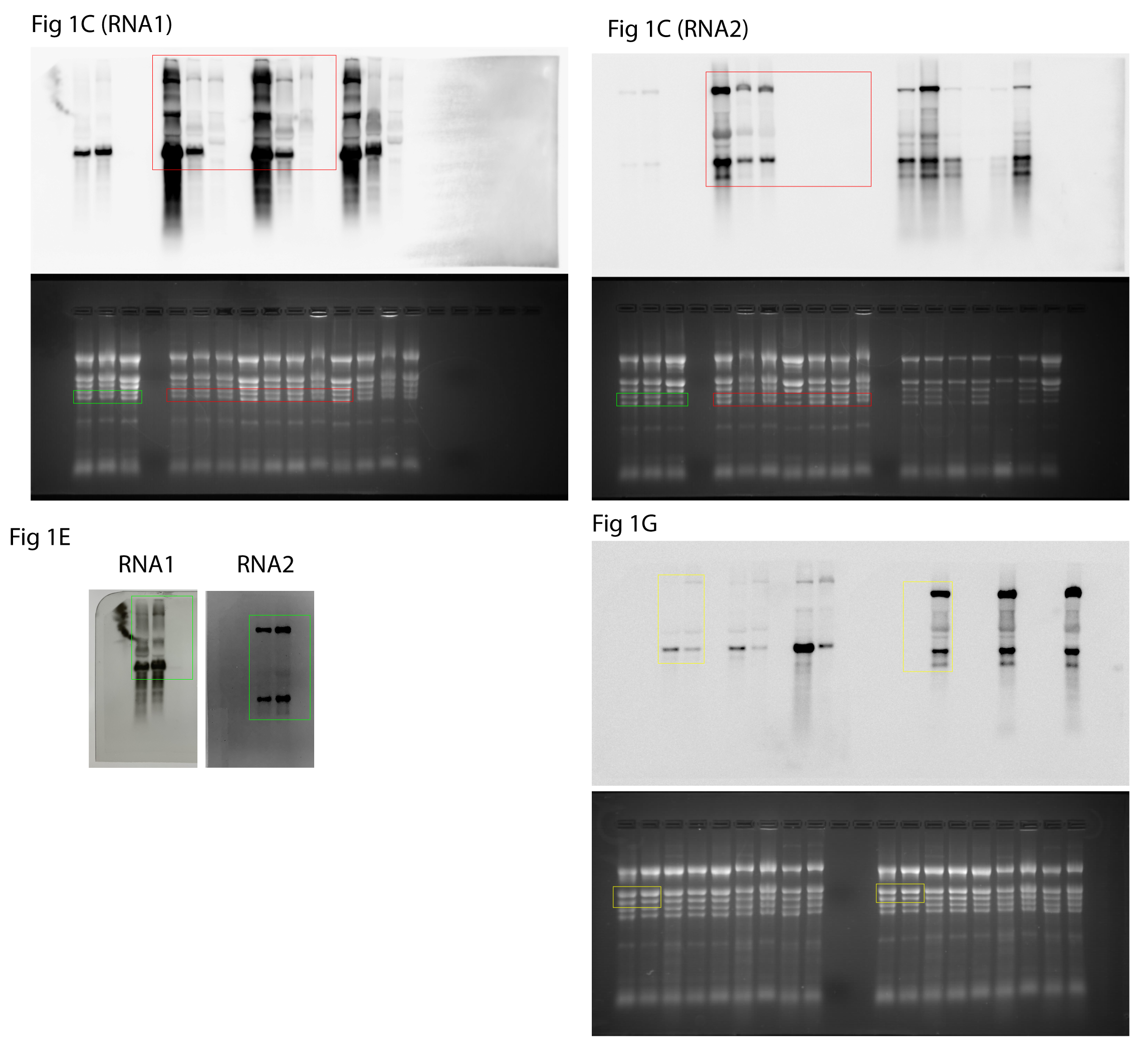


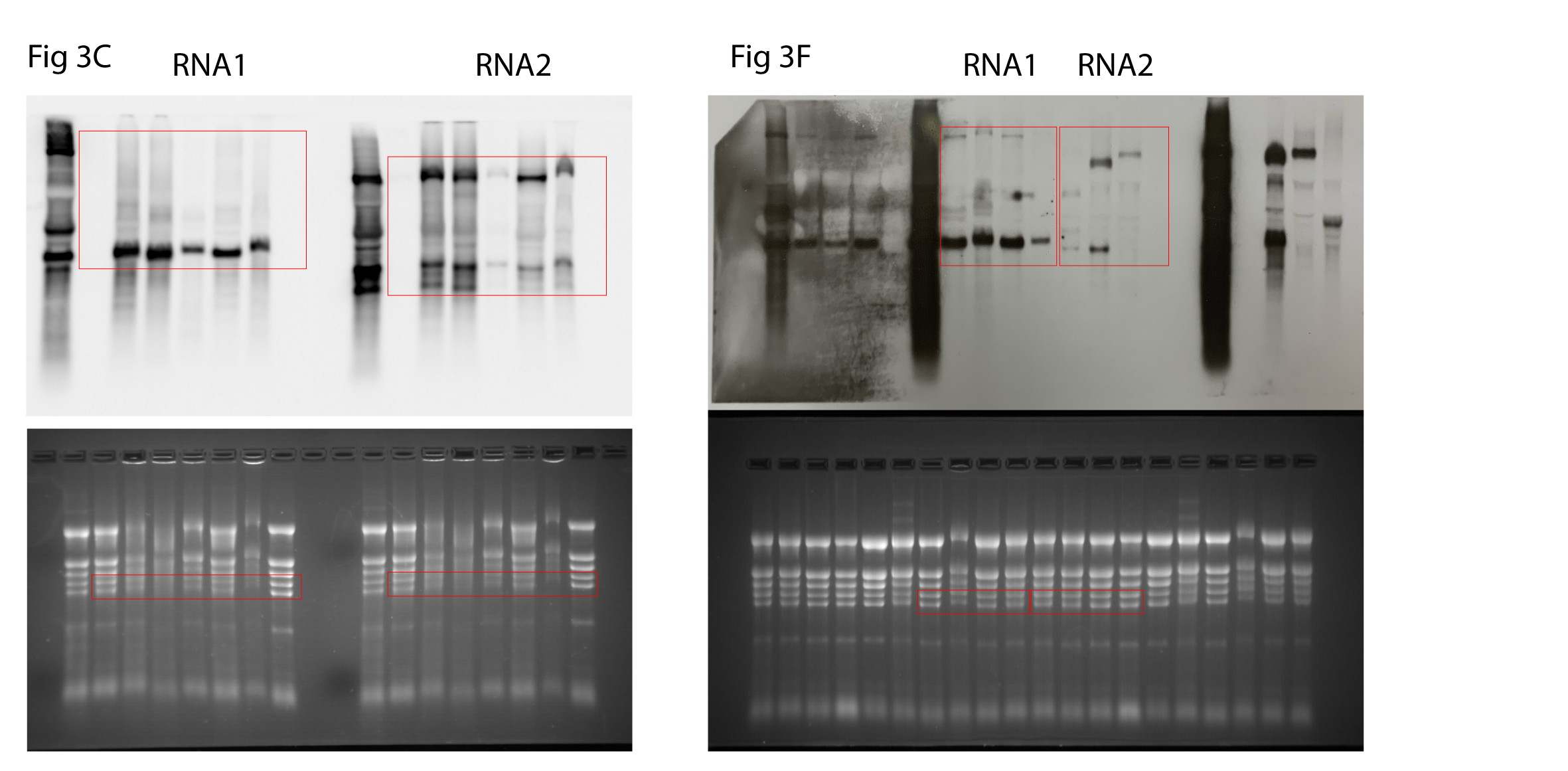


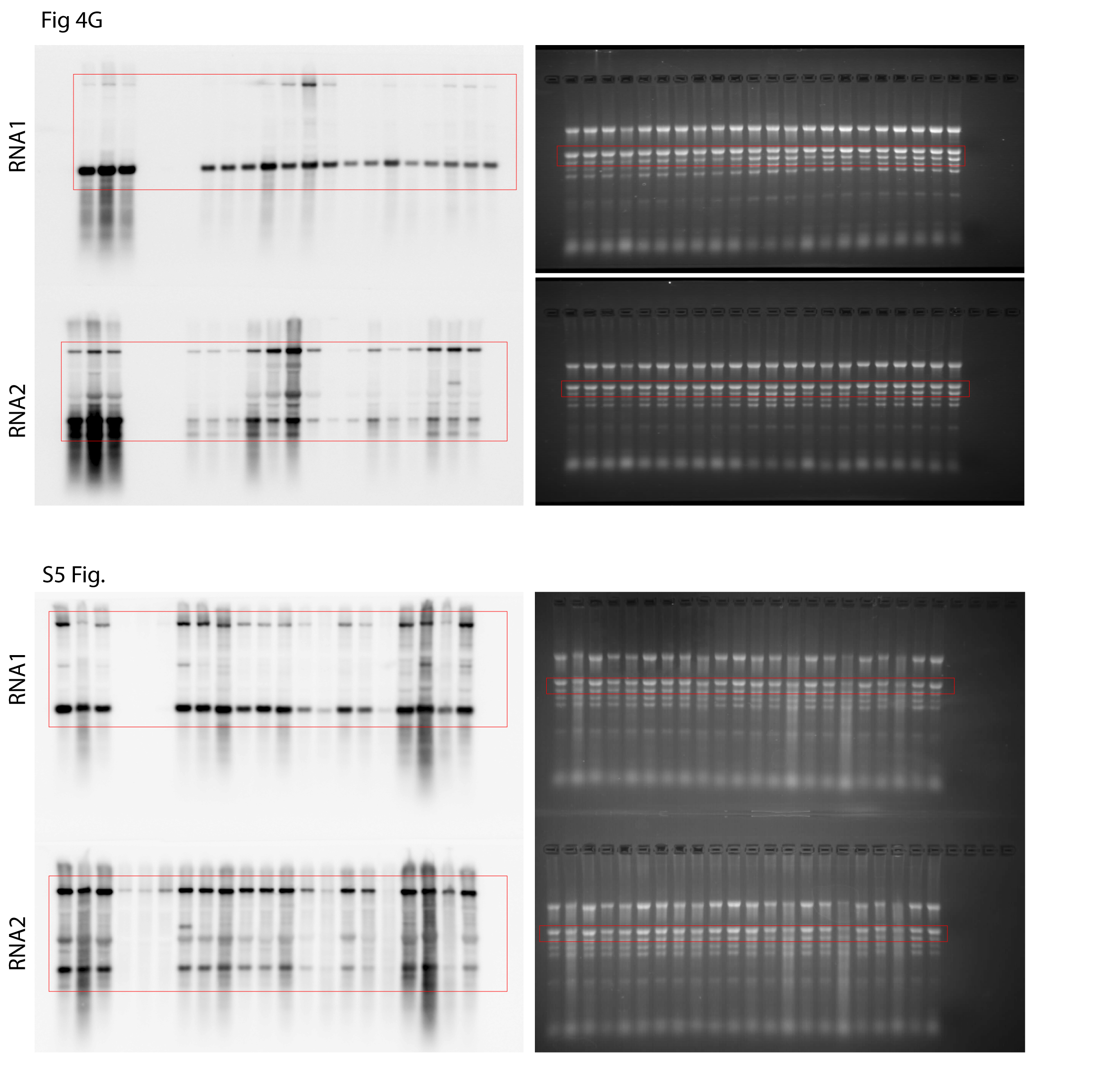


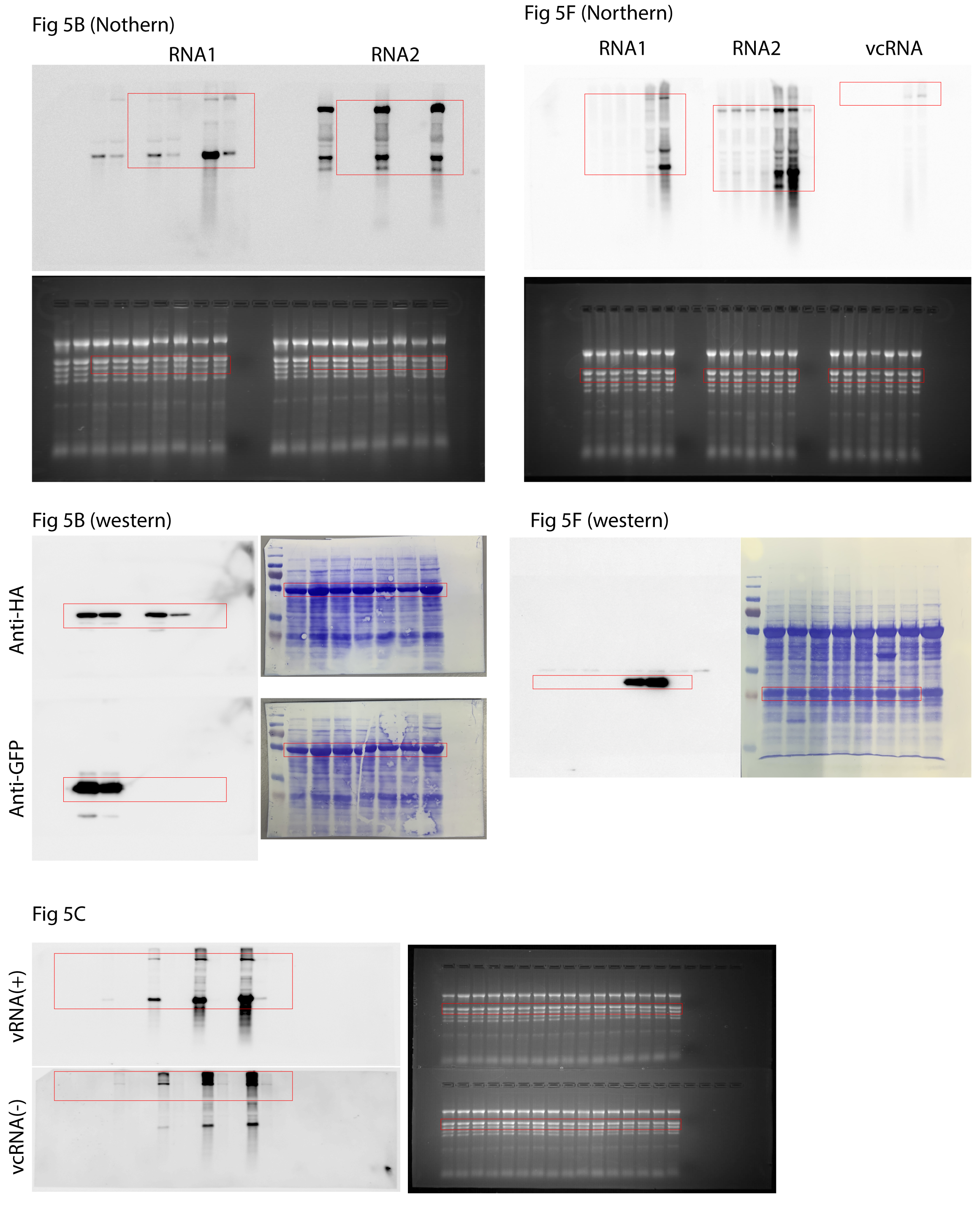


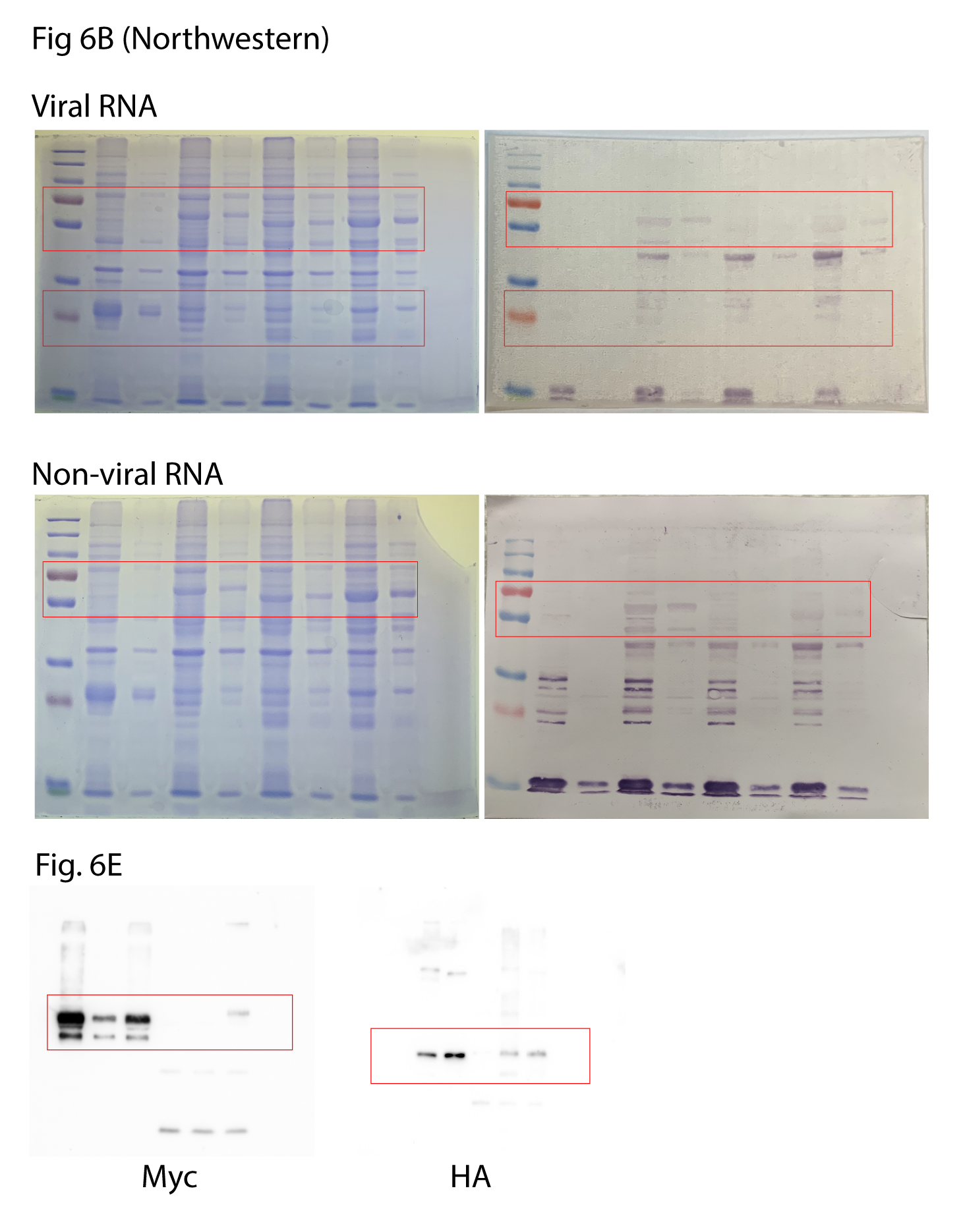


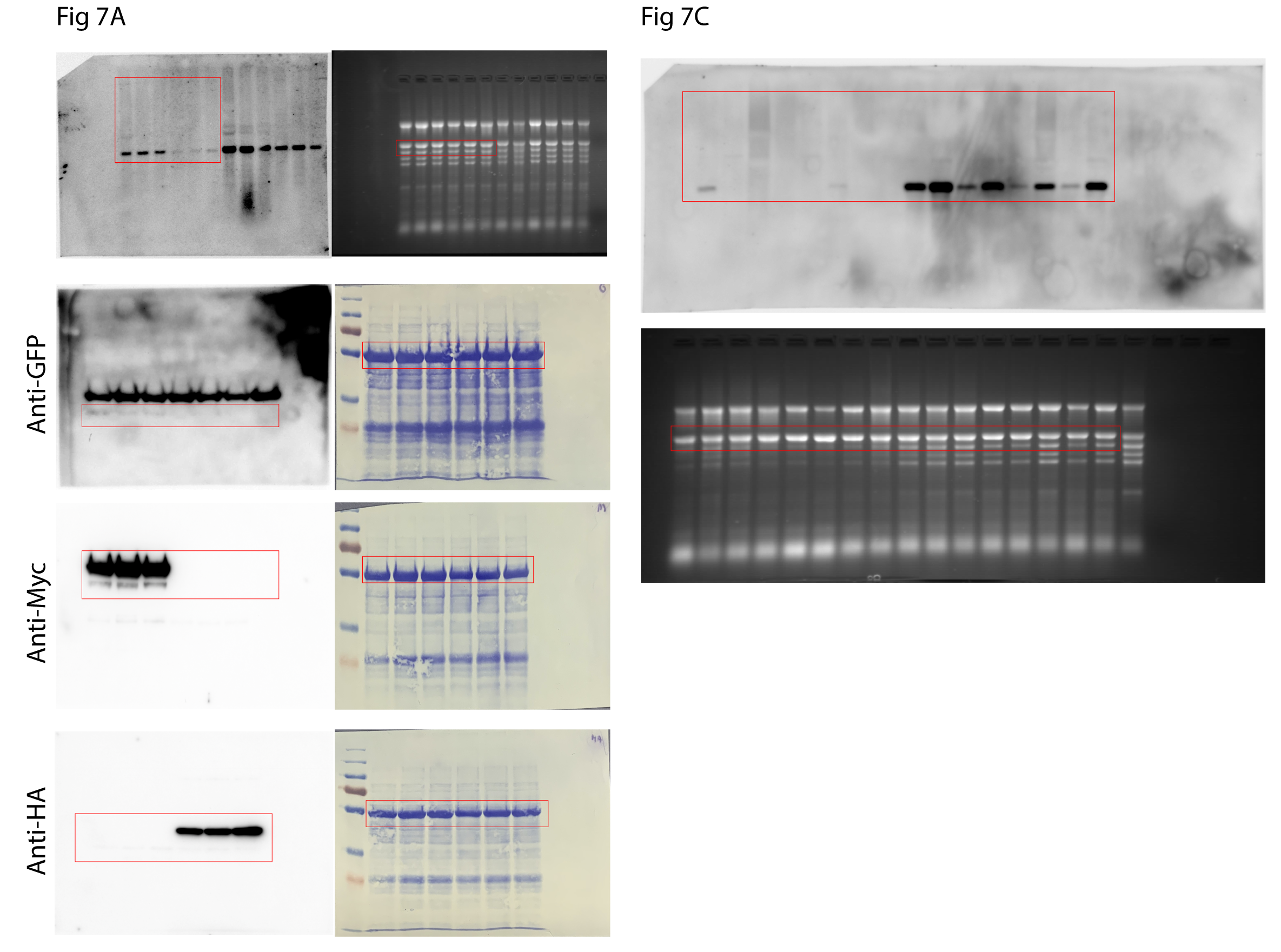


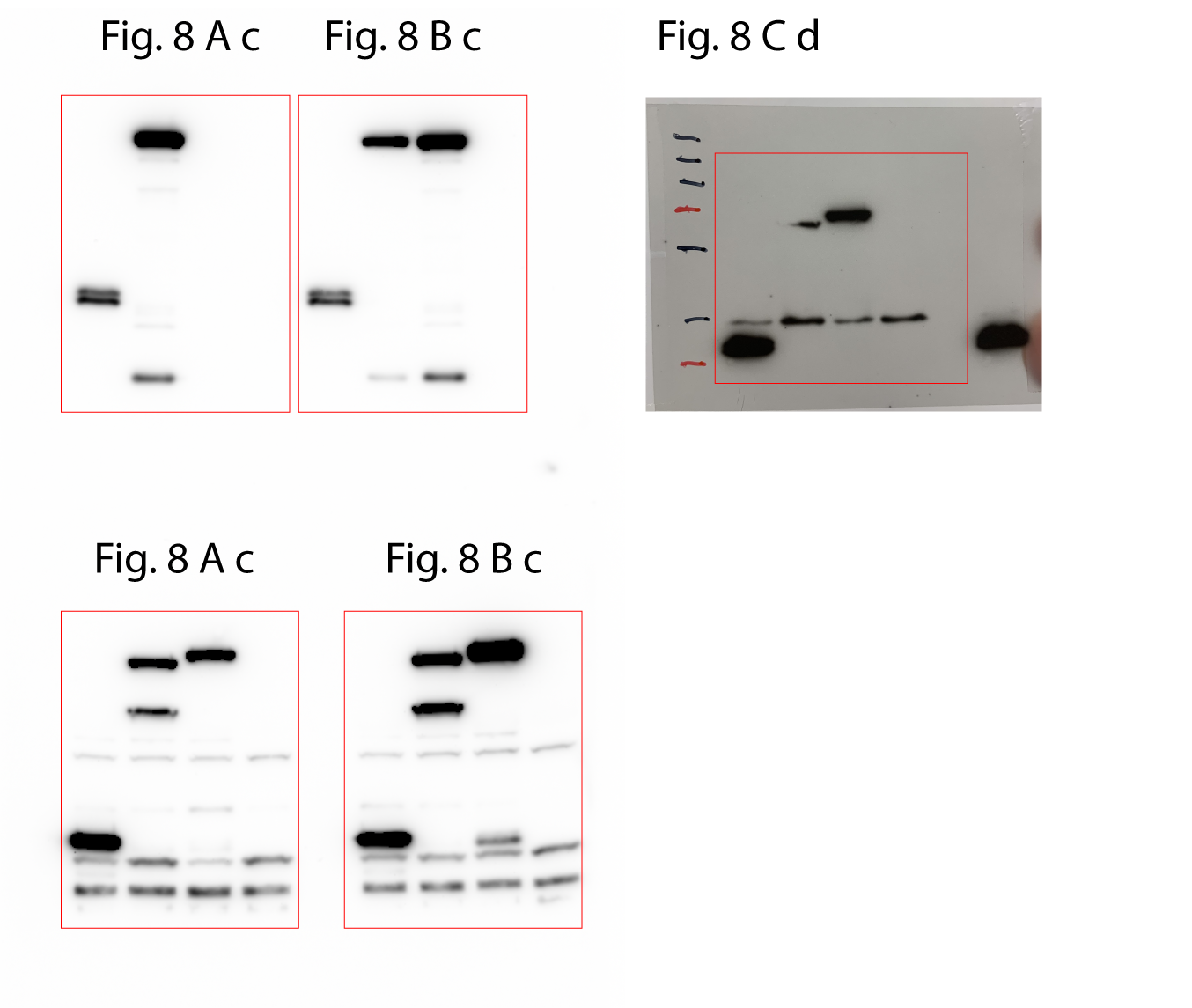


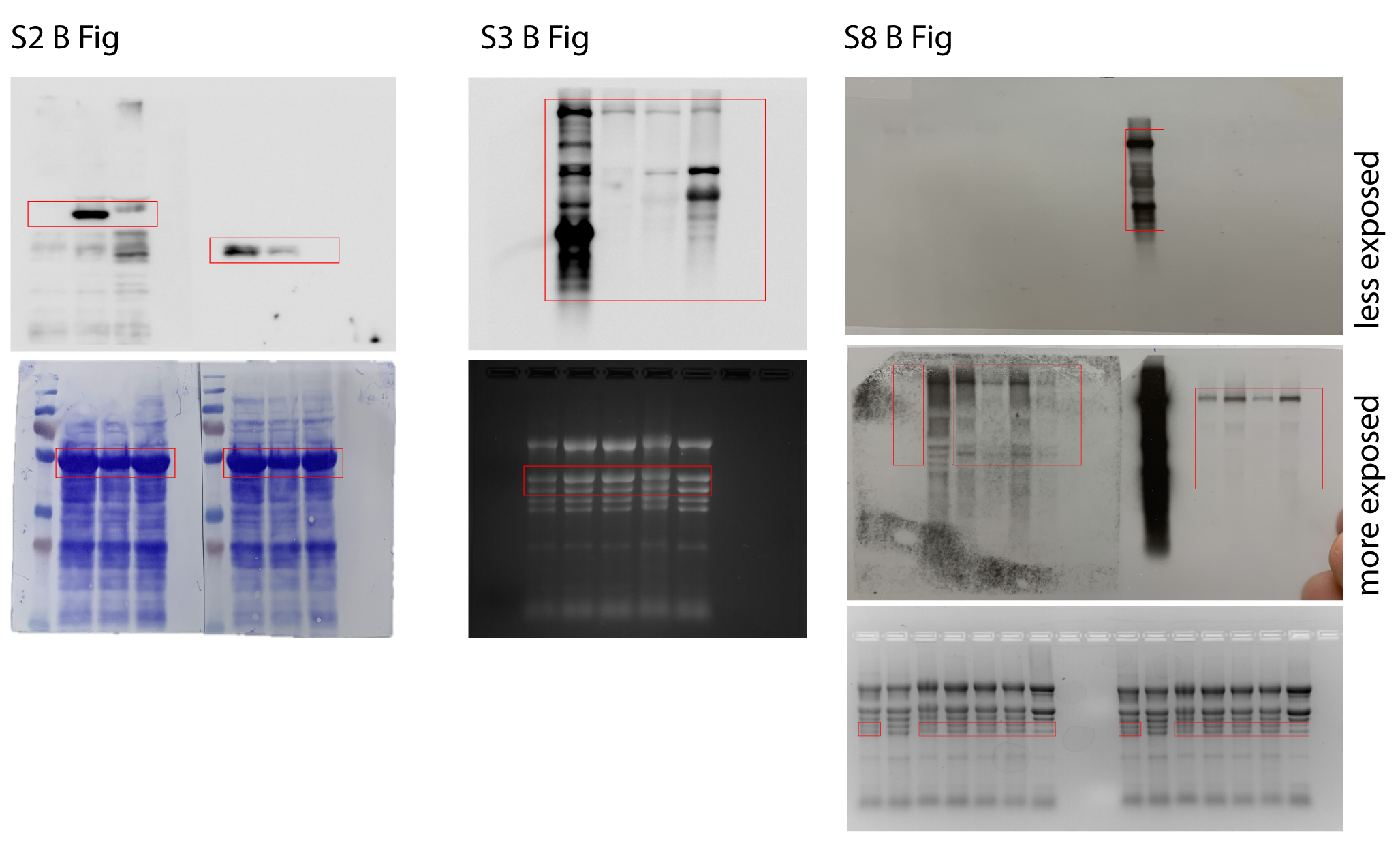


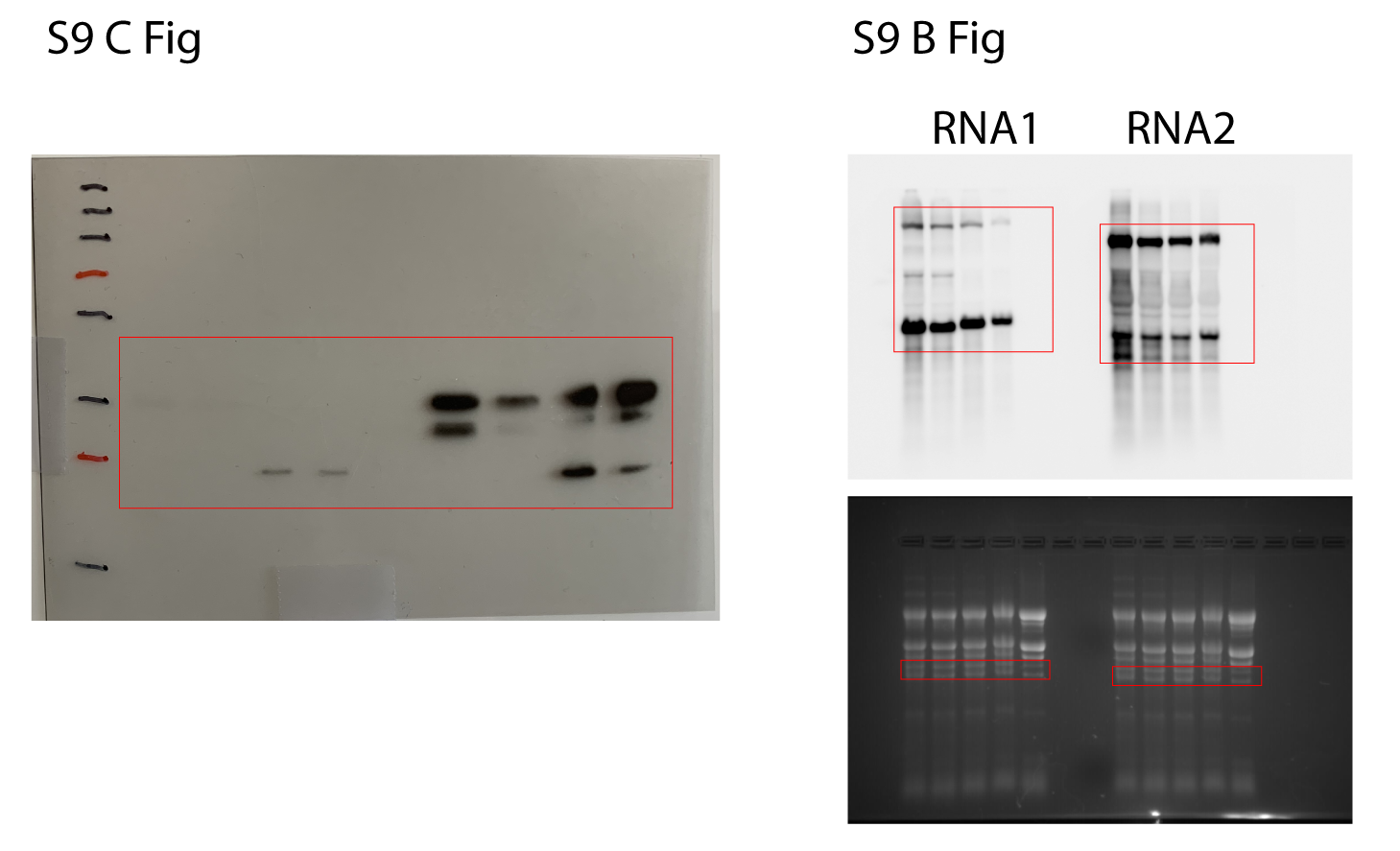


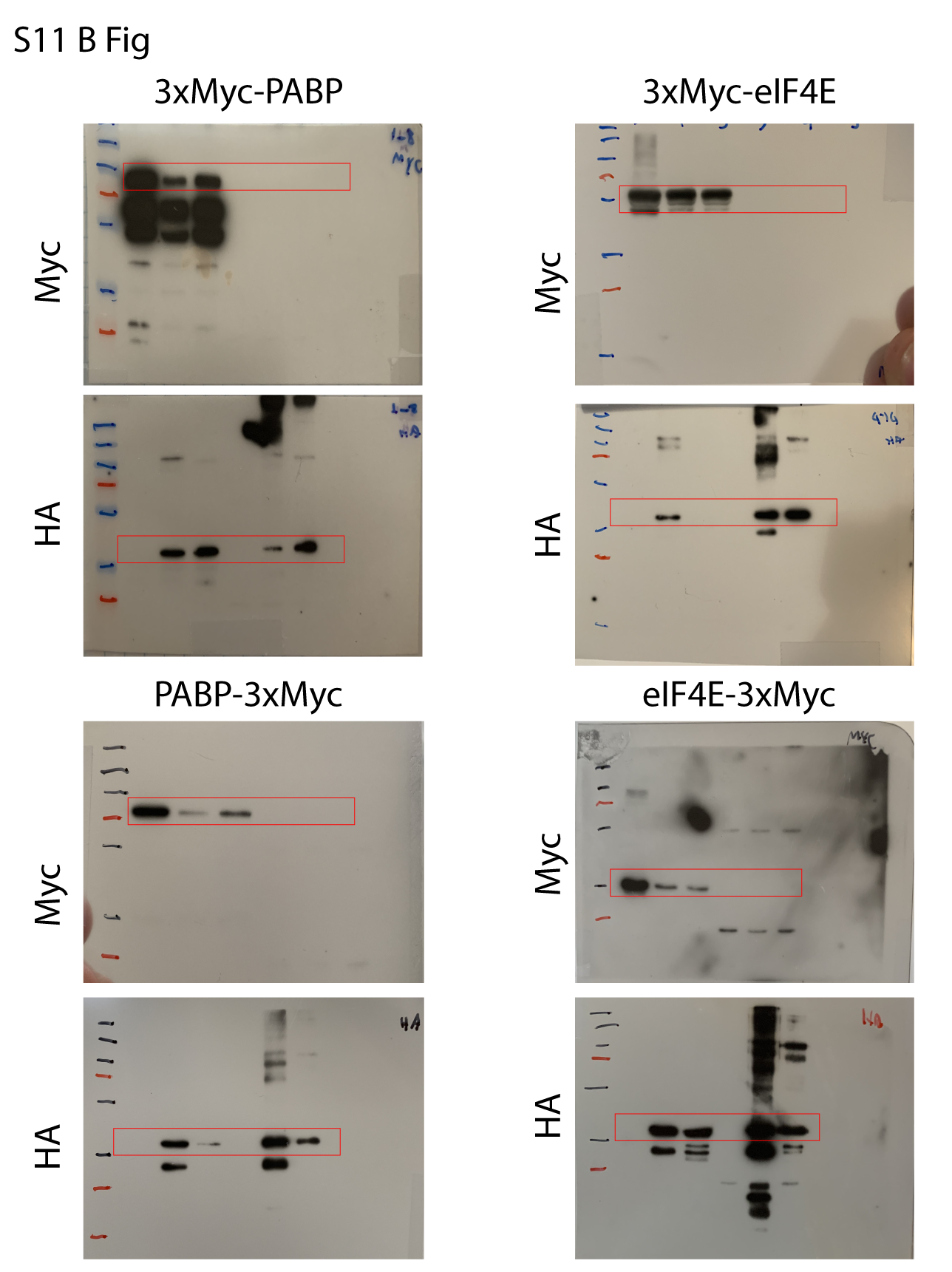


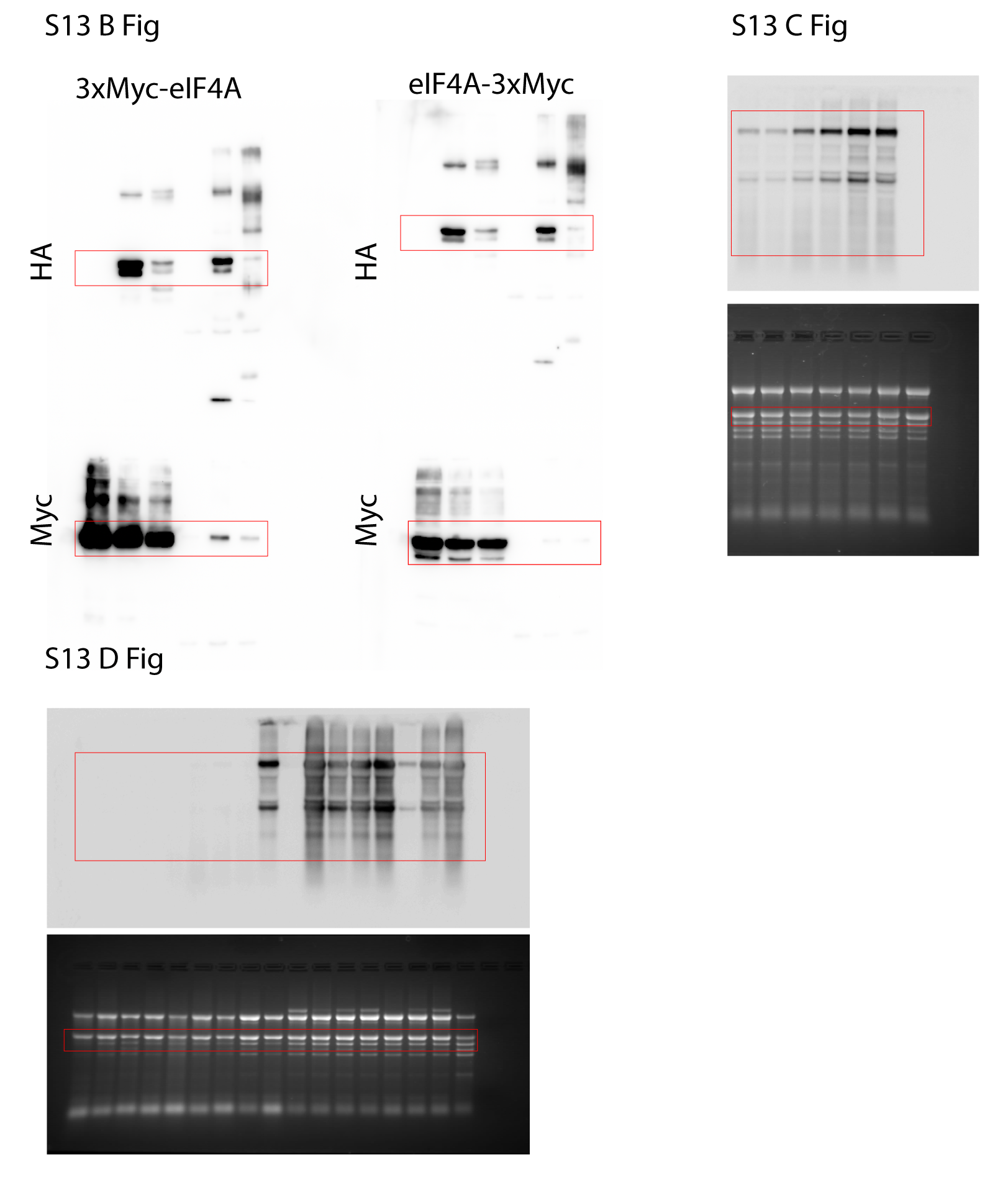

Supplement: S2 Data — (DOCX) [file ppat.1013388.s018.docx]
