# Supplementary material for: From darkness to light: Genetic manipulation of an atypical plant virus unveils key insights into kitavirus biology, highlighting capsid protein and eIF4A engagement to drive viral infection
Source: PLoS Pathog. 2025 Aug 1;21(8):e1013388. doi: 10.1371/journal.ppat.1013388 (PMC12334043; doi:10.1371/journal.ppat.1013388)
Supplement: S2 Table — (DOCX) [file ppat.1013388.s016.docx]

Mikhail Oliveira Leastro ^1^*, Elliot Watanabe Kitajima ^2^, Vicente Pallás ^1^, Jesús Ángel Sánchez-Navarro ^1^*

^1^ Department of Stress Biology, Institute of Molecular and Cellular Biology of Plants, CSIC- Universitat Politècnica de València. Valencia, Spain;

^2^ Department of Phytopathology and Nematology, University of Sao Paulo, Luiz de Queiroz College of Agriculture. Piracicaba, Brazil.

**From darkness to light: Genetic manipulation of an atypical plant virus unveils key insights into kitavirus biology, highlighting capsid protein and eIF4A engagement to drive viral infection**

S2 Table. Primers used in this study

| **Name** | **Sequence 5'- 3'** | **Target** | **Description** |
| --- | --- | --- | --- |
| cDNA1 Fwd | ATGAGTATCGTAACTTTCACTTTGACTGACC | pJL89 cDNA1 | 5’ end of the p29 gene |
| cDNA1 Rev | AATGACAAAATACAAATTCGCGGTAA | pJL89 cDNA1 | 3’ end of the gene junction (GJ) |
| EGFP Fwd | ATGGTGAGCAAGGGCGAG | EGFP | 3’ end of the gene junction (GJ) |
| EGFP:GJ 31pb Rev | CAATAATAGTATCATAAAAGTCAGTATTACTTTACTTGTACAGCTCGTCC | EGFP | 3' end of the EGFP |
| EGFP:GJ 62pb Rev | AATGACAAAATACAAATTCGCGGTAAAATATCAATAATAGTATCATAAAAG | EGFP:GJ 31pb | 3' end of EGFP carrying 31pb of GJ |
| EGFP 25pb Fwd | TACCGCGAATTTGTATTTTGTCATTATGGTGAGCAAGGGCGAG | EGFP:GJ 62pb | 5' end of the EGFP:GJ 62pb |
| EGFP 25pb Rev | TCAAAGTGAAAGTTACGATACTCATAATGACAAAATACAAATTCGCG | EGFP:GJ 62pb | 3' end of the EGFP:GJ 62pb |
| cDNA1 Fwd NheI | TATAGCTAGCGTCGGTGGATTAATGATGGGGGT | pJL89 cDNA1 | 3’ end of the p29 gene |
| cDNA1 Rev NheI | TATAGCTAGCCTACTGCGCTGAGTCGGA | pJL89 cDNA1 | RNA1 3 UTR |
| Poly+link2xser Rev | TCTAGCTTAATTAATGACAGGAAGCAAATTACCCTC | pJL 89 cDNA1-GFP | 3’ end of the polymerase |
| EGFP Fwd | ATGGTGAGCAAGGGCGAGGA | pJL 89 cDNA1-GFP | 5’ end of the p29 gene |
| EGFP:p29 Fwd | ATGAGTATCGTAACTTTCACTTTGAC | pcDNA1-GFP-29 | 5' end of the p29 gene |
| EGFP:p29 Rev | CTTGTACAGCTCGTCCATGC | pcDNA1-GFP-29 | 3' end of the EGFP gene |
| p29:EGFP Fwd | ATGGTGAGCAAGGGCGAGGAG | pcDNA1-p29-GFP | 5' end of the EGFP gene |
| p29:EGFP Rev | CTGCGCTGAGTCGGAGTCA | pcDNA1-p29-GFP | 3' end of the p29 gene |
| p29(fs) Fwd | ATGTATCGTAACTTTCACTTTGACTG | pJL89 cDNA1 and cDNA1-GFP | 5’ end of the p29 gene |
| p29(fs) Rev | AATGACAAAATACAAATTCGGTAAAATATC | pJL89 cDNA1 and cDNA1-GFP | 3’ end of the gene junction (GJ) |
| p15(fs) Fwd | ATGAAACTGGTCTACGATTGAGTGG | pJL89 cDNA2 | 5’ end of the p15 gene |
| p15(fs) Rev | TGTAATGTTCACTAATAATTGAGAGTTATTAGCC | pJL89 cDNA2 | 3’ end of the RNA2 5' UTR |
| p61(fs) Fwd | ATGGCGATTTCAGCTTTTTAGTTTC | pJL89 cDNA2 | 5’ end of the p61 gene |
| p61(fs) Rev | TTGACAATACTATTTTAGCTTAATTTGC | pJL89 cDNA2 | 3’ end of the IR |
| MP(fs) Fwd | ATGGTCTTTCTACCAATAACAATTCTTCTC | pJL89 cDNA2 | 5’ end of the MP gene |
| MP(fs) Rev | CTCAACATAAAATTCACCATTGATTTAC | pJL89 cDNA2 | GJ between p61 and MP genes |
| p24 (fs) Fwd | AAGGTTGGCAACGGAAAGTTTCG | pJL89 cDNA2 | 5’ end of the p24 gene |
| p24(fs) Rev | ATATCTTTGCCTAACGTTAGCTGC | pJL89 cDNA2 | 5’ end of the p24 gene |
| Δp15 XbaI Fwd | TATCTAGATTGTCATCTCGCTAGCACA | pJL89 cDNA2 | After p15 stop codon |
| Δp15 NcoI Rev | TACCATGGTGTAATGTTCACTAATAATTGAGAGTTATTAGCC | pJL89 cDNA2 | Before p15 start codon |
| Δp61 (BsaI) NheI Fwd | A*GGTCTC*GCTAGCATCAATGGTGAATTTTATGTTG | pJL89 cDNA2 | After p61 stop codon |
| Δp61 (BsaI) NcoI Rev | A*GGTCTC*CCATGGTTGACAATACTATTTTAGCTTAATTTGC | pJL89 cDNA2 | Before p61 start codon |
| ΔMP Fwd BspHI | TATCTAGAATGGACGCTCAACTTCTACAAG | pJL89 cDNA2 | After val codon at position 288 of MP |
| ΔMP NcoI Rev | TACCATGGCTCAACATAAAATTCACCATTGAT | pJL89 cDNA2 | Before MP start codon |
| Δp24 XbaI Fwd | TATCTAGACACCTACTGGTGTTATGCG | pJL89 cDNA2 | After p24 stop codon |
| Δp24 NcoI Rev | TACCATGGTTATTCGCTTGTAGAAGTTGAGC | pJL89 cDNA2 | 3' end of the MP gene |
| DsRed BsaI (BspHI) Fwd | A*GGTCTCTC*ATGAGGTCTTCCAAGAATGTTATC | pSK | 5' end of the DsRed gene |
| DsRed BsaI (XbaI) Rev | A*GGTCTC*TCTAGACTAAAGGAACAGATGGTGGC | pSK | 3' end of the DsRed gene |
| Δp29 BsaI Fwd | AGGTCTCGTCGGTGCGATTAATGATGG | pJL89 cDNA1 | 5' end of the CiLV-C RNA1 3UTR |
| Δp29 BsaI Rev | AGGTCTCAATGACAAAATACAAATTCGGTAA | pJL89 cDNA1 | 3’ end of the gene junction (GJ) |
| TCV CP BsaI (TCAT) Fwd | A*GGTCTC*GTCATTATGGAAAATGATCCTAGAGTCC | pTCV | 5' end of TCV CP |
| TCV CP BsaI (CCGA) Rev | A*GGTCTC*ACCGACCTAAATTCTGAGTGCTTGC | pTCV | 3' end of TCV CP |
| cDNA1-p29:HA.3' Fwd | TACCCATACGATGTTCCAGATTACGCTTAGGTCGGTGCGATTAATGATGGG | pJL89 cDNA1 | CiLV-C RNA1 3 UTR |
| cDNA1-p29:HA.3' Rev | CTGCGCTGAGTCGGAGTCATC | pJL89 cDNA1 | 3' end of the p29 gene |
| cDNA1:HA:p29.5' Fwd | ATGTACCCATACGATGTTCCAGATTACGCTAGTATCGTAACTTTCACTTTGAC | pJL89 cDNA1 | 5' end of the p29 gene |
| cDNA1-HA:p29.5' Rev | AATGACAAAATACAAATTCGG | pJL89 cDNA1 | p29 GJ |
| cDNA1-GFP BsaI (NcoI) Rev | A*GGTCTC*CCATGGAATGACAAAATACAAATTCGG | pJL89 cDNA1-GFP | Before GFP start codon |
| cDNA1-GFP BsaI (NheI) Fwd | A*GGTCTC*GCTAGCAGTAATACTGACTTTTATGATACTATT | pJL89 cDNA1-GFP | After GFP stop codon |
| CiLV-C MP BsaI (NcoI) Fwd | A*GGTCTCCC*ATGGCTCTTTCTACCAATAAC | CiLV-C MP | 5' end of CiLV-C MP |
| CiLV-C MP BsaI (NheI) Rev | A*GCTCTC*GCTAGCTTATTCGCTTGTAGAAGTTGAG | CiLV-C MP | 3' end of CiLV-C MP |
| TMV MP BsaI (NcoI) Fwd | A*GCTCTCCC*ATGGCTCTAGTTGTTAAAG | TMV MP | 5' end of TMV MP |
| TMV MP BsaI (NheI) Rev | A*GCTCTC*GCTAGCTTAAAACGAATCCGATTCGGCG | TMV MP | 3' end of TMV MP |
| VIGS eIF4A Fwd | GGGGACAAGTTTGTACAAAAAAGCAGGCTTCTATGCTTATGGTTTTGAGAAGCC | NbeIF4A | 167-188 position of NbeIF4A gene |
| VIGS eIF4A Rev | GGGGACCACTTTGTACAAGAAAGCTGGGTGTTAAAGGATACGCTGATCCTCACG | NbeIF4A | 445-465 position of NbeIF4A gene |
| eIF4A qPCR Fwd | AATGACAGAGCTGCTTGGTAC | NbeIF4A | 57-77 position of NbeIF4A gene |
| eIF4A qPCR Rev | AGGTTTTCTTGCAAACCCAT | NbeIF4A | 133-152 position of NbeIF4A gene |
| L23 qPCR Fwd | AAGGATGCCGTGAAGAAGATGT | 60S ribosomal protein |  |
| L23 qPCR Rev | GCATCGTAGTCAGGAGTCAACC | 60S ribosomal protein |  |

The italicized sequence corresponds to the *Bsa*I restriction site.

Underlined sequences indicated i) restriction sites *Nco*I, *BspH*I, *Nhe*I, or *Xba*I; ii) overlapping sequence, iii) HA sequence, or iv) attB sequences.
