# Supplementary figures and images for: Correction: From darkness to light: Genetic manipulation of an atypical plant virus unveils key insights into kitavirus biology, highlighting capsid protein and eIF4A engagement to drive viral infection
Source: PLoS Pathog. 2025 Nov 21;21(11):e1013705. doi: 10.1371/journal.ppat.1013705 (PMC12637930; doi:10.1371/journal.ppat.1013705)

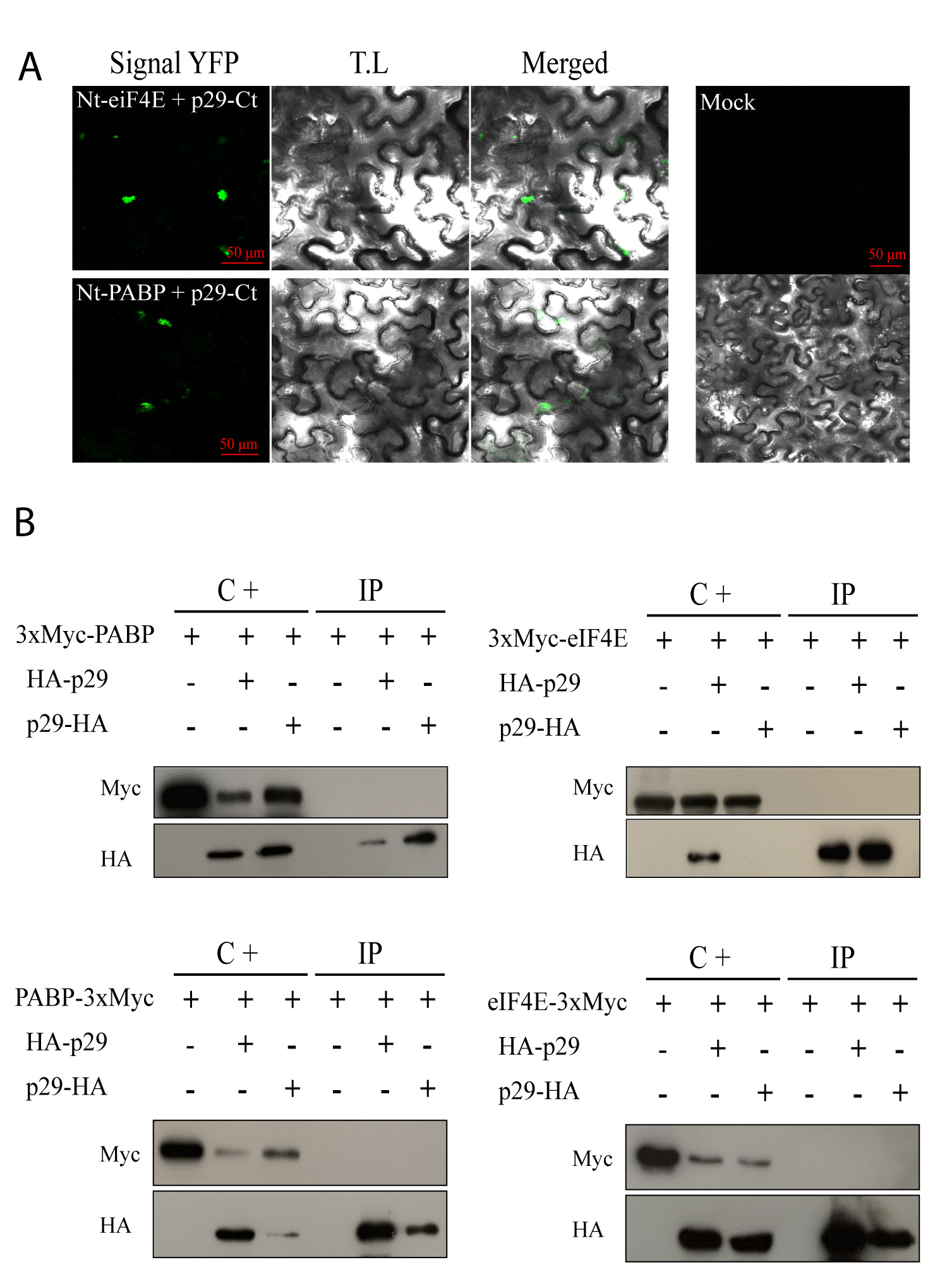

Supplement: S11 Fig — (A) In vivo interaction between the p29 and eIF4E/PABP. PABP and eIF4E were targeted at their C-terminus with NYFP or CYFP, respectively, and co-expressed with p29 fused at its N- or C-terminus with the NYFP or CYFP. A representative protein pair combination is indicated at the top of the image. The image shows the reconstitution of the YFP fluorescence distributed through the cell cytoplasm of N. benthamiana cells. The image is representative of several infiltrated leaves from three different plants. The fluorescent signals were captured at 3 dpi. The green (GFP), transmitted light (T.L) channels, and merged images are shown in the figure. The negative control is illustrated by a representative image (mock) showing the expression of eIF4A, eIF4E, and PABP combined with Ncyt constructs and p29 combined with the Cer construct. The bars indicate 50 μm. (B) CoIP of CiLV-C p29 with eIF4E and PABP. Extracts of N. benthamiana leaves expressing p29 fused at its N- or C-terminus with an HA epitope and 3xMyc targeted eIF4E or PAPB at their N- or C-terminus, were analyzed at 3dpi. The CoIP assay was addressed using the Pierce HA-Tag Magnetic IP/CoIP Kit. Western blot analysis was carried out using Myc and HA antibodies. C + , positive control (non-immunoprecipitated samples); IP, immunoprecipitated samples. ‘+’ and ‘–‘ signs indicate the presence or absence of the corresponding proteins in the leaf extracts. (TIF) [file ppat.1013705.s001.tif]
